# *PKD1* upstream open reading frames affect Polycystin-1 expression and polycystic kidney disease phenotypes

**DOI:** 10.1172/JCI203177

**Published:** 2026-07-28

**Authors:** Zhigui Li, Zi Guo, Soyoung Cho, Rishi Bhardwaj, Ke Dong, Sorin Fedeles, Whitney Besse

**Affiliations:** Department of Internal Medicine, Section of Nephrology, Yale School of Medicine, New Haven, Connecticut, USA.

**Keywords:** Genetics, Nephrology, Genetic diseases, Mouse models, RNA processing

## Abstract

Autosomal dominant polycystic kidney disease (ADPKD) accounts for 5%–10% of prevalent end-stage kidney failure (ESKD). ADPKD cysts result from a loss of sufficient functional expression of *PKD1*/Polycystin-1 (PC1) in approximately 80% of families. Kidney disease severity correlates with the extent to which PC1 dosage is reduced below a critical level, and evidence suggests therapeutic benefit from increasing PC1 expression in these conditions. Upstream open reading frame (uORF) translation can reduce translation of a protein’s coding sequence. Ribosome profiling data and bioinformatic predictions suggested the presence of conserved *PKD1* uORFs, so we sought to explore their biological role. We generated luciferase reporters and two humanized *PKD1* 5’ UTR mouse models with or without single nucleotide edits removing uORF start codons (ΔuORF) to define active uORFs and test their impact on PC1 translation. *PKD1* uORF start codons can robustly initiate translation, and ΔuORF conveys a 2–4 fold increase in PC1 protein expression and resultant prevention of kidney cysts in *Dnajb11* as well as in *Pkd1* missense models. *PKD1* uORF1-blocking steric antisense oligonucleotides (ASOs) substantially increase PC1 expression in vitro. *PKD1* uORFs play an important role in the low basal expression of WT *PKD1*, and their inhibition represents an opportunity to therapeutically increase PC1 translation in polycystic kidney and liver disease resulting from reduced dosage of PC1.

## Abstract 2

We demonstrate that upstream protein translation on the PKD1 transcript blunts Polycystin-1 expression and can be blocked to therapeutically increase expression in polycystic kidney disease

## Graphical abstract

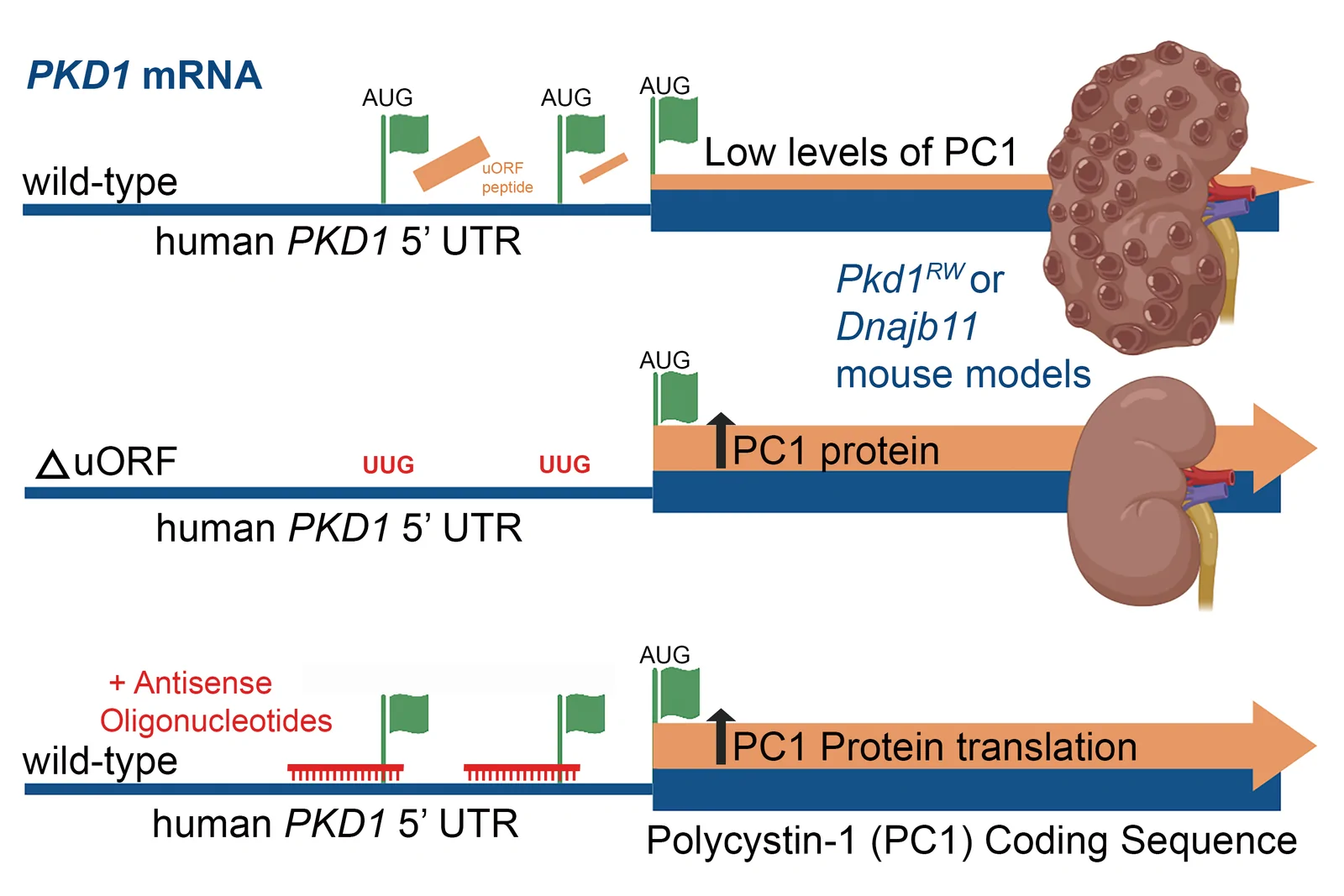

## Introduction

Autosomal dominant polycystic kidney disease (ADPKD) affects 1 in every 1,000 people, and accounts for 5%–10% of all end-stage kidney disease (ESKD) worldwide (1). ADPKD is characterized by fluid-filled cysts that arise from kidney tubule epithelial cells and bile duct epithelial cells in the kidney and liver respectively (1). These cysts increase in size and number over time resulting in organ enlargement and most notably kidney failure in the 4^th^ to 7^th^ decade of life (1–3). In a subset of patients, liver cysts can result in painful hepatomegaly with complications that may require partial or total hepatectomy and liver transplant (4–6). A mechanistically related phenotype of autosomal dominant polycystic liver disease (ADPLD), also known as isolated polycystic liver disease (PCLD), can have equally severe liver cysts with few or no kidney cysts (7, 8). The only FDA-approved drug for these polycystic diseases, the vasopressin receptor antagonist Tolvaptan approved for ADPKD, carries a risk profile and only modestly delays kidney disease progression by slowing cyst proliferation and secretion (9, 10). An unmet need exists to identify and develop effective therapies to delay or prevent ongoing cyst initiation and growth in the kidney and liver (11).

The “master regulator” of cyst formation is the large 4,303 amino acid primary cilium-localized transmembrane protein polycystin-1 (PC1), encoded by the *PKD1* gene (12). Heterozygous truncating or deleterious nontruncating variants in *PKD1* cause at least 78% of ADPKD families (13). Such variants in PC1’s binding partner *PKD2*/Polycystin-2 (PC2) or other genes involved in PC1 maturation — many associated with the ADPLD phenotype — explain the remainder (12–22). The unifying disease mechanism is insufficient functional PC1 at its site of action on the primary cilium of epithelial cells. Insufficient functional PC1/PC2 complex results in altered proliferative and fluid secreting properties in affected cells, leading to development of cysts (23–28). Genetic analysis of cyst epithelium suggests that the initiating factor for each cyst is often a somatic mutation (17% nontruncating, others truncating or gene rearrangements) to the remaining WT allele resulting in the recessive genotype (28, 29). A dosage model is proposed in parallel, whereby either minor change in the second *PKD1* allele or additional factors in the microenvironment or gene regulation may reduce PC1 dosage to cross a cystogenic threshold (30–33). Kidney disease severity correlates with the degree of PC1 dosage loss (30, 31, 34, 35). There are 2 established mechanisms of reduced PC1 dosage: (a) missense mutations in *PKD1 —* either as the germline or somatic mutation *—* that result in inefficient PC1 maturation, or (b) a biogenesis defect of PC1 due to loss of one of the alternative disease genes, including *GANAB*, *DNAJB11*, *ALG9*, *PRKCSH*, and *SEC63* (12, 18, 20, 31).

Animal models, and an early human clinical trial, support the premise that recovering PC1 dosage can prevent or rescue cysts. Animal models assessed for *PRKCSH* or *SEC63* show that 3 extra genomic copies of the *Pkd1* gene was enough to overcome the inefficiency in PC1 maturation and thereby prevent cyst formation (12). Animal models that prevent microRNA-17 repression of *PKD1* mRNA rescue kidney cysts that result from the commonly studied *PKD1* p.R3277C missense variant, and recent clinical trials show short term safety and efficacy of antisense oligonucleotide-mediated inhibition of microRNA-17 (36, 37). Increasing relevant ER chaperone expression with XBP1s transgene to help PC1 maturation or stability reduces cyst severity in biogenesis models and in a *Pkd1* p.R2216W missense mouse model (orthologue of human *PKD1* p.R2220W) (38, 39). Encouragingly, recent animal studies suggest that restoration of sufficient PC1 function even after cyst formation can be sufficient to restore normal kidney architecture and function (40).

PC1 is expressed ubiquitously across cell types and organs, yet its expression level in the disease-relevant kidney and liver is low, particularly after development (41–43). Recovering higher protein synthesis of *PKD1*/PC1 is a key therapeutic goal in ADPKD and ADPLD. Untranslated regions (UTRs) are an increasingly recognized effector of protein synthesis (44, 45). Translation of peptides starting at initiation codons in the 5’ UTR, known as upstream open reading frames (uORFs), can affect rates of protein synthesis from the main coding sequence (CDS) in ways affected by distance between the uORF stop codon and the CDS, mRNA secondary structure, and cell context affecting rate of preinitiation complex (PIC) reassembly after uORF stop codon (46–48). While there are functional roles established for a small number of uORF-encoded peptides, it is suggested that the relevance of the majority of studied uORFs is on coding sequence translation (49–52). Targeting UTRs to increase protein synthesis is an approach being tested in several diseases, including kidney disease (53–55). We noted that the *PKD1* gene contains predicted uORFs that are among the top 0.1% most likely to be translated based on a computational scoring of all predicted uORFs across the genome (56). We hypothesized that *PKD1* uORFs were likely translationally active and contributing to the low level of PC1 protein in WT cells. We tested the role of *PKD1* uORFs on PC1 protein expression to define biological mechanism and establish a potential therapeutic approach for increasing Polycystin-1 dosage.

## Results

### Defining PKD1 uORFs.

**Figure 1:**
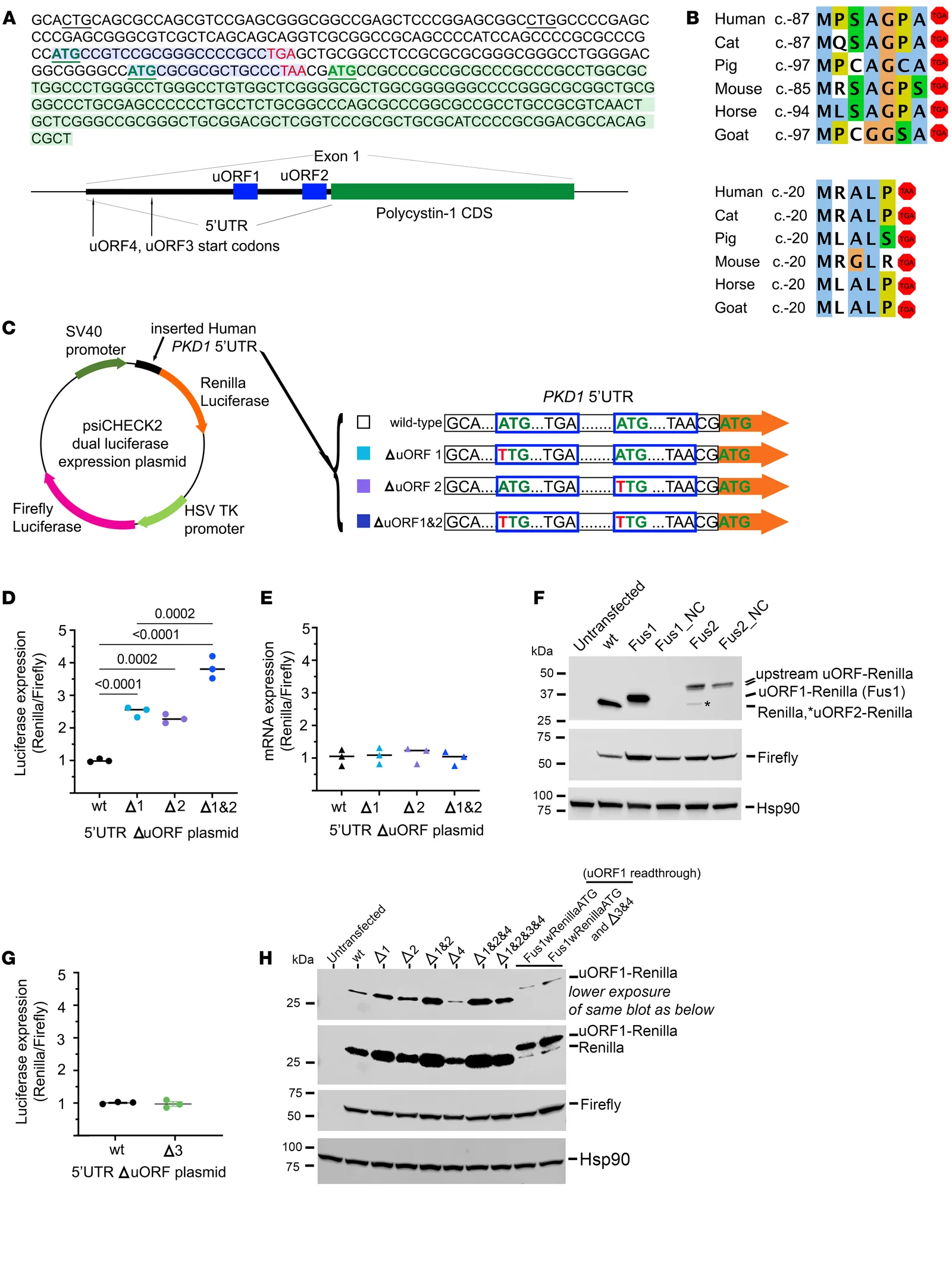
*PKD1* 5′ UTR has active uORFs that affect Polycystin-1 protein translation. (**A**) DNA sequence of human *PKD1* exon 1. Green shading indicates the coding region (CDS) of Polycystin-1. The preceding sequence is the 5’ UTR. Potential start codons are underlined, including the only 2 ATG sequences at the c.–87 and c.–20 position. These ATG start codons initiate the proposed upstream open reading frames highlighted in blue, which we named uORF1 and uORF2. (**B**) Orthologues of *PKD1* uORF1 and uORF2. (**C**) Illustration of psiCHECK2 luciferase construct with either the WT *PKD1* 5′ UTR sequence (“wt”), or versions with single nucleotide edit(s) of uORF1 or uORF2 start codons as indicated. Firefly luciferase expresses independently from the same construct to serve as an internal control for transfection efficiency. (**D**) Comparison of Renilla/Firefly protein expression detected by luciferase quantification for HEK293 cells transfected with luciferase construct of the indicated *PKD1* 5’ UTR variant. (**E**) No difference between Renilla/Firefly mRNA expression of the different constructs. (**F**) Anti-Renilla immunoblot of lysate from HEK293 cells transfected with the indicated constructs (Supplemental Figure 4 and Supplemental Table 1) that express a fusion protein of uORF1-Renilla (“Fus1”) or its negative control, or uORF2-Renilla (“Fus2”) or its negative control. The fusion protein constructs contain the minimum edits to remove the ATG of Renilla and put the uORF initiation site in frame. (**G**) Comparison of Renilla/Firefly expression from WT luciferase construct versus ΔuORF3, which only differs by c.A>T at c.–161. (**H**) Anti-Renilla immunoblot of lysate from HEK293 cells transfected with the indicated constructs (Supplemental Table 1). One-way ANOVA was applied in **D** and **E**, and 2-tailed *t* test in **G**. Significant differences are indicated.

The human *PKD1* 5’ UTR is 209 nucleotides in length and contains two occurrences of the ATG (AUG in mRNA) canonical start codon sequence 87 and 20 nucleotides before the start codon (c.–87 and c.–20, respectively) relative to the CDS start (Figure 1A). Translation from these potential start codons would encode a 7 or 5 amino acid peptide, respectively, before reaching a stop codon prior to CDS start. We defined these as “uORF1” and “uORF2.” Both uORFs and the *PKD1* coding sequence have moderate strength Kozak sequences (57). Both uORFs are present in the genomic sequence of the 4 of 6 human *PKD1* pseudogenes that contain the 5’ UTR but with 2 nucleotide differences in uORF1. Comparison of defined *PKD1* 5’ UTR sequences across mammalian species shows a conserved presence of ATG-initiated uORFs at near identical spacing from the CDS start and modestly conserved amino acid sequence (Figure 1B). The nucleotide sequence of uORF1 and uORF2 including start and stop codons is indeed more highly conserved than the remaining nucleotide sequence of the human 5’ UTR (“non-uORF”) (Supplemental Figure 1; supplemental material available online with this article; https://doi.org/10.1172/JCI203177DS1).

### PKD1 uORFs affect CDS translation: luciferase reporter assay.

We hypothesized that *PKD1* uORF1 and uORF2 contribute to the functionally borderline expression of Polycystin-1, which falls below a cystogenic threshold in patients with ADPKD and ADPLD. To assess the effect of *PKD1* uORF1 and uORF2 on protein expression, we inserted the 209-nucleotide human *PKD1* 5’ UTR — either WT or uORF-edited — preceding Renilla luciferase coding sequence in a psiCheck2 dual luciferase expression plasmid (Figure 1C and Supplemental Table 1). To test the effect of blocking translation from uORF1, “ΔuORF1” contains a single A>T edit to the A of uORF1’s start codon, and, similarly, “ΔuORF2” and “ΔuORF1&2” contain the respective A>T edits to these start codon(s). We transfected independent wells of HEK293 cells with equivalent preparations of luciferase plasmid in biological triplicate for each construct and quantified Renilla luciferase expression relative to an independently expressing Firefly luciferase internal control (Figure 1C). Compared with the plasmid with WT *PKD1* 5′ UTR, ΔuORF1 or ΔuORF2 showed 2.5 fold and 2.3 fold increased Renilla/Firefly expression, respectively, and ΔuORF1&2 showed a 3.8 fold increase (Figure 1D). The Firefly internal control showed highly consistent transfection rates (Supplemental Figure 2). Renilla mRNA expression in biological triplicates showed no difference between WT and ΔuORF constructs consistent with the effect being at the level of protein translation (Figure 1E). RNA secondary structure predictions within the limitations of available programs and input parameters are without apparent change (Supplemental Figure 3) (58–61). Taken together, these data support a 2–4 fold increase in CDS protein expression by genetic edit to remove *PKD1* uORF start codons.

### PKD1 uORF1 and uORF2 can initiate translation: fusion protein analysis.

To confirm the protein translation beginning at uORF1 and uORF2 initiation codons, which is fundamental to the proposed mechanism of effect, we designed a system to overcome the technical challenge of detecting the 5 and 7 amino acid uORF peptides. We edited the uORF stop codons in our *PKD1* 5′ UTR dual luciferase construct such that the uORF1- and uORF2-initiated peptides extend to be an in-frame fusion protein with approximately 35 kDa Renilla protein (“Fus1,” “Fus2,” respectively). Fus2 required an additional nucleotide to put uORF2 in frame with Renilla, and both constructs had Renilla’s start codon edited to TTG so the Renilla peptide sequence would only be translated if translation initiation occurred from a uORF start codon. Negative control constructs “Fus1-NC” and “Fus2-NC” have the single nucleotide A>T edit compared with their respective fusion construct to lack of that specific uORF’s start codon. Construct sequence is provided in Supplemental Figure 4A and Supplemental Table 1. We transfected independent wells of HEK293 cells with these plasmids and compared Renilla fusion protein expression and migration by Western blot. Anti-Renilla Western blot shows robust translation of uORF1, as represented by the approximately 37 kDa uORF1-Renilla fusion protein expressed from the Fus1 construct, and detectable weak translation of uORF2, as represented by the approximately 36 kDa uORF2-Renilla fusion protein expressed from Fus2, indicated by an asterisk (Figure 1F). Notably, Fus2 also expresses an approximately 42 kDa larger band with stronger expression than uORF2, suggesting additional in-frame translational initiation further upstream in the *PKD1* 5’ UTR; we investigate this below. Most notably, this experiment confirms strong translation initiation from uORF1 and some from uORF2.

### Public datasets support PKD1 uORF translation, and consideration of “uORF3”.

Given evidence of additional *PKD1* uORF translation from further upstream in the 5′ UTR, we next considered publicly available ribosome profiling (Ribo-seq) data in the Genome Wide Information on Protein Synthesis though visualization of ribosome profiling data (GWIPS-viz) browser (62, 63). Read depth in *PKD1* exon 1 is poor and inconsistent across the region, which greatly limits interpretations, nonetheless, there are ribo-seq reads in the human and mouse 5′ UTR, suggesting translation from uORFs with greatest read depth in the region of uORF1 (Supplemental Figure 5). An independent “Ribo-uORF” resource shows 8 datasets supporting *PKD1* uORF translation with 11–20 reads each (64, 65). Six out of the 8 datasets support only uORF1 translation initiation, while the 2 others also suggest an upstream CTG-initiated uORF at the c.–161 position or c.–206 position. We named these uORF3 and uORF4, respectively. As uORF3 at c.–161 has the strongest Kozak sequence among the potential uORFs (56) we hypothesized it may also affect CDS translation. However, upon designing and testing a ΔORF3 luciferase construct we found that Renilla/Firefly expression was no different from that of the WT construct (Figure 1G and Supplemental Table 1).

### Four PKD1 uORFs translate and affect the use of downstream start codons.

The upper band expressed from Fus2 in Figure 1F suggests active translation from at least one uORF upstream of uORF1 and uORF2. We focused on the potential uORFs that would start with CTG (CUG) codon at c.–161 and c.–206 that we define as uORF3 and uORF4, respectively. These are in frame with uORF2 and use its stop codon if translated, and there are no other ATG, CTG, or GTG start codons upstream in the *PKD1* 5′ UTR. We edited the start codons of uORF3 or uORF4 with or without additional uORF edits in the Fus2 construct to compare the resultant Renilla fusion proteins on Western blot. The Fus2-ΔORF4 and Fus2-ΔORF3 edits reduce expression of the upper and lower components of the 42 kDa wide band expressed by Fus2, thus supporting the proposed start codons and demonstrating active translation from uORF3 and 4 (Supplemental Figure 4, B and C). It is unclear why changing CTG to TTG does not fully abolish uORF4 translation; nonetheless, reducing translation of the most upstream uORF (“uORF4”) results in more apparent translation of the next uORF (“uORF3”) (Supplemental Figure 4C), while reducing translation of both uORF4 and uORF3 increases translation of the next most 5’ uORF, which is uORF1 (Figure 1H, rightmost lane), and following the same 5′ to 3′ order, impairing translation from uORF4, uORF3, and uORF1 maximally increases translation of the final uORF (uORF2, Supplemental Figure 4C, rightmost lane). With this understanding from Fus2 constructs, we made a final set of luciferase constructs to determine the optimal uORF inhibition strategy to maximize CDS translation (Figure 1H).

We conclude that uORF1 translation has the strongest inhibitory effect on CDS protein expression. Upstream uORFs are active; however, their inhibition may only increase uORF1 translation, which is counterproductive. Our data suggest that uORF2 translation may be low at baseline but increases substantially in the setting of inhibition of uORF1 (Supplemental Figure 4C), thus demonstrating a mechanism by which uORF2 inhibition could have additive benefit to uORF1 inhibition alone. We considered whether inhibition of all 4 uORFs or only the most active (uORF4, uORF1, uORF2) would achieve the maximal benefit; yet, we find that there is no added increase over inhibition of just uORF1&2 (Figure 1H). Given that the most relevant human *PKD1* uORF1 is in frame with the CDS, we considered whether readthrough of the uORF1 stop codon would be a strategy worth pursuing — with the large caveat that functional testing of a 5′-extended PC1 protein would be necessary, as this may likely affect the PC1 signal peptide and therefore protein maturation and trafficking. We tested a version of our Fus1 model with intact Renilla start codon (“Fus1wRenillaATG”) to compare intensity of translation start from uORF1 versus the CDS start and found that, indeed, uORF1 translates more strongly than the CDS. However, the summative intensity of both bands appeared lower than translation from the CDS start with uORF1&2 inhibition, so we pursued strategies to inhibit translation from uORF1 and uORF2.

### ASOs targeting PKD1 uORFs substantially increase CDS protein expression in a sequence-specific manner.

**Figure 2:**
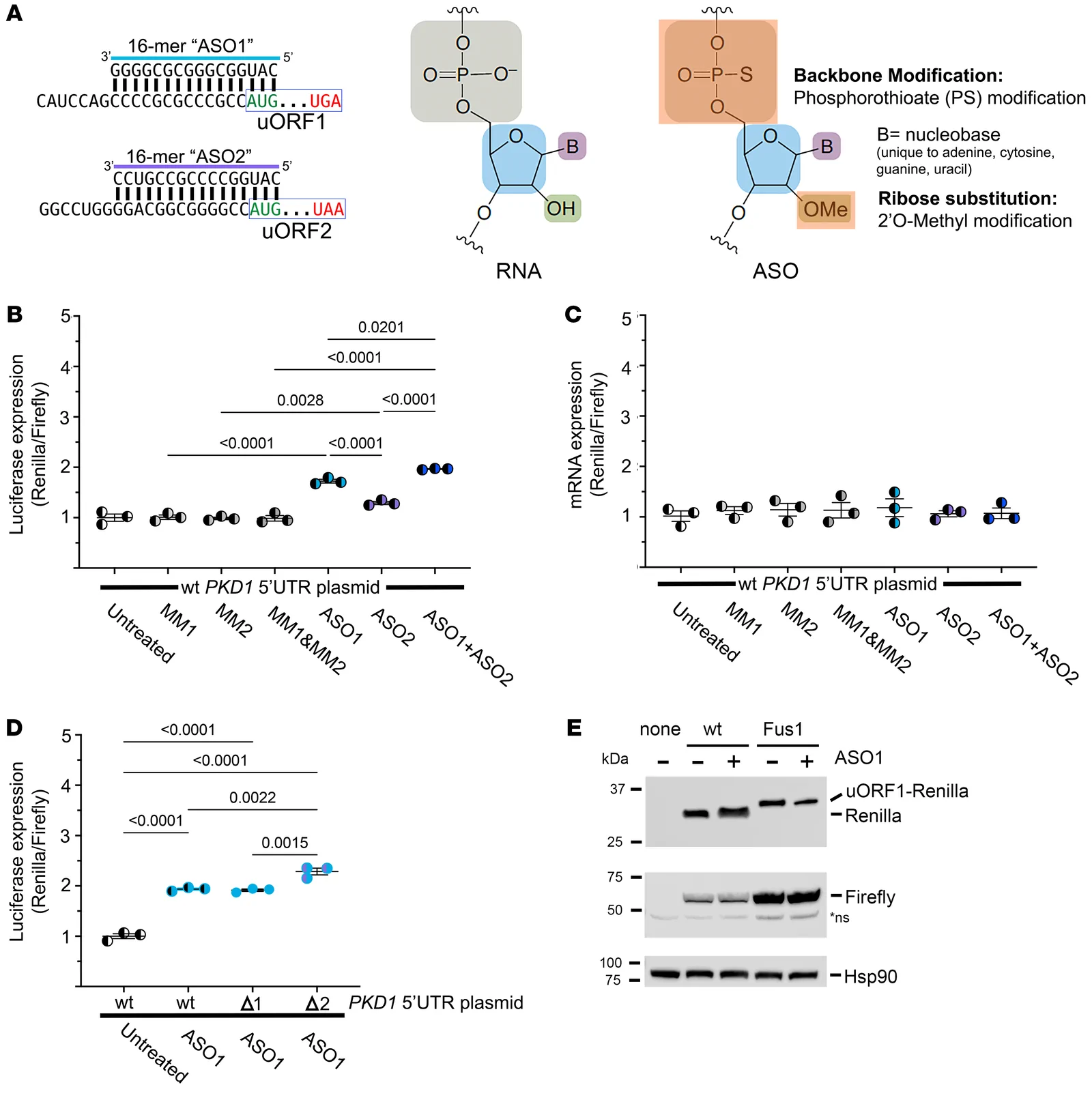
Design and efficacy of antisense oligonucleotides (ASOs) Inhibiting uORF translation to increase protein expression post-transcriptionally. (**A**) Illustration of the 16-nucleotide ASO sequences complementary to human *PKD1* 5’ UTR mRNA sequence used in this study. ASO nucleotides are a derivative of RNA with PS linkages and 2’OMe modifications on all nucleotides as highlighted in orange on illustration modified from Roberts et al. (68). (**B**) Effects of 20 nM ASOs on Renilla/Firefly expression in WT *PKD1* 5’ UTR dual-luciferase assay assessed after 24 hours of treatment. Mismatched control ASOs (MM1 and MM2) are of identical chemical composition to ASO1 and ASO2 but with every third nucleotide modified. (**C**) No difference between Renilla/Firefly mRNA expression of the transfected cells treated with or without treatment with ASOs. (**D**) ASO1 treatment of Luciferase construct variants illustrates that the ASO only has additional benefit when added to the construct that contains the uORF1 initiation codon (WT or DuORF2, but not DuORF1). Data represent 3 independent treatments for each experimental condition. (**E**) Anti-Renilla immunoblot showing that the effect of ASO1 on protein expression is specific to the location of the protein initiation. As designed, ASO1 indeed inhibits translation initiation from uORF1 (the only initiation codon for Renilla in the uORF1-Renilla fusion protein “Fus1” construct) even though it increases protein translation from the downstream typical ATG initiation codon of Renilla in the WT construct, as identified in this study. One-way ANOVA was applied in **B**–**D**. Significant differences are indicated.

We designed 16-nucleotide ASOs and mis-matched controls to block translation initiation from uORF1 or uORF2 in human or mouse based on successful ASO sequence designs for uORF blocking in other genes (66, 67) and chose ASO chemistries used in current FDA-approved therapies with established safety and stability profiles (68). We call these “ASO1,” “ASO2” (Figure 2A), “mASO1,” and “mASO2” (Supplemental Figure 6 and Supplemental Table 2). ASO treatment to HEK293 cells expressing “WT” luciferase plasmid demonstrates increased Renilla luciferase (protein) expression, without affecting Renilla mRNA expression (Figure 2, B and C). ASO-based uORF inhibition was more impactful with ASO1 than ASO2, although modest additive benefit was seen from ASO2. To demonstrate specificity of the ASO1 effect to the uORF translation as opposed to other factors related to mRNA binding, we combined the genetic edit and ASO treatment. We found that ASO1 treatment has no added benefit on a construct that already lacks uORF1 start codon, but can treat the ΔuORF2 construct to achieve protein expression similar to combined ASO1 and ASO2 treatment (Figure 2D). To visualize ASO1’s specific inhibition of uORF1 translation, we applied ASO1 to the uORF1-Renilla fusion protein “Fus1” construct. We found that ASO1 treatment increased translation from the main CDS while it reduced — although not completely — translation initiated at uORF1, as represented by the uORF1-Renilla fusion protein (Figure 2E).

### Native PKD1 translation is increased with PKD1 uORF-blocking treatment.

**Figure 3:**
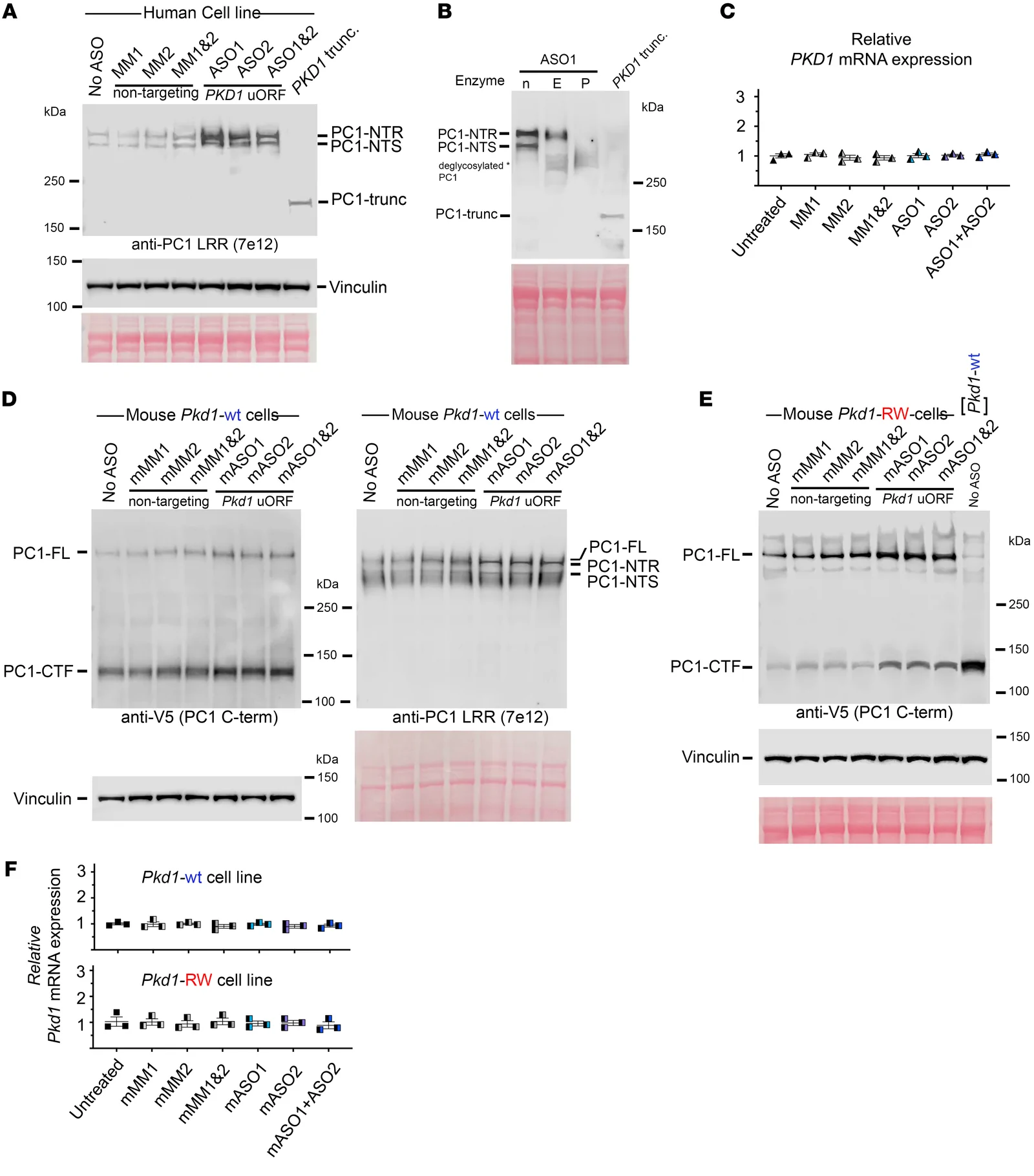
ASOs targeting *PKD1* uORFs increase Polycystin-1 protein expression in human and mouse cell lines without affecting mRNA expression. (**A**) Native PC1 immunoblot (PC1 N-term, 7e12) demonstrates increased PC1 with ASOs targeting *PKD1* uORFs in human retinal pigment epithelial (RPE) cells. (**B**) To confirm the identity of protein bands, protein lysates from RPE cells treated with ASO1 were subsequently treated with Endoglycosidase H (EndoH, “E”), PNGase F (“P”), or buffer only (“n”) before separation on the Tris-Acetate gel and blotting with anti-PC1 N-term antibody 7e12. We created a *PKD1-*null RPE clonal cell line (frameshift in exon 15) using CRISPR/Cas9 and ran lysates from this cell line (“*PKD1* trunc”) in parallel as a negative control for PC1 bands of native size. (**C**) *PKD1* mRNA corresponding to the experiment in **A**. (**D**) Anti-V5 immunoblot (left panel) and anti-PC1 N-terminal 7e12 blot (right panel) of lysates from an immortalized mouse cell line with C-terminal V5 tag on wild-type *Pkd1* (“*Pkd1-*wt*”)* treated with the indicated ASOs targeting mouse *Pkd1* 5’UTR to inhibit the mouse orthologues of uORF1, uORF2, or both (mASO1, mASO2, or mASO1&2, respectively), or non-targeting mismatch controls (mMM1, mMM2, mMM1&2). (**E**) Anti-V5 immunoblot of lysates from a similar V5-tagged immortalized mouse cell line with the cleavage-impaired *Pkd1* p.R2216W missense variant (orthologue of human *PKD1* p.R2220W) (“*Pkd1-*RW*”)* treated with the indicated mASOs. Lysate from the *Pkd1-*wt cell line tested in panel 3D is run on this blot to allow for comparison of the ASO-treated *Pkd1-*RW PC1-CTF intensity with that of the untreated wild-type cell line. (**F**) *Pkd1* mRNA expression for experiments in **D** and **E**. One-way ANOVA was applied in **C** and **F** however there are no significant differences.

We next applied ASO1 and ASO2 to culture media of human retinal pigment epithelial (RPE) cells to determine their treatment effect on PC1 protein expression. RPE cells are a well-studied ciliated epithelial cell line chosen for its detectable native PC1 expression levels. Cells treated with ASO1 or ASO2, particularly ASO1 alone, show a strong posttranscriptional increase in PC1 protein (Figure 3, A–C). We confirmed the identity of the most prominent PC1 band as the functionally relevant mature N-terminal fragment (PC1-NTR) by demonstrating a negative control cell line “PKD1-trunc” which lacks the PC1 bands of normal size and showing that the PC1-NTR band is EndoglycosidaseH (EndoH) resistant, a feature achieved when a protein traffics through the middle Golgi (Figure 3B). Given the similarities in human and mouse uORFs and the critical use of mouse models for ADPKD therapeutics, we next tested mouse *Pkd1* uORF inhibition on immortalized mouse cell lines of *Pkd1* WT or *Pkd1* p.R2216W cystogenic missense allele (mouse orthologue of human p.R2220W) with a knocked-in C-terminal V5 tag (38). The PC1 C-terminal fragment (PC1-CTF), which holds onto the mature N-terminal fragment by a noncovalent bond, is an established representation of functional PC1 dosage (12, 18). In both genotypes, treatment with mASO1, mASO2, or the combination shows an increase PC1-CTF, PC1-FL, and PC1-NTR protein, without change in mRNA (Figure 3, D–F). The magnitude of effect on protein synthesis was generally less for the mouse *Pkd1* uORF-targeting ASOs compared with those targeting human uORFs, although some experiments showed similar levels of effect (data not shown).

### In vivo role of PKD1 uORFs.

**Figure 4:**
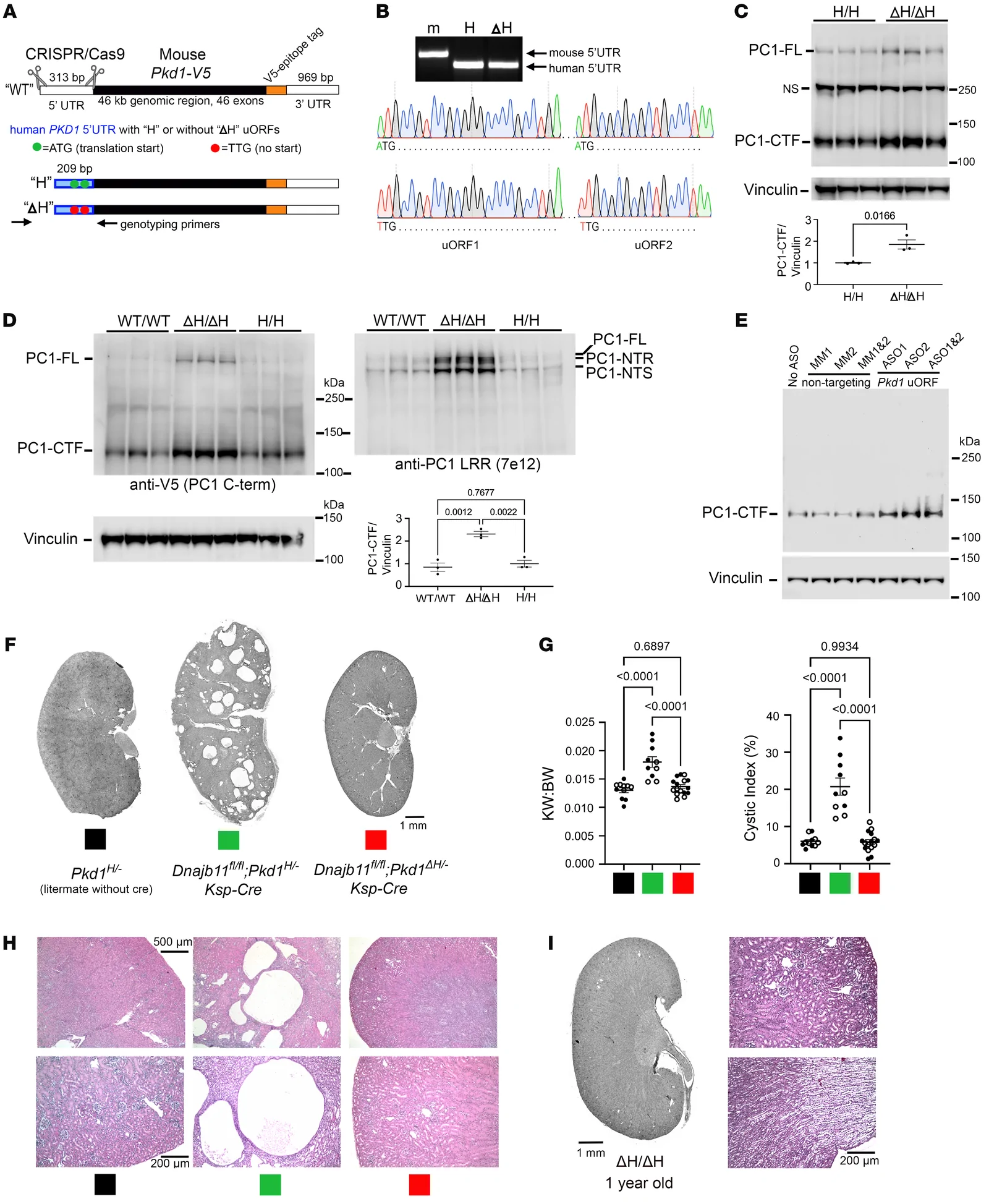
Humanized *PKD1* 5′UTR mouse model with or without *PKD1* uORFs. (**A**) Illustration of endogenous wild-type mouse *Pkd1* locus with previously knocked-in c-terminal V5 epitope undergoing CRISPR/Cas9-mediated replacement of the mouse 5′UTR with human wild-type or DuORF1&2 *PKD1* 5′UTR to make the “H” and “DH” alleles for this study. (**B**) Agarose gel electrophoresis illustrating the expected size difference of PCR amplicons between endogenous mouse *Pkd1* upstream-forward and exon 1-reverse primer pair. Sanger sequencing from homozygous mice with humanized 5′UTR alleles illustrates the two single nucleotides differences (the A>T in uORF1 and uORF2 start codons) which are the only differences between the H and DH alleles. (**C**) Anti-V5 immunoblot of P6 lung tissue lysate from H/H and DH/DH mice. NS, nonspecific band present using the rabbit host anti-V5 antibody optimal for tissue lysate. This band is no longer present when the immunoblot is preceded by anti-V5 immunoprecipitation (data not shown). (**D**) Anti-V5 (left panel) and anti-PC1 (N-term, 7e12, right panel) immunoblot of kidney primary cell lysate from the indicated genotypes. Vinculin denominator from lane 7 was used as denominator for all H/H sample quantification because Ponceau stain indicated equal loading. (**E**) Anti-V5 immunoblot of H/H primary cell lysate following 72 hours of treatment with the indicated human *PKD1* uORF-targeting ASOs. (**F**–**H**) Kidney histology scans, KW:BW, cystic index, and representative histology sections from 9-week-old mice. The cohort indicated by the black label are *Pkd1* heterozygous mice with *Dnajb11^fl/fl^* that lack Cre and therefore are noncystic controls (*Pkd1^H/–^* in **F** and **H**, and combined *Pkd1^H/–^* or *Pkd1*^DH/–^ in **G**. (**I**) Representative kidney histology of DH/DH mice aged to 1 year. Filled circle, male; empty circle, female. Additional data in Supplemental Figures 7 and 8. Two-tailed *t* test was applied in **C**, 1-way ANOVA was applied in **D** and **G**. Significant differences are indicated.

To test the role of human *PKD1* uORFs in vivo, we used CRISPR/Cas9 to replace the 313-nucleotide 5’ UTR of WT mouse *Pkd1-V5*(38) with the 209-nucleotide human *PKD1* 5’ UTR with or without the 2 A>T single nucleotide edits to remove uORF1 and uORF2 start codons (Figure 4A). The resultant alleles bred to homozygosity produce healthy WT mice of confirmed sequence (Figure 4B). We predicted that the mice without *PKD1* uORF1&2 start codons (*Pkd1^ΔH/ΔH^*) would have higher PC1 protein expression than mice with WT *PKD1* uORFs (*Pkd1^H/H^*) throughout the body. Native PC1 expression is known to be challenging to detect but is highest in lung tissue. Anti-V5 immunoblot on lung tissue supports increased PC1 expression in *Pkd1^ΔH/ΔH^* mice (Figure 4C). To best characterize the effect of this allele in the kidney, we cultured primary cells from postnatal day 9 (P9) mouse kidney in epithelial cell–promoting media. Figure 4D shows that expression of PC1 was similar between alleles with mouse (*Pkd1^WT/WT^*) or human (*Pkd1^H/H^*) WT 5′ UTR, but markedly higher — both in total and cleaved PC1 by C-terminal and N-terminal blots — when uORFs were edited (*Pkd1^ΔH/ΔH^*). Further, *Pkd1^H/H^* primary cells treated with our on-target ASOs showed a clear increase in PC1-CTF (Figure 4E).

### PKD1 uORF-blocking genetic edit rescues dosage-sensitive cysts: proof of concept for uORFs as a therapeutic target.

Orthologous mouse models for ADPKD require biallelic mutation of the disease gene. We have recently characterized a *Pkd1* dosage–sensitive mouse model using the human ADPKD minor disease gene *DNAJB11* (19, 69). *Dnajb11* loss results in inefficient maturation of PC1 protein in the endoplasmic reticulum, resulting in cysts when the functional dosage of PC1 falls below a cystogenic threshold despite a WT *Pkd1* gene. *Dnajb11^fl/fl^,* with the distal-nephron Ksp(Cdh19) Cre is a model of dosage-sensitive cysts: *Dnajb11^fl/fl^*;*Pkd1^+/–^;Ksp-cre* is cystic, whereas *Dnajb11^fl/fl^;Ksp-cre,* which is *Pkd1^+/+^* is not (69). For this study, we first confirmed that, indeed, the humanization of the *Pkd1* 5′ UTR did not change the model. We found that, while *Dnajb11^fl/fl^*;*Pkd1^H/–^;Ksp-cre* mice have a characteristic cystic phenotype, *Dnajb11^fl/fl^*;*Pkd1^ΔH/–^;Ksp-cre* mice appear entirely rescued from cyst formation (Figure 4, F–H, and Supplemental Figure 7), consistent with a greater than or equal to 2 fold increase in PC1 expression demonstrated from the *Pkd1^ΔH^* allele relative to *Pkd1^H^*. Of note, the breeding strategy allowed the represented genotypes to be litter mates.

### PKD1 uORF inhibition across the lifespan.

We hypothesize that the tiny 7 and 5 amino acid peptides translated from *PKD1* uORF1 and uORF2 do not themselves have a functional role; rather, the impact of *PKD1* uORFs is due to their effect on the availability of ribosomes for the translation of the PC1 coding sequence. To lend support for this hypothesis, we aged male and female *Pkd1^ΔH/ΔH^* mice *—* which would lack these uORF peptides and have the influence of uORF inhibition on PC1 expression throughout their life — to 1 year of age before sacrifice. Mice at this age appeared healthy, and, upon, sacrifice had normal kidney histology; kidney function is normal (Figure 4I and Supplemental Figure 8).

### Humanized PKD1 5′ UTR genetic edits on the Pkd1^RW^ missense allele.

**Figure 5:**
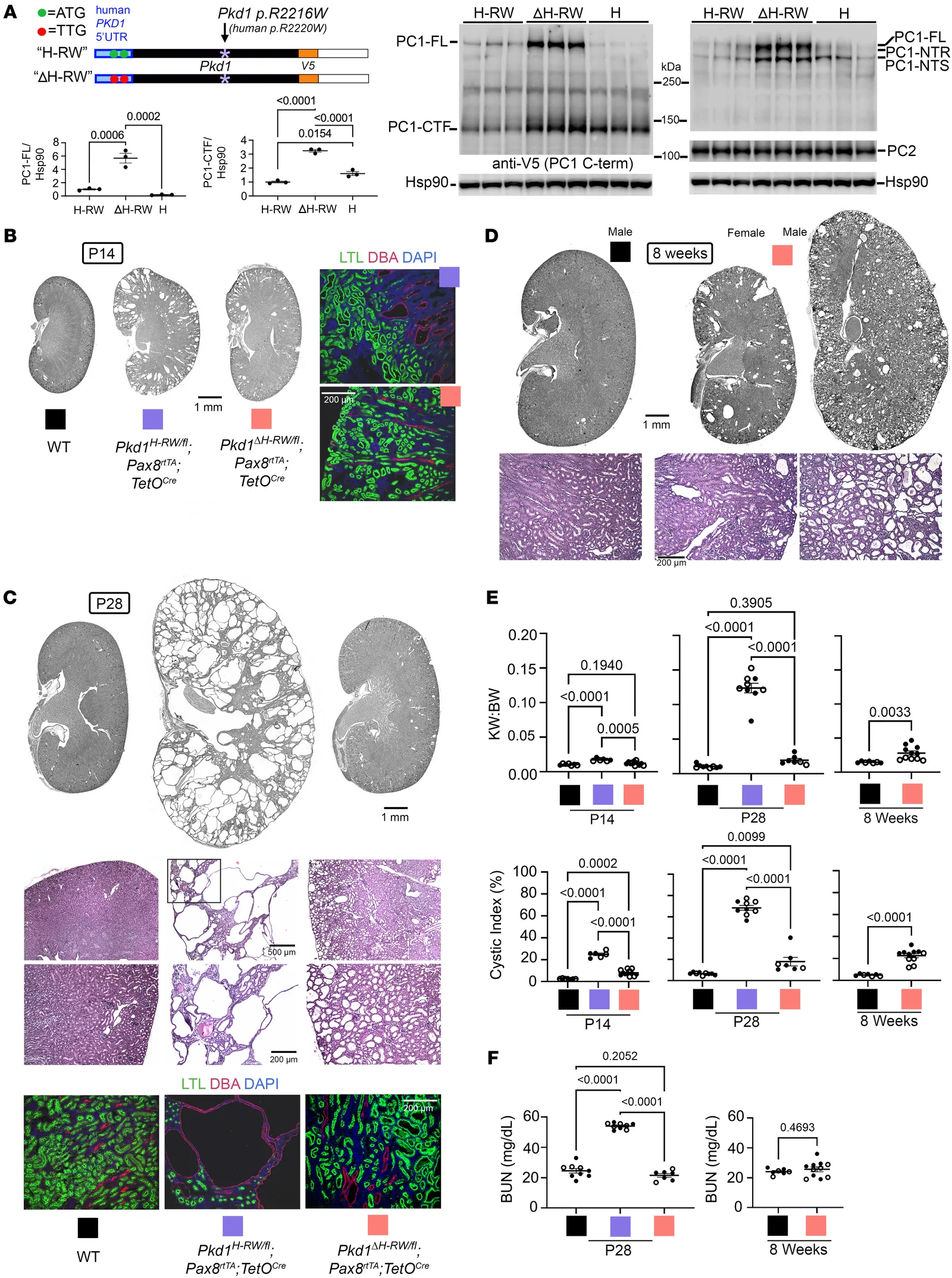
*PKD1* ΔuORF1&2 rescues cystic kidney models from the *Pkd1^RW^* missense allele. (**A**) Illustration of the humanized *PKD1* WT or ΔuORF1&2 5′UTR generated in this study on a *Pkd1*-V5 p.R2216W allele (orthologue of human p.R2220W). Anti-V5 immunoblot (center panel) and anti-PC1 (N-term, 7e12, right panel) immunoblot on P9 kidney primary cell lysate. Quantification is by densitometry of PC1-CTF and PC1-FL/Hsp90 on the anti-V5 blot. (**B**) Kidney histology and fluorescence imaging on kidney sections from mice of the indicated genotypes sacrificed at P14. (**C**) Kidney histology and fluorescence imaging from mice at P28. The black box indicates the region amplified in the higher power image. (**D**) Kidney histology from mice at 8 weeks of age. (**E**) KW-to-BW ratio and cystic index parameters from mice of the indicated genotypes sacrificed at age P14, P28, and 8 weeks. *Y*-axis labeling for the left panel applies across center and right panels for each parameter to allow comparisons across time points. (**F**) Blood urea nitrogen levels from mice of the indicated genotypes and time points. Filled circle, male; empty circle, female. Additional data are provided in Supplemental Figures 10–12. One-way ANOVA was applied in **A**, **E**, and **F** and 2-tailed *t* test for 8-week analyses in **E** and **F**. Significant differences are indicated.

To test the impact of the uORF-edited *PKD1* 5’ UTR on a relatively rapid *Pkd1* missense cystic kidney model, we generated H or ΔH humanized 5’ UTR alleles with the *Pkd1* p.R2216W (orthologue of human *PKD1* p.R2220W) missense variant (38). We call these alleles “*Pkd1^H-RW^*” and “*Pkd1^ΔH-RW^*” (Figure 5A). The *Pkd1^RW^* missense variant expresses reduced mature PC1-CTF protein due to impaired PC1 cleavage, as evidenced by an elevated PC1-FL–to–PC1-CTF ratio. After confirming founders for our humanized 5′ UTR *Pkd1^RW^* alleles and breeding to homozygosity, we tested PC1 expression in *Pkd1*^H/H^, *Pkd1*^H-RW/H-RW^, and *Pkd1*^ΔH-RW/ΔH-RW^ primary cells. As expected, the alleles with the p.R2216W missense variant show reduced PC1-CTF with elevated PC1-FL:PC1-CTF ratio indicating impaired cleavage (Figure 5A). Nonetheless, the allele with ΔuORF1&2 (“ΔH-RW”) achieves cleaved PC1 (PC1-CTF) levels at least equivalent to those from WT *Pkd1* by increasing overall expression (Figure 5A). This illustrates the mechanism by which therapeutic rescue is expected from uORF blocking for missense alleles that are functionally insufficient due to impaired biogenesis. Indeed, our limited assessment of recessive mice (*Pkd1*^H-RW/H-RW^ and *Pkd1*^ΔH-RW/ΔH-RW^) shows dramatic rescue of the cystic kidney phenotype and lifespan from the ΔuORF edit (Supplemental Figure 9). Because of variable phenotype and survival that comes with in utero insufficiency of PC1 dosage, we chose to characterize conditional models of our *Pkd1^RW^* alleles.

### Increased PC1 expression from ΔuORF1&2 edits rescues the Pkd1^RW^ allele.

We induced *Pkd1^H-RW/fl^* or *Pkd1^ΔH-RW/fl^* mice carrying single copy *Pax8^rtTA^;TetO-Cre* alleles from P0–P14 with doxycycline water to their nursing mothers, and sacrificed mice at P14 or P28 (4 weeks). In the first month of life, *Pkd1^H-RW/fl^* mice have progressive cystic kidney disease resulting in large kidneys and markedly reduced renal function, while *Pkd1*^ΔH-RW/fl^ mice have only small cysts or tubule dilation and still preserved kidney size and function (Figure 5, B–F, and Supplemental Figures 10 and 11). At P14 the small cysts are of both proximal and distal nephron origin, whereas the most rapid cyst growth in the *Pkd1^H-RW/fl^* mouse from P14 to P28 is in the distal nephron (Figure 5, B and C). We aged an additional cohort of the mice lacking uORFs (*Pkd1^ΔH-RW/fl^*) to 8 weeks, beyond the lifespan of the fully cystic (*Pkd1^H-RW/fl^*) mice to assess durability of the rescue (Figure 5D and Supplemental Figure 12). KW:BW and cystic index in 8-week *Pkd1^ΔH-RW/fl^* mice suggest mild progression from 4–8 weeks; histology shows this is driven by focal small cysts and — particularly in males — further tubule dilation (Figure 5, D and E). Nonetheless, mean KW:BW and cystic index in 8-week *Pkd1^ΔH-RW/fl^* mice remain closer to the mean values of WT than 4-week *Pkd1^H-RW/fl^* genotype, and these 8-week *Pkd1^ΔH-RW/fl^* mice maintained normal kidney function as assessed by BUN (Figure 5, D–F). The increased severity in males versus females is a feature of all Pax8^rtTA^;TetO-Cre *Pkd1* models at adult time points in our experience not specific to uORF-edited rescue. Overall, these data support 2 key findings: (a) the *Pkd1* p.R2216W missense allele can be functionally rescued by increasing its expression; this rescue is lasting but not complete as slowly growing cysts are present, (b) *PKD1* uORF inhibition can have dramatic therapeutic effect on at least certain cystogenic *PKD1* missense alleles.

## Discussion

**Figure 6:**
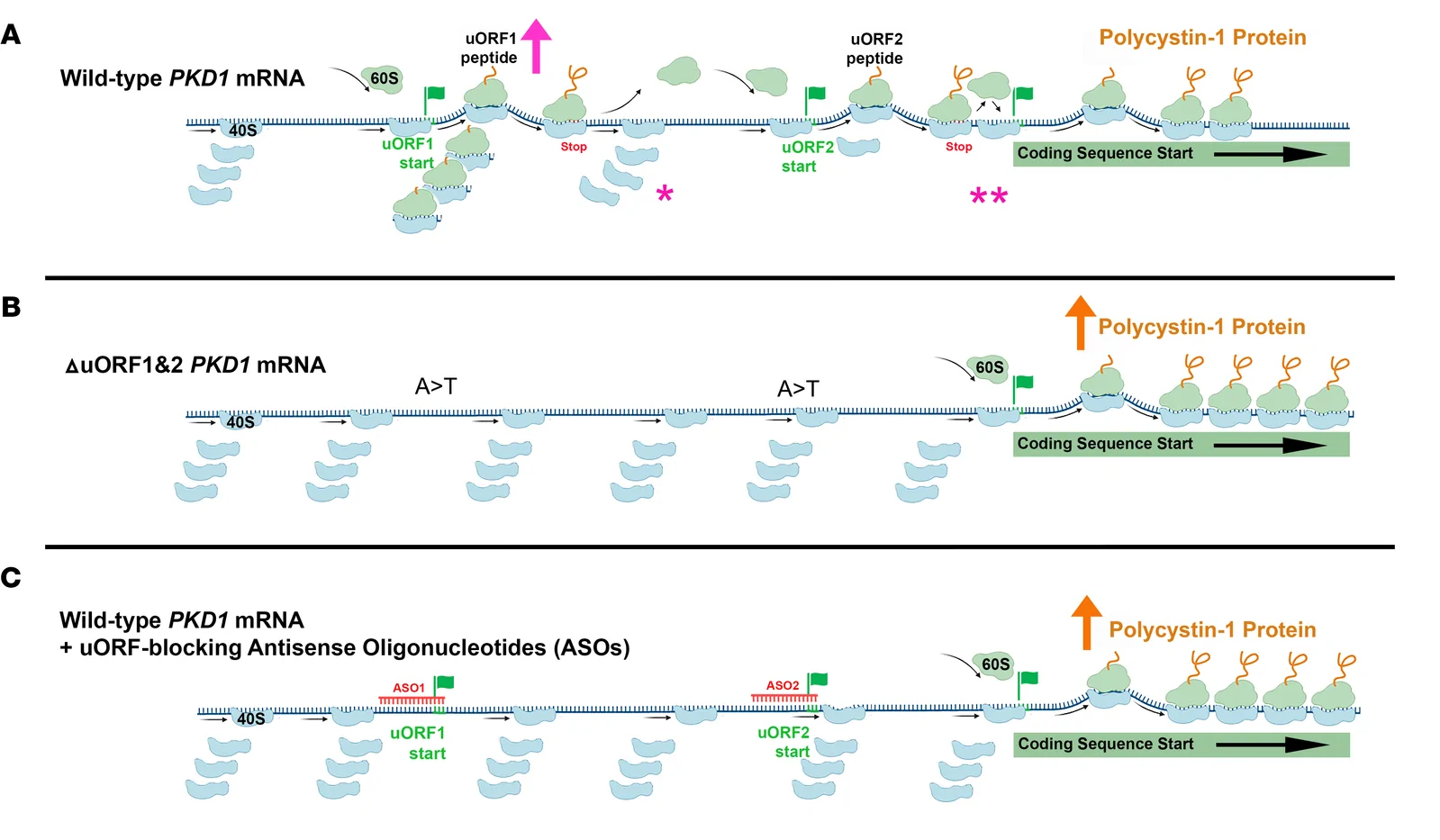
Protein translation from *PKD1* mRNA. Illustration of ribosome scanning, assembly, and reassembly along the 5′ UTR, showing the proposed mechanism by which *PKD1* uORF1 and uORF2 regulate Polycystin-1 protein expression. Scanning of the 40S ribosomal subunit begins at the mRNA 5′ cap and moves in the 5′→3′ direction looking for a start codon (green flag) in the sequence and structural context to initiate translation. When it reaches such a start codon, the 60S ribosomal subunit and other proteins (not shown) composing the translation initiation complex (TIC) assemble and begin protein translation. Upon reaching a stop codon, the TIC with 60S subunit disassembles from the 40s subunit. (**A**) In WT *PKD1* mRNA, our data suggest efficient translation of uORF1 (pink arrow). Given the effect on Polycystin-1 expression, we propose that scanning 40S subunits may often stall or dissociate after uORF1 translation (*) therefore reducing the occurrence of a scanning 40S ribosome getting past this to reach the coding sequence. Our data suggest that *PKD1* uORF2 does not translate as robustly as uORF1, nonetheless its stop codon is only separated from the CDS start by 2 nucleotides, so reassembly (**) of the TIC prior to CDS start may be particularly challenged. (**B**) Genetic edits (2 single nucleotide substitutions) at uORF start codons prevent initiation at uORF1 and uORF2, blocking their translation and thereby increasing ribosome availability for initiation at the downstream Polycystin-1 coding sequence. This study modeled these edits in mouse models and luciferase constructs. (**C**) Sterically blocking the uORF start sites with antisense oligonucleotides (ASOs) as the 40S ribosomal subunit scans across the 5′ UTR similarly impairs translation of uORF1 and uORF2. In both uORF-blocking strategies, loss of uORF translation enhances formation of the initiation complex at the main ORF and increases Polycystin-1 protein synthesis (orange arrow).

We present the identification and validation of translationally active uORFs in the 5′ UTR of the WT *PKD1* gene in humans and other mammalian species. The translation of “uORF1” (*PKD1* c.–87 through –64) and “uORF2” (*PKD1* c.–20 through –3) negatively impacts Polycystin-1 protein translation from available *PKD1* mRNA thereby contributing to the low basal levels of PC1 expression. Genetic edits or steric blocking to prevent the translation of these inhibitory uORFs provide a unique means to increase WT *PKD1* expression at the posttranscriptional level (Figure 6).

*PKD1* uORF-blocking exerts a sequence-specific effect to increase PC1 protein that is independent from other approaches, such as miR-17 inhibition, which increases *PKD1* mRNA, or from folding correctors or chaperones, which aim to increase functional protein. The robust effect in preventing or minimizing cyst burden in our models illustrates the direct relationship between PC1 functional expression level and cystogenic threshold. Our *Pkd1^RW^* model (orthologue of human p.R2220W) has not been studied as extensively as the commonly studied *Pkd1^RC^* (orthologue of human p.R3277C) mouse model; the *Pkd1^RW^* has a more severe defect in PC1 cleavage based on published Western blot data and resultantly has a more severe phenotype in recessive and conditional models, as has been reported in the literature (31, 38). Our data show that the *Pkd1^RW^* allele can be rescued with quantitative increase alone. Importantly, while quantitative increase has a profound benefit for hypomorphic *Pkd1* alleles, mice living with only a single upregulated *Pkd1^RW^* allele (*Pkd1^ΔH-RW/fl^* genotype) do develop mild cystic phenotype that progresses over time, reminding us that the degree of benefit for individual pathogenic missense variants will vary depending on the quantitative versus functional deficit of the allele. The strong efficacy of uORF inhibition in the *Dnajb11* model supports the applicability of this strategy in models of PC1 biogenesis defects, such as the cysts in ADPKD-*DNAJB11* where WT *Pkd1* alleles are present for quantitative increase in expression.

A study published in 2006 implicated that overexpression of WT *PKD1* by 2–15 fold in the form of a BAC transgene caused cysts, lending pause to therapeutic approaches to over express the gene (70). Nonetheless, other models with increased copy number of WT *Pkd1* have not described cysts attributable to PC1 overexpression and suggest that preserving *Pkd1’s* endogenous upstream regulatory sequence is an important consideration (12, 71). The normal phenotype of our *PKD1* uORF–edited models across the lifespan lends further support for the safety of at least modest increase in PC1 expression. Any mutation-agnostic approach that increases protein expression from endogenous alleles in monogenic diseases may increase expression of the pathogenic allele. Fortunately for the safety of this approach in ADPKD, *PKD1* pathogenic alleles are functionally or quantitatively insufficient, without any reported alleles or phenotypes with gain of function. Truncated alleles may undergo nonsense mediated decay to varying extents. Further, the low basal expression levels of PC1 ensure that a 2–4 fold increase still is not a highly expressed protein. This is in stark contrast to the *UMOD* and *MUC1* genes, which are highly expressed in the kidney and for which specific mutations cause ER stress and autosomal dominant tubulointerstitial kidney disease (ADTKD). From experience and additional testing with the humanized alleles, we do not see overt ER stress in the *Pkd1^RW^* genotype with or without humanized UTR nor from its upregulation in the uORF-edited alleles (Supplemental Figure 9D). These factors make *PKD1* a good candidate for safe upregulation.

Characterization of *PKD1* uORFs lends insight into the interpretation of rare human variants in the *PKD1* 5’ UTR. *PKD1* uORFs have recently been independently investigated using luciferase assays by two other groups, and these analyses provide support to our conclusions (72, 73). Each group found an additive effect of editing both uORF1 and uORF2 in luciferase expression. Our study extends the analysis of *PKD1* uORF inhibition to assessment of endogenous Polycystin-1 protein, informs the context of additional upstream uORF translation, and defines successful uORF-blocking ASO sequences. Our ASO studies showed equivalent benefit from single uORF blockade as combination treatment when assessing Polycystin-1 protein itself. One of these published studies implicated what we believe is the first recognized ADPKD disease-causing variant in the *PKD1* 5′ UTR. The patient’s variant, a single nucleotide insertion in uORF1, shifts the reading frame such that uORF1 doesn’t reach a stop codon until after the Polycystin-1 CDS start (73). The pathogenicity of this human variant emphasizes the functional importance of *PKD1* uORFs in humans. Our investigation of alternative *PKD1* 5’ UTR sequences affecting uORFs was not aimed at particular human *PKD1* variants but supports that the termination of uORF1 — initiating at c.–87 and terminating with the stop codon at c.–64 through–66 in WT sequence — prior to CDS start is important to allow sufficient translation at the *PKD1*/Polycystin-1 coding sequence ATG start codon at c.1 (Figure 1H, right-most 2 lanes). Our data illustrate the consequence of *PKD1* uORF1 stop loss — without frameshift — in “Fus1wRenillaATG,” which models the proposed effect from a stop codon readthrough compound or a rare variant to the uORF1 stop codon. This shows protein expression at approximately WT levels of a uORF1-CDS fusion protein, though it remains to be seen whether such an N-terminal extension of 29 amino acids to Polycystin-1 would be functional or would have impaired trafficking and maturation and be pathogenic. Finally, while such an occurrence would be exceedingly rare to occur in a patient with ADPKD, our data suggest that a rare variant abolishing the start codon of uORF1 or uORF2 on an otherwise WT *PKD1* allele would be proposed to raise PC1 dosage and may confer relative protection against PC1 dosage-dependent mechanisms in patients with ADPKD or ADPLD.

Our investigation of *PKD1* uORFs may lend a framework and valuable insights into the understanding and investigation of uORFs for other genes. Our sequential investigation of upstream uORF translation from non-AUG start codons — both CTG (CUG) in this case — illustrates mechanistic principles of uORF regulation, including that (a) the 5’ to 3’ order of translating uORFs is relevant to their consequence on each other’s translational activity, (b) factors beyond Kozak sequence influence translational activity from each uORF, and (c) factors beyond just the location of the uORF stop codon relative to the coding sequence modulate the impact of uORFs on CDS protein expression. Our data, supported by the implicated human pathogenic variant (73), suggest that the most severe effect is when highly expressed uORFs have no in-frame stop codon prior to the coding sequence — although consideration should be given to whether the uORF is in frame with the coding sequence.

In summary, our studies establish a role for human *PKD1* uORFs in the steady-state protein levels of Polycystin-1 in the kidney with a magnitude of effect highly impactful in dosage-dependent models of polycystic kidney disease. We show that steric inhibition of *PKD1* uORFs using an ASO is an effective approach to increase Polycystin-1 protein. We therefore propose that therapeutically targeting *PKD1* uORFs may offer modest to dramatic effects, depending on the germline and somatic variants in the kidney cysts of patients with ADPKD.

## Methods

### Sex as a biological variable.

Our study examined male and female animals. The cystic phenotype was milder in females for some models, thus making rescue less apparent in those cases; nonetheless, similar conclusions are supported for both sexes.

### Reference sequence for human and mammalian orthologue PKD1.

Reference sequence for *PKD1* is defined as the well-established canonical transcript NM_001009944 (Ensembl transcript ENST00000262304.9). Transcripts for other mammalian species were defined per Ensembl.org (74). Further detail on conservation assessment is provided in Supplemental Figure 1.

### Luciferase assay and fusion-protein plasmids.

The dual-luciferase expression plasmid used in this study was generated by ligation of annealed complementary single-stranded oligos (Integrated DNA Technologies) of the desired sequence between NheI and EcoRV restriction sites of a dual-luciferase expression plasmid “psiCHECK-2” (Promega) (75, 76). The insert sequence introduced the human *PKD1* 5′UTR as well as a synonymous change into the 16th and 17th amino acids of the Renilla luciferase to make an AgeI restriction site for subsequent edits. Variations of this plasmid to edit the *PKD1* 5′UTR sequence were then made by similar means by replacement of the shorter fragment between NheI and AgeI sites with a purchased oligo of the desired sequence. ΔuORF3 construct was created by replacing uORF3 CTG start codon with CCG(Pro) using Q5 Site-Directed Mutagenesis Kit (FP: CCGGAGCGGCccgGCCCCGAGCC, RP:GAGCTCGGCCGCCCGCTCG). *PKD1* 5′UTR variations for fusion-protein luciferase constructs were purchased from Synbio Tech and cloned into the shared psiCHECK-2 backbone using standard restriction digest and ligation. Construct sequences were confirmed with Sanger sequencing. Supplemental Table 1 provides sequence information for each genotype tested.

### Cell transfection and luciferase detection.

HEK293 cells were seeded at 6,000 cells per well in 96-well cell culture plates. After overnight incubation, cells were transfected with 50 ng per well of expression plasmid using standard Lipofectamine 3000 (Invitrogen) protocol. Forty-eight hours after transfection, cells were lysed with Passive Lysis Buffer (Promega). Renilla and Fireﬂy luciferase signals were quantified per protocol using Dual-Luciferase Assay System (Promega) read on a Synergy LX multimode reader (BioTek). Results are presented with Renilla luciferase signal normalized to the Fireﬂy luciferase signal from the same well of cells. For parallel assessment of mRNA in luciferase assay studies, additional wells were seeded and transfected in parallel then processed as described below. To obtain sufficient mRNA yield, cells from 3 wells were pooled to generate each biological replicate. All experiments were done in biological triplicate (three independent wells of cells). HEK293 cells were obtained from ATCC (CRL-1573).

### Quantitative reverse transcription PCR.

For all experiments with mRNA quantification, RNA was extracted from independent wells or plates of cells plated and treated in parallel with the equivalent sample for protein assessment. Methods for protein extraction make it not possible to extract RNA and protein from the same well. Total RNA extraction was performed using the RNeasy Plus Mini Kit (Qiagen 74134). cDNA synthesis was prepared using the iScript cDNA synthesis kit (BIO-RAD 1708891). Quantitative PCR (qPCR) was performed using standard protocol with iQ SYBR Green Supermix (BIO-RAD 1708882), technical triplicates for each biological sample, and normalization to 18s ribosomal RNA or *GAPDH*. Primer sequences are shown in Supplemental Table 3.

### Cell culture and primary cell isolation.

All immortalized cells used for *PKD1*/PC1 assessment were grown in DMEM/F12 in 1:1 combination with 1mg/mL Penicillin/Streptomycin and 10% FBS concentration and serum starved prior to collection of cell lysate or mRNA from confluent plate/well. Mouse *Pkd1-V5* wild-type and missense cell lines are serum starved for 3 days in media containing 0.5% FBS. RPE cells are serum starved for 3 days in media containing 0.1% FBS. Serum starvation is a standard practice for cell synchronization and primary cilium generation used prior to assessment of ciliary proteins in epithelial cells. Primary cells were isolated from mice at P9 and dissociated with a GentleMACS Octo Dissociator and plated in primary tubular epithelial cell media (DMEM with 1% Pen/Strep, 1% FBS, 0.1% ITS, 0.1% EGF and 1.3 ng/ml T3).

### Generation of PKD1 knockout cell line in RPE cells.

A preparation of 1000 cells/uL of RPE cells was nucleofected with ribonucleoprotein (RNP) containing CRISPR/Cas9 components per standard protocol using 4D Nucleofector (Lonza). The RNP included Cas9 protein with transfer RNA including the gRNA sequence targeting *PKD1* exon 15 as published by others (77). The resultant cell population was clonally diluted. The selected clone was confirmed by western blot and Sanger sequencing to have truncating mutation in exon 15 (*PKD1*:c.4013insC:p.1338fs). RPE cells were obtained from ATCC (CRL-4000).

### Antisense oligonucleotides.

Antisense oligonucleotides (ASOs) were designed by the authors as described and purchased from Integrated DNA technologies (IDT). 100 μM ASOs were applied to cell culture media to produce the indicated concentrations for passive uptake. Based on optimization studies (data not shown), a concentration of 20nM was used for each ASO in the data provided. For ASO treatment of cells transfected with luciferase plasmid, the ASO was added to the fresh culture media at the time of transfection, luciferase activity and Renilla/Firefly proteins were tested 48 hours after transfection. For ASO assessment in primary cells, ASO was added to fresh culture media 48 hours after harvest, and serum starvation begun the following day based on desired cell confluence. For RPE and *Pkd1-V5* cells, ASO was added to fresh culture media at the time of starting serum starvation.

### Immunoblotting.

Immunoblotting was performed on whole cell lysate prepared using RIPA buffer (10 mM Tris-Cl pH 8.0, 1 mM EDTA, 0.5 mM EGT A, 1% Triton X-100, 0.1% sodium deoxycholate, 0.1% SDS, 140 mM NaCl, with cOmplete Mini, EDTA free protease inhibitor cocktail tablet [Roche 11836170001] per 10ml RIPA), combined with 5X Sample buffer (National Diagnostics). Protein lysate was run on Novex 3–8% Tris Acetate gel and transferred to nitrocellulose membrane (0.45 um) using 24V at room temp overnight. For blots involving the small Renilla Luciferase protein, a Biorad 4–20% Tris-Glycine gel was used and transfer accomplished using 80V at room temperature for 1 hour. For Ponceau staining, the membrane was removed from the transfer cassette, washed in water, submerged in Ponceau S Solution (Sigma) for 10 minutes, then washed briefly in water before being allowed to dry on tissue. After rehydration in methanol and wash in TBST, membranes were blocked with 5% non-fat milk in TBST for 1 hour. The following primary antibodies were used in 5% milk in TBST: anti-V5 (mouse species used for western blots on cell lysates, 46-0705, Invitrogen; 1:2000), anti-V5 (rabbit species used for western blots on tissue lysate, D3H8Q #13202, Cell Signaling Technology; 1:1000), anti–PC1-LRR (7e12, sc-130554, Santa Cruz Biotechnology; 1:2000), anti-vinculin (7F9, sc-73614, Santa Cruz Biotechnology, 1:10000), anti-Hsp90 (4877, Cell Signaling Technology, 1:10000), anti-BiP (c5012 #3117, Cell Signaling Technology, 1:1000), anti-Renilla (MA5-32013, Invitrogen, 1:5000) and anti-Firefly (MA1-16880, Invitrogen, 1:10000). Secondary antibodies were obtained from Jackson Immuno Research (RRID: AB_2307391, AB_2338511) and used at 1:10,000 dilution. Super Signal West Femto Maximum Sensitivity Substrate (34096, Thermo Scientific) was used as a substrate for HRP for PC1 detection, and Pierce ECL Western Blotting Substrate (32209, Thermo Scientific) for other proteins.

### Mouse lines and generation of humanized PKD1 5′UTR mouse alleles.

All experiments were conducted in accordance with Yale University Institutional Animal Care and Use Committee guidelines and procedures. The following strains of mice were used in this study: *Pkd1-V5* and *Pkd1-p.R2216W-V5* on a mixed C57BL/6J and 129/Sv background (38), *Dnajb11^flox^* on C57BL/6N (69), and Ksp-Cre (Jax Strain #012237), *Pkd1^null^* (78), *Pkd1^flox^* (79), Pax8^rtTA^ (JAX Strain #007176), TetO-Cre (Jax Strain # 006234) which are > 90% C57BL/6J. Immortalized cells lines from mice carrying the V5-tagged alleles are previously described (38). Breeding strategies ensured the closest possible genetic background between genotype comparisons (litter mates in the *Dnajb11* model and shared male breeders with closely related *Pkd1^ΔH or H-RW/fl^* mothers). We targeted the 5’UTR of *Pkd1-V5* and *Pkd1-p.R2216W-V5* mice by injection of CRISPR/Cas9 components targeting the left and right end of the 313-nucleotide mouse *Pkd1* 5’UTR (guides sequences CCGCCCCTGCGCTTCCAAGT (reverse strand) and GGGGAACCGGGGCCATGCGC (forward strand), respectively) and circularized DNA repair template into 0.5day embryos by the Yale Genome Editing Center. The repair template consisted of either the wild-type 209-nucleotide human *PKD1* 5’UTR sequence “H” or the that with two A>T edits at the c.-20 and c.-87 position to edit uORF1 and uORF2 start codons “ΔH” flanked by 100 nucleotides of the mouse sequence that precedes and follows the removed mouse 5’UTR. Founder mice with the precise replacement of only the mouse 313-nucleotide *Pkd1* 5′UTR with the 209-nucleotide human *PKD1* 5′UTR were produced for 3 of the 4 intended alleles confirmed with internal and external PCR and Sanger sequencing, but by chance there were no “H-RW” founders so a subsequent round of CRISPR introduced the p.R2216W edit to the H allele using the established approach (38). Founder and F1 alleles were confirmed by internal, external, and internal-external PCR band size and Sanger sequencing.

### Mouse sacrifice, tissue perfusion, histology, and Immunofluorescence.

The sacrifice and collection of mouse specimens was completed as previously described (69). Briefly, blood was obtained at the time of sacrifice by cardiac puncture and centrifuged for serum separation. All tissues were perfused with 1x PBS prior to harvest. Kidneys collected for histology or immunofluorescence were subsequently perfused with 4% paraformaldehyde (PFA) then stored in PFA before paraffin embedding (for histology sections) or tissueTek (Sakura product 4583) embedding on liquid nitrogen for subsequent cryotome sectioning for immunofluorescence studies.

### Histology scans and cystic index calculation.

Whole-kidney scans were obtained using the scan slide module in MetaMorph (Universal Imaging). Cystic Index was calculated as previously described (69).

### Statistics.

Data were analyzed by one-way ANOVA for the comparison of means of multiple samples followed by multiple comparison testing for pairwise statistics. An unpaired 2-tailed *t*-test (parametric) was used to compare the means of 2 samples. *P* ≤ 0.05 was considered significant. *P*-values reaching significance are represented on data plots. Mean and SEM are represented.

### Study approval.

This research was conducted in full compliance with animal protocol #2023-20539 approved by the Institutional Animal Care and Use Committee (IACUC) of Yale University, New Haven, Connecticut, USA.

### Data availability.

Numerical data values visualized in plots are provided in a supplemental Supporting Data Values file. All data will be made available upon publication. Animal models are subject to the agreements in place by the funding agency. The original data generated from this study are fully presented in this manuscript.

## Author contributions

ZL, ZG, and SC designed research studies, conducted experiments, acquired data, analyzed data, and contributed to writing and editing the manuscript. ZL and ZG contributed equally, but ZL contributed to initial project design so is listed first. RB, KD, and SF provided reagents, WB conceptualized the project, designed research studies, analyzed data, and wrote the manuscript.

## Conflict of interest

WB and ZL are co-inventors for a patent submitted by Yale University (US-2025339561-A1; AU-2023275606-A1, CA-3255304-A1, CN-119546758-A, EP-4532709-A1, and JP-2025519109).

## Funding support

This work is the result of NIH funding, in whole or in part, and is subject to the NIH Public Access Policy. Through acceptance of this federal funding, the NIH has been given a right to make the work publicly available in PubMed Central.

- NIH/National Institute of Diabetes and Digestive and Kidney Diseases (K08DK119642, R03DK134793, R01DK138015).
- The Doris Duke Charitable Foundation (Clinical Scientist Development Award #2021195 to WB).
- The Polycystic Kidney Disease Foundation (Fellowship #946986 to ZL).

## Supplementary Material

Supplemental data

Unedited blot and gel images

Supporting data values

## Version 1 07/28/2026

In-Press Preview

## Version 2 09/15/2026

Electronic publication

## References

[1] Torres VE (2007). Autosomal dominant polycystic kidney disease. Lancet.

[2] Harris PC, Torres VE (2009). Polycystic kidney disease. Annu Rev Med.

[3] Chebib FT, Torres VE (2016). Autosomal dominant polycystic kidney disease: core curriculum 2016. Am J Kidney Dis.

[4] Van Keimpema L (2011). Patients with isolated polycystic liver disease referred to liver centres: clinical characterization of 137 cases. Liver Int.

[5] Barten TRM (2023). Higher need for polycystic liver disease therapy in female patients: sex-specific association between liver volume and need for therapy. Hepatology.

[6] Schonauer R (2023). Sex, genotype, and liver volume progression as risk of hospitalization determinants in autosomal dominant polycystic liver disease. Gastroenterology.

[7] Qian Q (2003). Clinical profile of autosomal dominant polycystic liver disease. Hepatology.

[8] Sierks D (2022). Modelling polycystic liver disease progression using age-adjusted liver volumes and targeted mutational analysis. JHEP Rep.

[9] Torres VE (2017). Tolvaptan in later-stage autosomal dominant polycystic kidney disease. N Engl J Med.

[10] Torres VE (2012). Tolvaptan in patients with autosomal dominant polycystic kidney disease. N Engl J Med.

[11] Chebib FT, Perrone RD (2023). Drug development in autosomal dominant polycystic kidney disease: opportunities and challenges. Adv Kidney Dis Health.

[12] Fedeles SV (2011). A genetic interaction network of five genes for human polycystic kidney and liver diseases defines polycystin-1 as the central determinant of cyst formation. Nat Genet.

[13] Cornec-Le Gall E (2018). Genetic complexity of autosomal dominant polycystic kidney and liver diseases. J Am Soc Nephrol.

[14] Li A (2003). Mutations in PRKCSH cause isolated autosomal dominant polycystic liver disease. Am J Hum Genet.

[15] Drenth JP (2003). Germline mutations in PRKCSH are associated with autosomal dominant polycystic liver disease. Nat Genet.

[16] Davila S (2004). Mutations in SEC63 cause autosomal dominant polycystic liver disease. Nat Genet.

[17] Porath B (2016). Mutations in GANAB, encoding the glucosidase IIα subunit, cause autosomal-dominant polycystic kidney and liver disease. Am J Hum Genet.

[18] Besse W (2017). Isolated polycystic liver disease genes define effectors of polycystin-1 function. J Clin Invest.

[19] Cornec-Le Gall E (2018). Monoallelic mutations to DNAJB11 cause atypical autosomal-dominant polycystic kidney disease. Am J Hum Genet.

[20] Besse W (2019). *ALG9* mutation carriers develop kidney and liver cysts. J Am Soc Nephrol.

[21] Senum SR (2022). Monoallelic IFT140 pathogenic variants are an important cause of the autosomal dominant polycystic kidney-spectrum phenotype. Am J Hum Genet.

[22] Lemoine H (2022). Monoallelic pathogenic ALG5 variants cause atypical polycystic kidney disease and interstitial fibrosis. Am J Hum Genet.

[23] Qian F (1996). The molecular basis of focal cyst formation in human autosomal dominant polycystic kidney disease type I. Cell.

[24] Watnick TJ (1998). Somatic mutation in individual liver cysts supports a two-hit model of cystogenesis in autosomal dominant polycystic kidney disease. Mol Cell.

[25] Wu G, Somlo S (2000). Molecular genetics and mechanism of autosomal dominant polycystic kidney disease. Mol Genet Metab.

[26] Janssen MJ (2011). Secondary, somatic mutations might promote cyst formation in patients with autosomal dominant polycystic liver disease. Gastroenterology.

[27] Tan AY (2018). Somatic mutations in renal cyst epithelium in autosomal dominant polycystic kidney disease. J Am Soc Nephrol.

[28] Zhang Z (2021). Detection of PKD1 and PKD2 somatic variants in autosomal dominant polycystic kidney cyst epithelial cells by whole-genome sequencing. J Am Soc Nephrol.

[29] Mallawaarachchi AC (2024). Somatic mutation in autosomal dominant polycystic kidney disease revealed by deep sequencing human kidney cysts. NPJ Genom Med.

[30] Rossetti S (2009). Incompletely penetrant PKD1 alleles suggest a role for gene dosage in cyst initiation in polycystic kidney disease. Kidney Int.

[31] Hopp K (2012). Functional polycystin-1 dosage governs autosomal dominant polycystic kidney disease severity. J Clin Invest.

[32] Antignac C (2015). The future of polycystic kidney disease research--as seen by the 12 Kaplan awardees. J Am Soc Nephrol.

[33] Cornec-Le Gall E (2019). Autosomal dominant polycystic kidney disease. Lancet.

[34] Heyer CM (2016). Predicted mutation strength of nontruncating PKD1 mutations aids genotype-phenotype correlations in autosomal dominant polycystic kidney disease. J Am Soc Nephrol.

[35] Cornec-Le Gall E (2013). Type of PKD1 mutation influences renal outcome in ADPKD. J Am Soc Nephrol.

[36] Lakhia R (2022). PKD1 and PKD2 mRNA cis-inhibition drives polycystic kidney disease progression. Nat Commun.

[37] https://medcommshydhosting.com/CRM/Renal/ASN/presentations/2025/oral-presentation/ASN2025_Farabursen_increases_urinary_Oral_abstract.pdf.

[38] Krappitz M (2023). XBP1 activation reduces severity of polycystic kidney disease due to a nontruncating polycystin-1 mutation in mice. J Am Soc Nephrol.

[39] Fedeles SV (2015). Sec63 and Xbp1 regulate IRE1α activity and polycystic disease severity. J Clin Invest.

[40] Dong K (2021). Renal plasticity revealed through reversal of polycystic kidney disease in mice. Nat Genet.

[41] Ward CJ (1996). Polycystin, the polycystic kidney disease 1 protein, is expressed by epithelial cells in fetal, adult, and polycystic kidney. Proc Natl Acad Sci U S A.

[42] Geng L (1997). Distribution and developmentally regulated expression of murine polycystin. Am J Physiol.

[43] Hu J, Harris PC (2020). Regulation of polycystin expression, maturation and trafficking. Cell Signal.

[44] Wieder N (2024). Differences in 5′untranslated regions highlight the importance of translational regulation of dosage sensitive genes. Genome Biol.

[45] Leppek K (2018). Functional 5′ UTR mRNA structures in eukaryotic translation regulation and how to find them. Nat Rev Mol Cell Biol.

[46] Johnstone TG (2016). Upstream ORFs are prevalent translational repressors in vertebrates. EMBO J.

[47] Brar GA, Weissman JS (2015). Ribosome profiling reveals the what, when, where and how of protein synthesis. Nat Rev Mol Cell Biol.

[48] Jain N (2023). Small open reading frames: a comparative genetics approach to validation. BMC Genomics.

[49] Li F (2024). A peptide encoded by upstream open reading frame of *MYC* binds to tropomyosin receptor kinase B and promotes glioblastoma growth in mice. Sci Transl Med.

[50] Huang N (2021). An Upstream open reading frame in phosphatase and tensin homolog encodes a circuit breaker of lactate metabolism. Cell Metab.

[51] Jayaram DR (2021). Unraveling the hidden role of a uORF-encoded peptide as a kinase inhibitor of PKCs. Proc Natl Acad Sci U S A.

[53] Sasaki S (2019). Steric inhibition of 5’ UTR regulatory elements results in upregulation of human CFTR. Mol Ther.

[54] Kidwell A (2023). Translation rescue by targeting Ppp1r15a through its upstream open reading frame in sepsis-induced acute kidney injury in a murine model. J Am Soc Nephrol.

[55] Hedaya OM (2023). Secondary structures that regulate mRNA translation provide insights for ASO-mediated modulation of cardiac hypertrophy. Nat Commun.

[56] McGillivray P (2018). A comprehensive catalog of predicted functional upstream open reading frames in humans. Nucleic Acids Res.

[57] Hernandez G (2024). Functional analysis of the AUG initiator codon context reveals novel conserved sequences that disfavor mRNA translation in eukaryotes. Nucleic Acids Res.

[58] Kerpedjiev P (2015). Forna (force-directed RNA): simple and effective online RNA secondary structure diagrams. Bioinformatics.

[59] Hofacker IL (2003). Vienna RNA secondary structure server. Nucleic Acids Res.

[60] http://rna.tbi.univie.ac.at/forna.

[61] http://rna.tbi.univie.ac.at/cgi-bin/RNAWebSuite/RNAfold.cgi.

[62] Kiniry SJ (2018). The GWIPS-viz Browser. Curr Protoc Bioinformatics.

[63] https://gwips.ucc.ie.

[64] http://rnainformatics.org.cn/RiboUORF.

[65] Liu Q (2023). Ribo-uORF: a comprehensive data resource of upstream open reading frames (uORFs) based on ribosome profiling. Nucleic Acids Res.

[66] Liang XH (2017). Antisense oligonucleotides targeting translation inhibitory elements in 5′ UTRs can selectively increase protein levels. Nucleic Acids Res.

[67] Liang XH (2016). Translation efficiency of mRNAs is increased by antisense oligonucleotides targeting upstream open reading frames. Nat Biotechnol.

[68] Roberts TC (2020). Advances in oligonucleotide drug delivery. Nat Rev Drug Discov.

[69] Ghosh Roy S (2023). Dnajb11-kidney disease develops from reduced polycystin-1 dosage but not unfolded protein response in mice. J Am Soc Nephrol.

[70] Thivierge C (2006). Overexpression of PKD1 causes polycystic kidney disease. Mol Cell Biol.

[71] Kurbegovic A (2023). Modeling *Pkd1* gene-targeted strategies for correction of polycystic kidney disease. Mol Ther Methods Clin Dev.

[72] Chen L (2025). Translational regulation of PKD1 by evolutionarily conserved upstream open reading frames. RNA Biol.

[73] Wedd L (2025). PKD1 5’UTR variants are a rare cause of disease in ADPKD and suggest a new focus for therapeutic development. Eur J Hum Genet.

[74] Martin FJ (2023). Ensembl 2023. Nucleic Acids Res.

[75] Calvo SE (2009). Upstream open reading frames cause widespread reduction of protein expression and are polymorphic among humans. Proc Natl Acad Sci U S A.

[76] Vasu K (2023). Translational control of murine adiponectin expression by an upstream open reading frame element. RNA Biol.

[77] Kuraoka S (2020). *PKD1*-dependent renal cystogenesis in human induced pluripotent stem cell-derived ureteric bud/collecting duct organoids. J Am Soc Nephrol.

[78] Wu G (2002). Trans-heterozygous Pkd1 and Pkd2 mutations modify expression of polycystic kidney disease. Hum Mol Genet.

[79] Shibazaki S (2008). Cyst formation and activation of the extracellular regulated kinase pathway after kidney specific inactivation of Pkd1. Hum Mol Genet.

