# Supplementary material for: PKD1 upstream open reading frames affect Polycystin-1 expression and polycystic kidney disease phenotypes
Source: J Clin Invest. 2026 Jul 28;136(18):e203177. doi: 10.1172/JCI203177 (PMC13574166; doi:10.1172/JCI203177)
Supplement: Supplementary file 1 [file jci-136-203177-s279.pdf]

A B

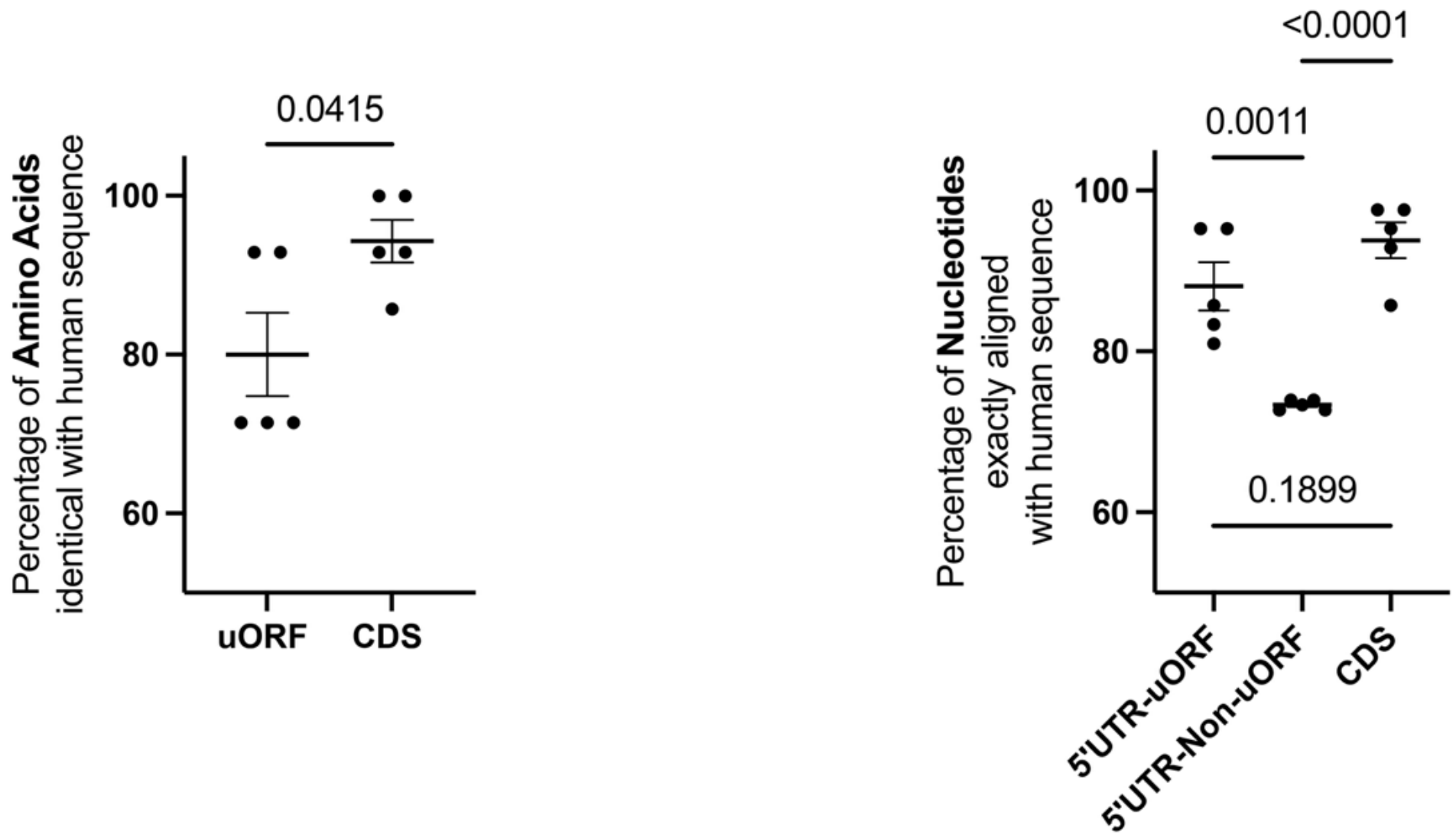

	***Pkd1* uORF amino acids**	***Pkd1* CDS amino acids**
cat	13/14 (93%)	14/14 (100%)
pig	10/14 (71%)	14/14 (100%)
mouse	10/14 (71%)	12/14 (86%)
goat	10/14 (71%)	12/14 (86%)
horse	13/14 (93%)	13/14 (93%)

compared to human

	***Pkd1* 5'UTR uORF nucleotides**	***Pkd1* 5'UTR non-uORF nucleotides**	***Pkd1* CDS nucleotides (first 42)**
cat	40/42 (95%)	120/165 (73%)	41/42 (98%)
pig	34/42 (81%)	120/165 (73%)	41/42 (98%)
mouse	36/42 (86%)	122/165 (74%)	36/42 (86%)
goat	35/42 (83%)	121/165 (73%)	39/42 (93%)
horse	40/42 (95%)	122/165 (74%)	40/42 (95%)

compared to aligned human *PKD1* mRNA sequence

Supplemental Figure 1: Conservation of *PKD1* uORFs across mammalian species. (A) The mammalian species' amino acid sequence for the 14 amino acids of combined uORF1 + uORF2 including stop codons, and separately for the first 14 amino acids of the *PKD1* coding sequence are compared to the corresponding human sequence. (B) The mammalian species' nucleotide sequence for the 42 nucleotides of combined uORF1 + uORF2 including stop codons ("uORF nucleotides"), and separately for the 165 remaining nucleotides in the 5'UTR starting from the first nucleotide that aligns with the start of human *PKD1* 5'UTR and excluding the 2 nucleotides between uORF2 and CDS ("non-uORF nucleotides"), and separately for the first 42 nucleotides of the CDS, were compared to the corresponding human sequence. The 5'UTR for each species is defined to be the Ensembl canonical transcript and the species represented are chosen based on availability of this defined 5'UTR annotation. NCBI Align Blast "somewhat similar sequences" (blastn) defined the alignment used for these calculations. Bases in a species' transcript preceding the sequence aligning with start of human *PKD1* 5'UTR were excluded from analysis. SNVs or indels were included as differences. The table represent the number of identical nucleotides. Two-tailed t test was applied in panel A, one-way ANOVA was applied in panel B. Significant differences are indicated.

Supplemental Figure 2

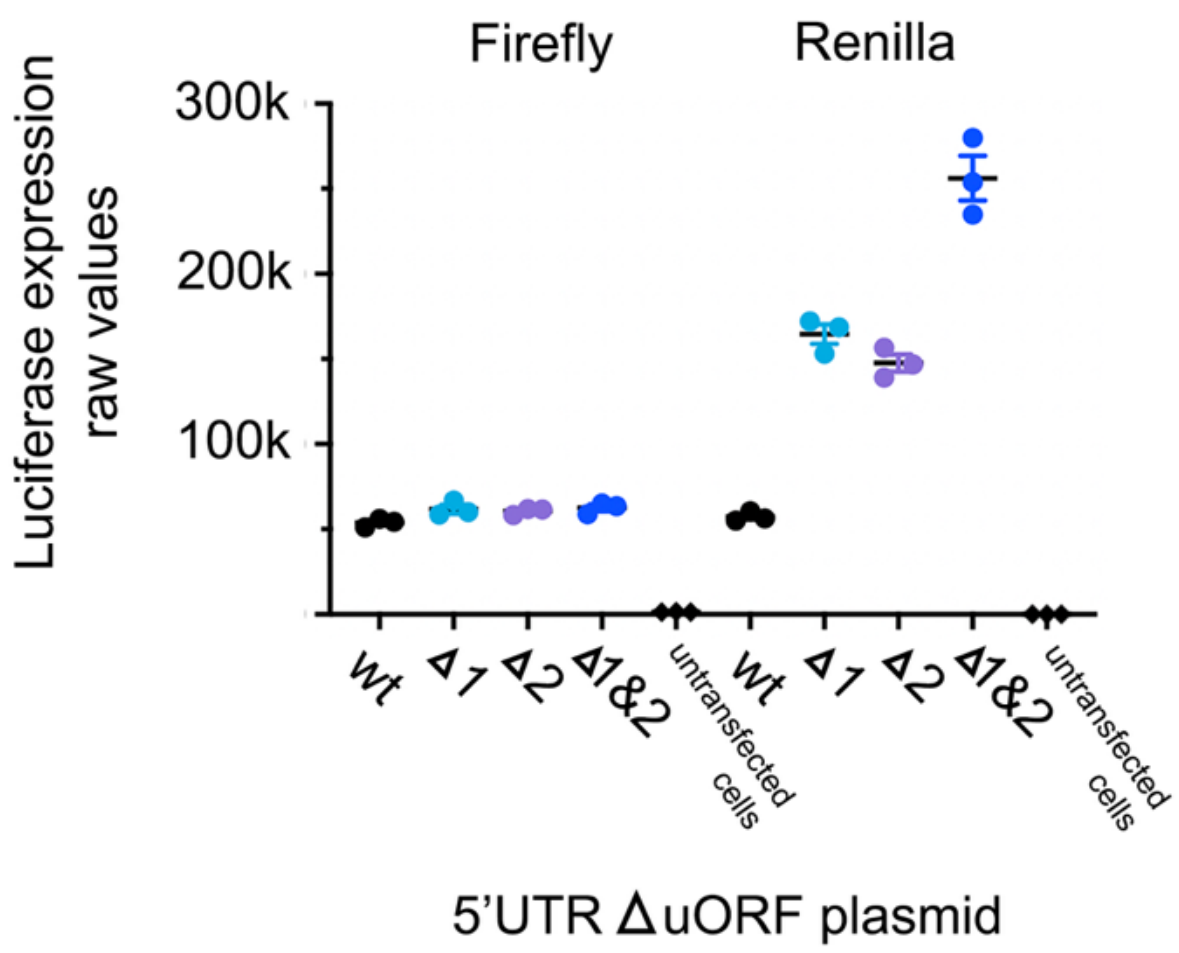

Supplemental Figure 3

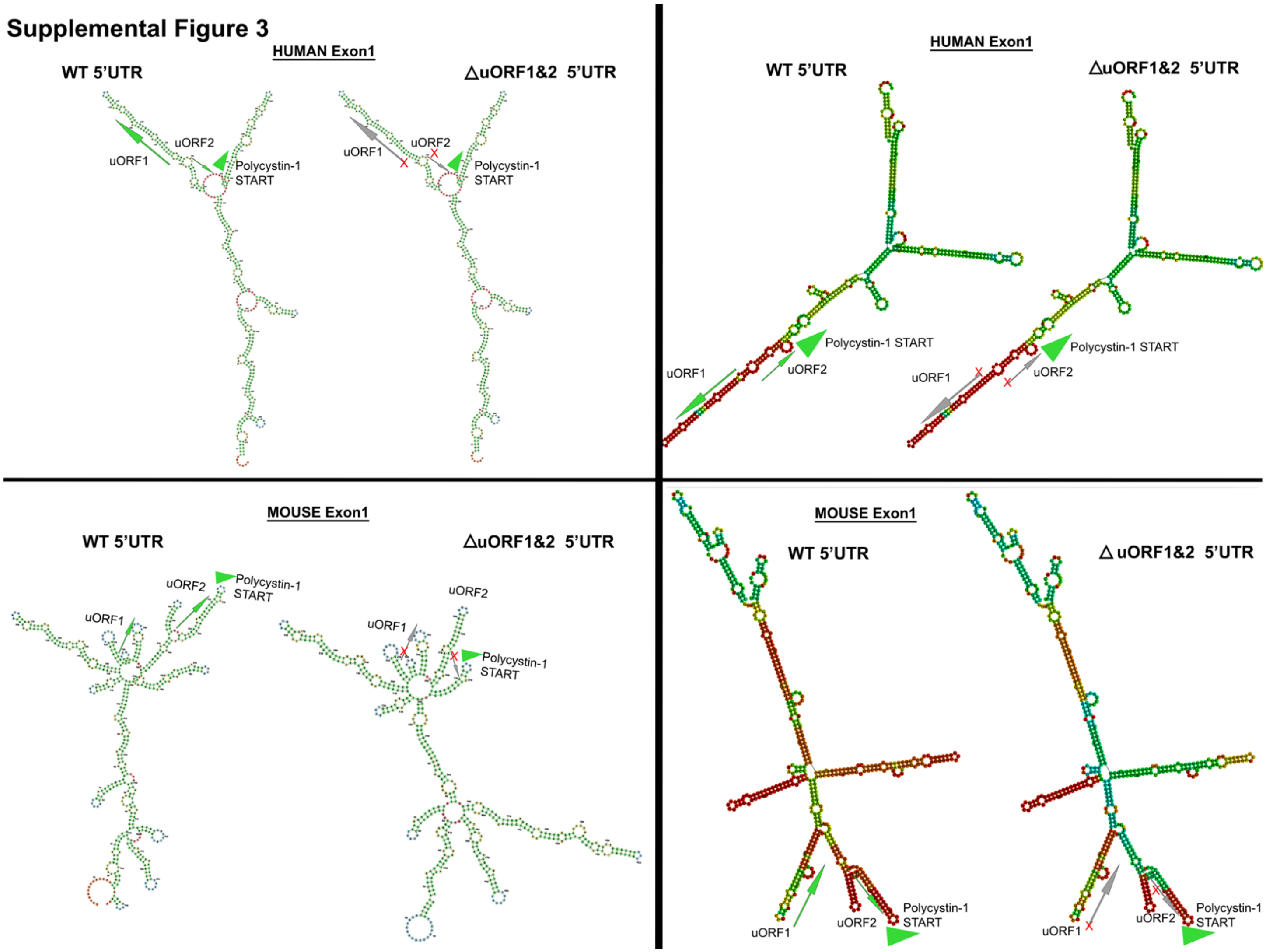

Supplemental Figure 4

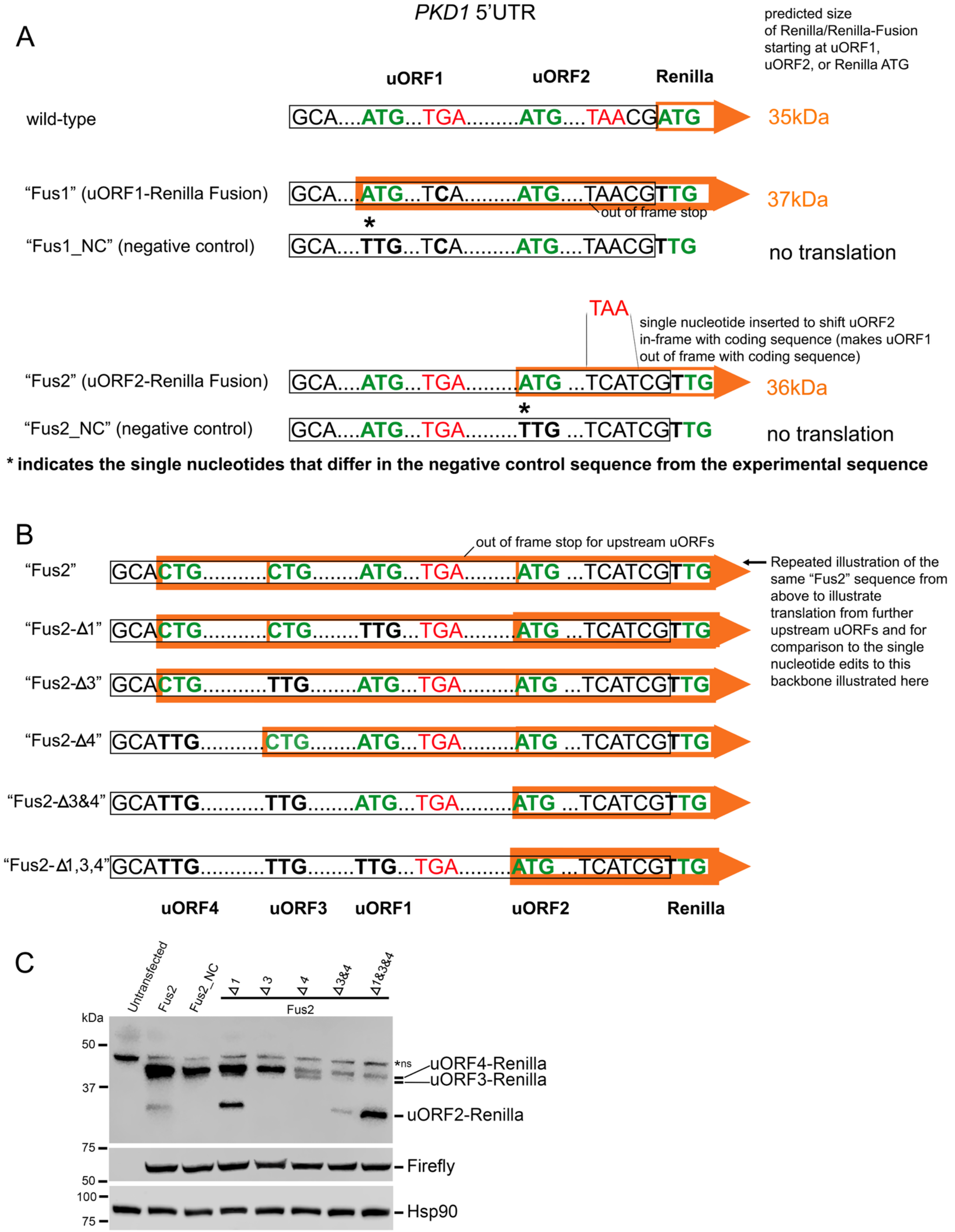

Supp Figure 5- Publicly available Riboseq data

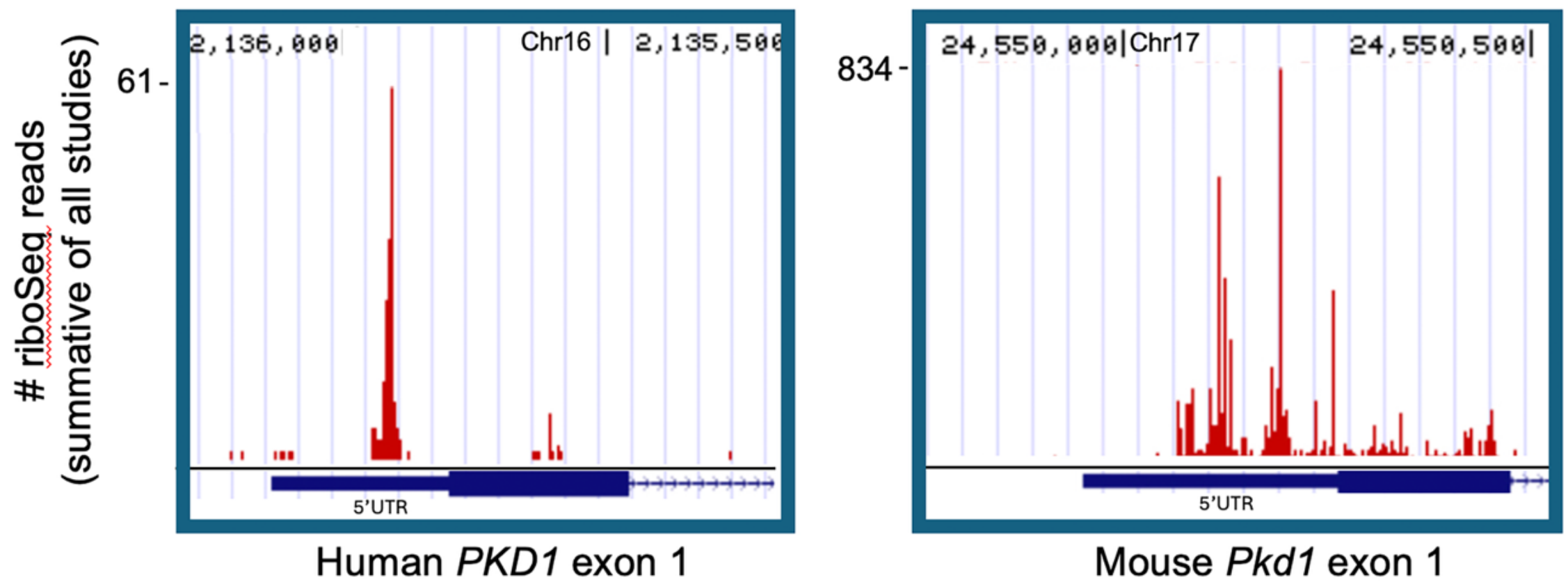

GWIPS-vis Genome Browser, accessed Oct 2024

Supplemental Figure 6

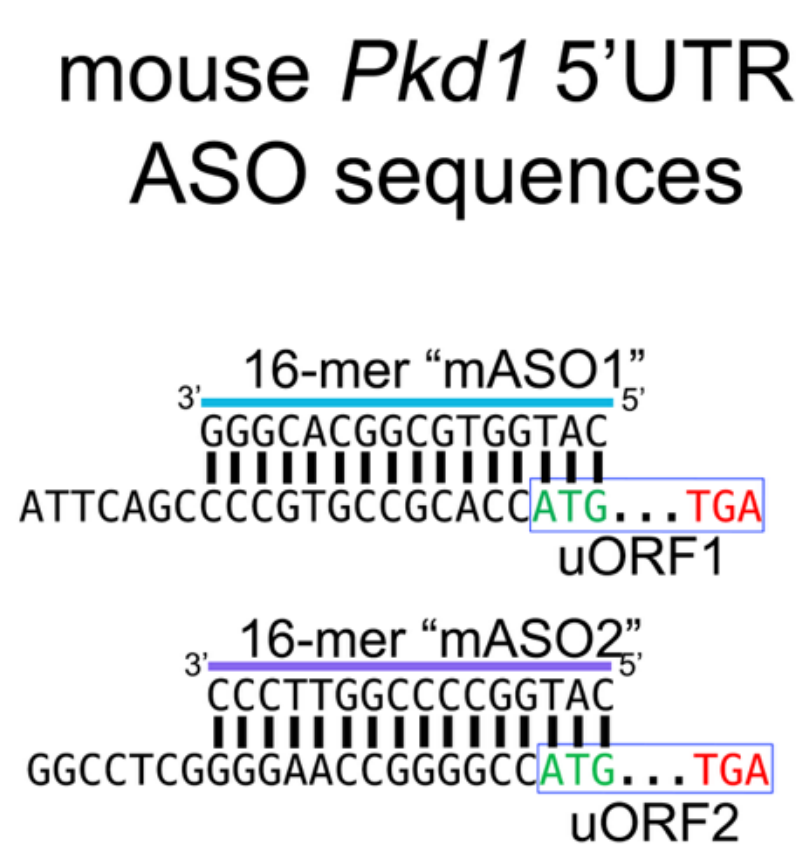

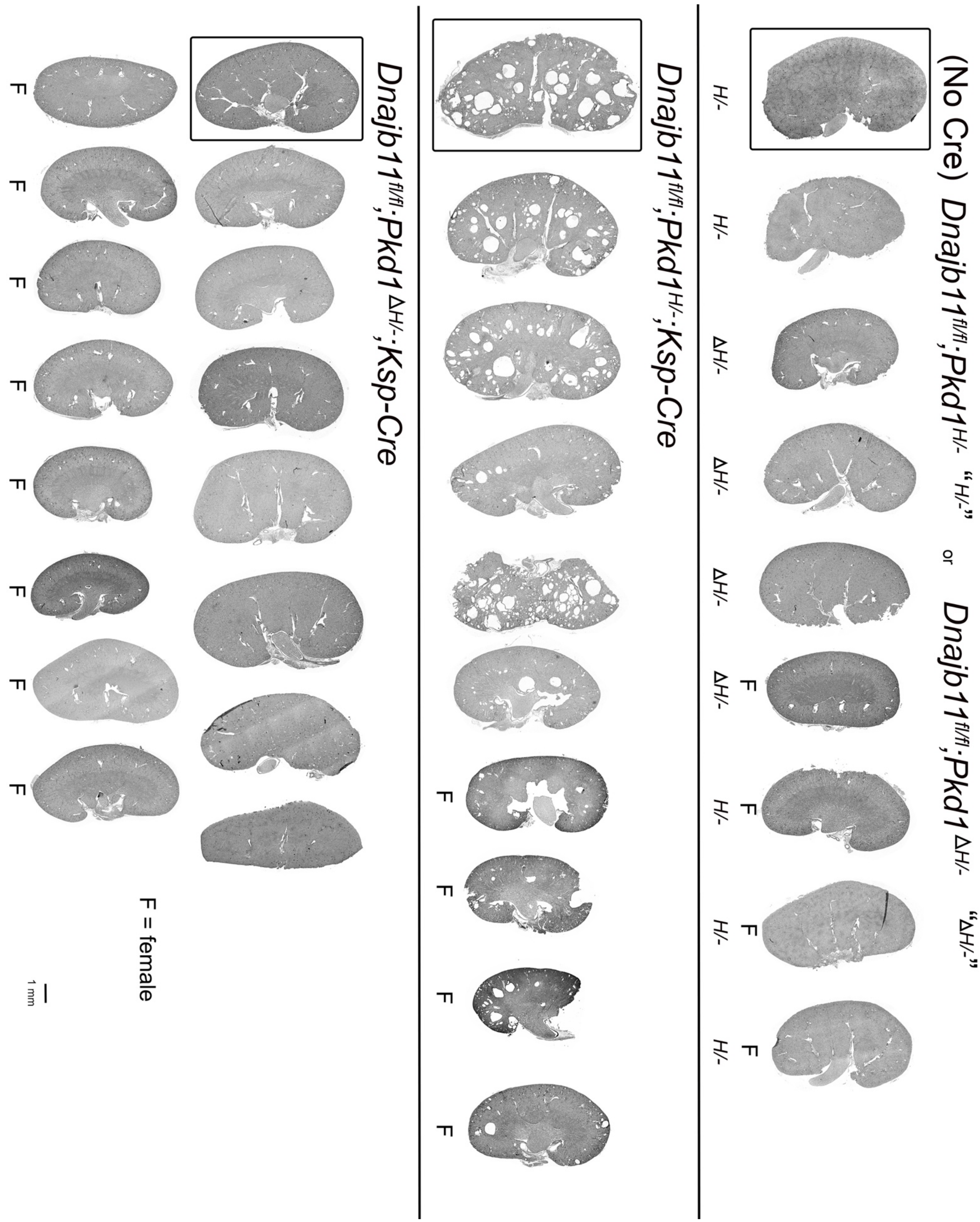

Supplemental Figure 7: Kidney scans for all mice in the *Dnajb11* model analysis, sacrificed at 9 weeks of age. Kidneys from the indicated genotypes are shown. Mice for this model were produced by crossing *Dnajb11*$^{fl/fl}$;*Pkd1*$^{\Delta H/H}$ mice with *Dnajb11*$^{fl/fl}$ mice carrying the *Pkd1*$^{-}$ allele with *Ksp-Cre* present on one breeder parent only. This strategy allowed for a cohort composed of littermates for the experimental comparison. The kidney images in the boxes are also shown in Figure 4F.

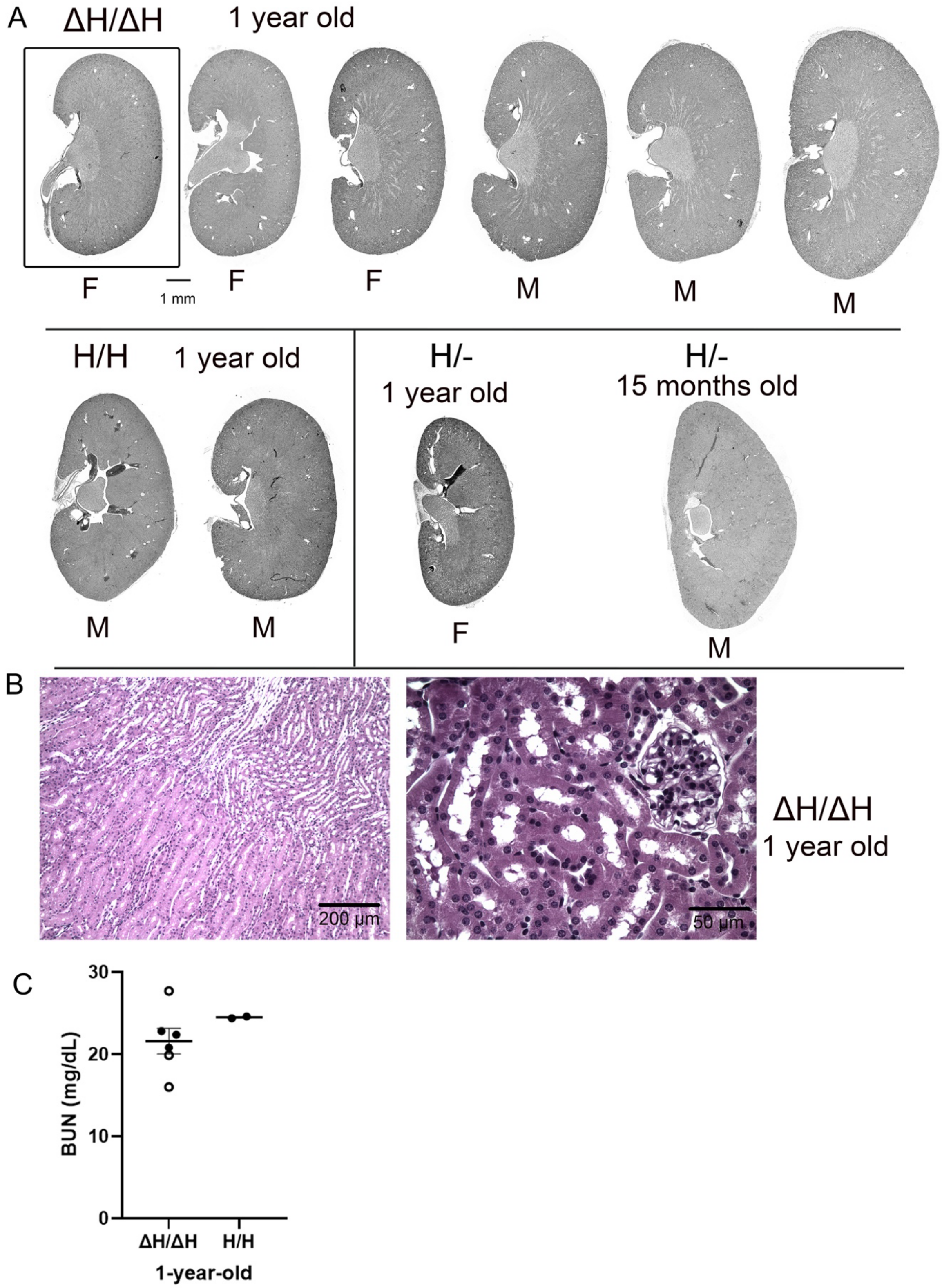

Supplemental Figure 8: Mice with humanized 5'UTR aged to 1 year. *Pkd1*$^{\Delta H/\Delta H}$ ("ΔH/ΔH") mice which lack uORF peptide expression have normal kidneys throughout life. Humanization of *Pkd1* 5'UTR between unedited mouse upstream regulatory sequences and coding sequence is tolerated without apparent effect. (A) Kidney scans, (B) representative kidney histology, and (C) kidney function represented by blood urea nitrogen (BUN) in *Pkd1*$^{\Delta H/\Delta H}$ ("ΔH/ΔH") and *Pkd1*$^{H/H}$ ("H/H") mice aged to 1 year. Serum was not available for H/- mice at this aged timepoint. The kidney image in the box is also represented in Figure 4I. Two-tailed t test was applied in panel C but no significant difference was present. .

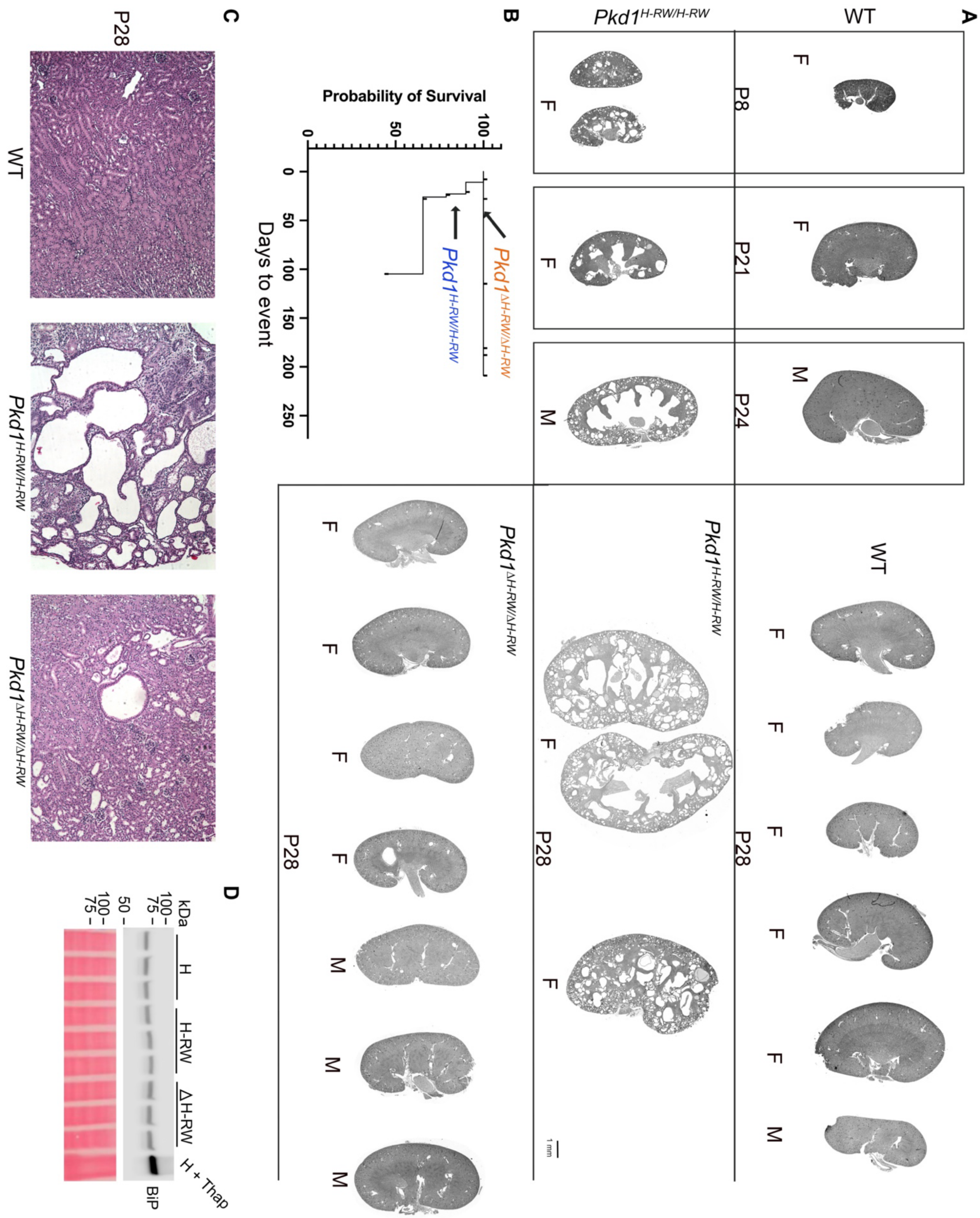

Supplemental Figure 9: Limited assessment of *Pkd1*$^{H\text{-}RW/H\text{-}RW}$ recessive model with early but variable lethality that is rescued by the uORF-edited allele. (A) Kidney histology, **(B)** Survival in the cohort of eleven *Pkd1*$^{H\text{-}RW/H\text{-}RW}$ and fifteen *Pkd1*$^{\Delta H\text{-}RW/\Delta H\text{-}RW}$ mice. (C) Representative histology for each genotype. (D) Representative Anti-BiP immunoblot on kidney P9 primary cell lysates illustrating lack of any overt endoplasmic reticulum stress in our humanized and uORF-edited models. Thap: Thapsigargin treatment for 6 hours.

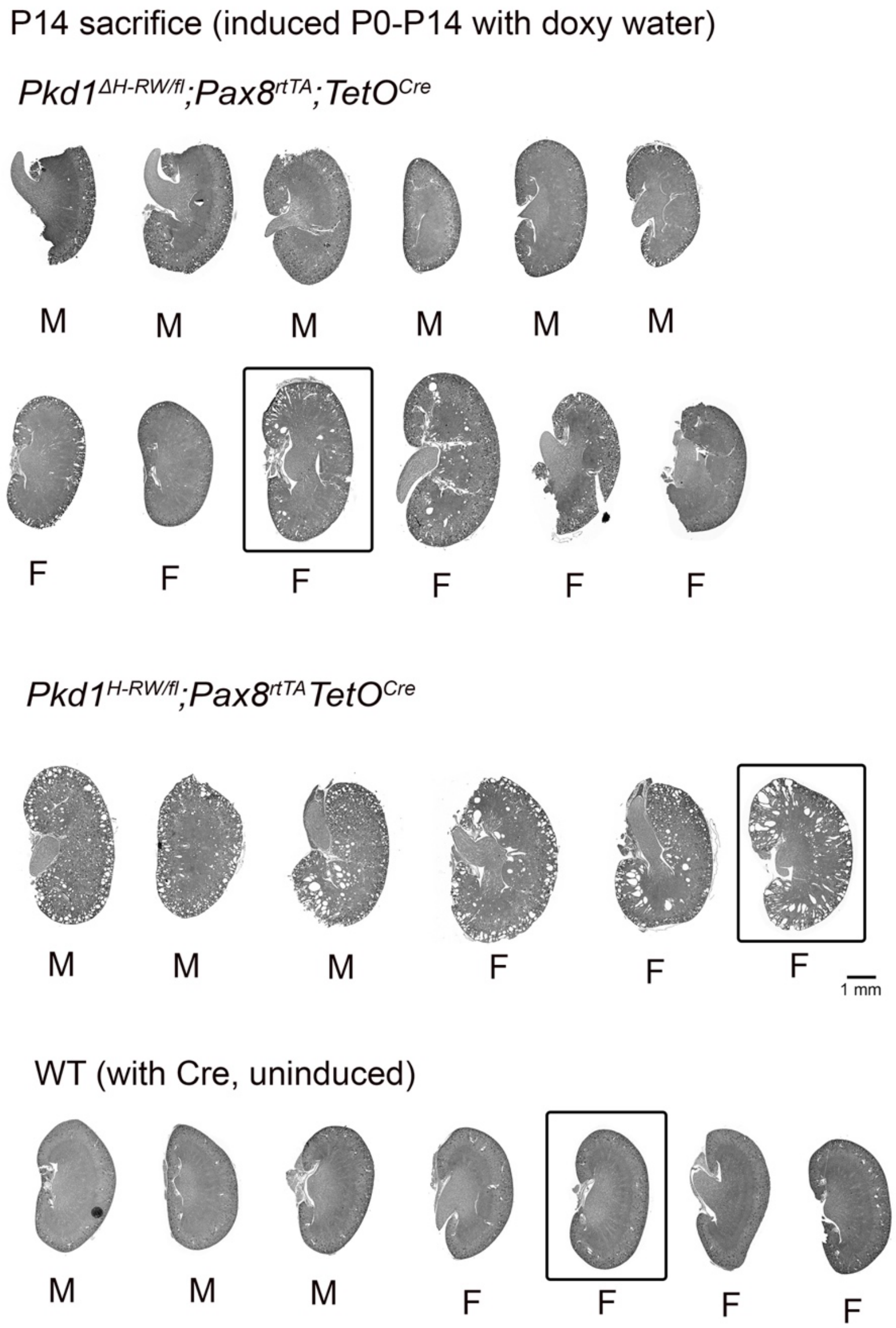

Supplemental Figure 10: P14 timepoint. Kidney scans for all mice in the *Pkd1*^{RW} model analysis sacrificed at P14. Kidneys are to scale with Supplemental Figures 11 and 12. Kidneys from the indicated genotypes are shown. The kidney images in the boxes are also shown in Figure 5B.

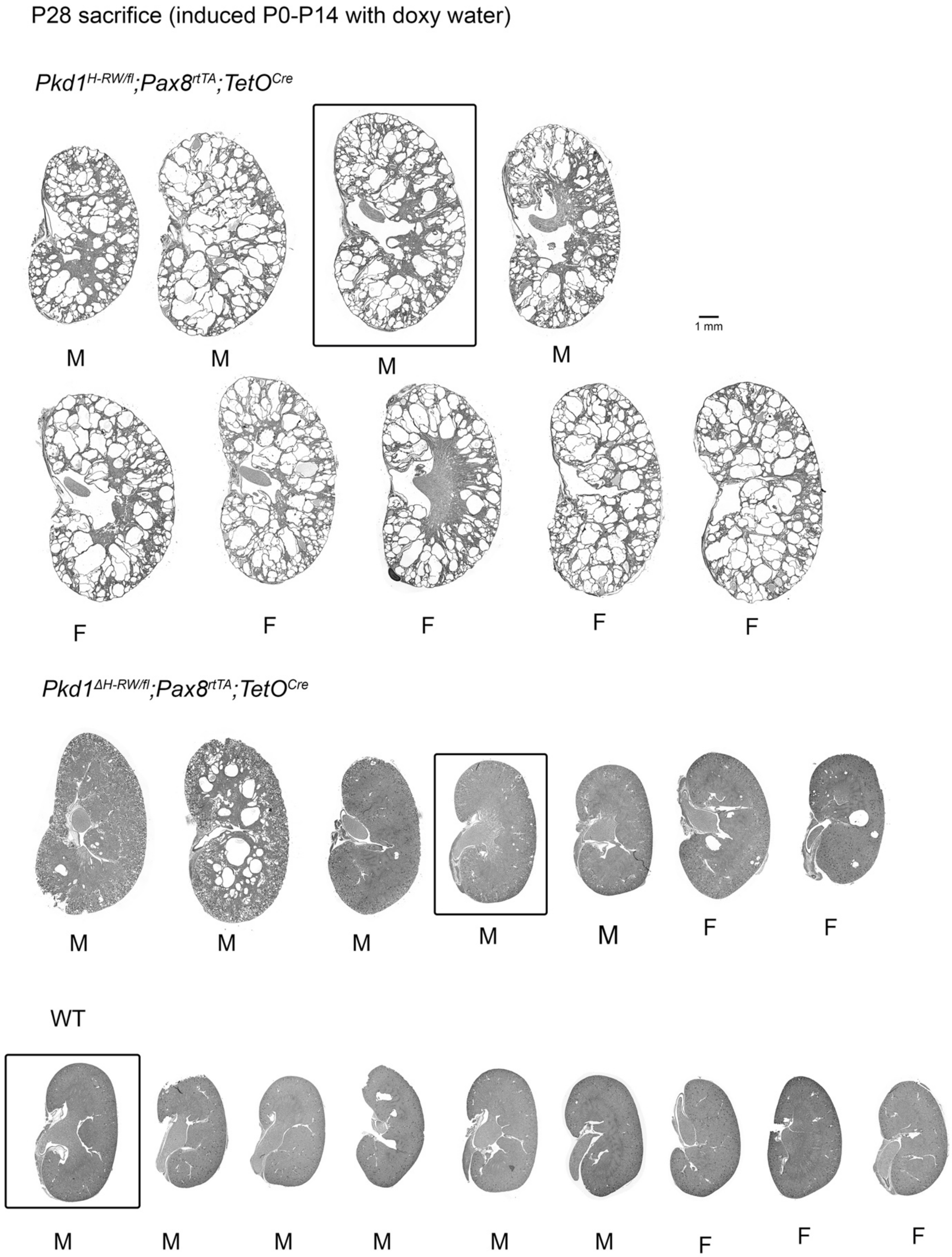

Supplemental Figure 11: P28 timepoint. Kidney scans for all mice in the *Pkd1*^{RW} model analysis, sacrificed at P28. Kidneys from the indicated genotypes are shown. The kidney images in the boxes are also shown in Figure 5C.

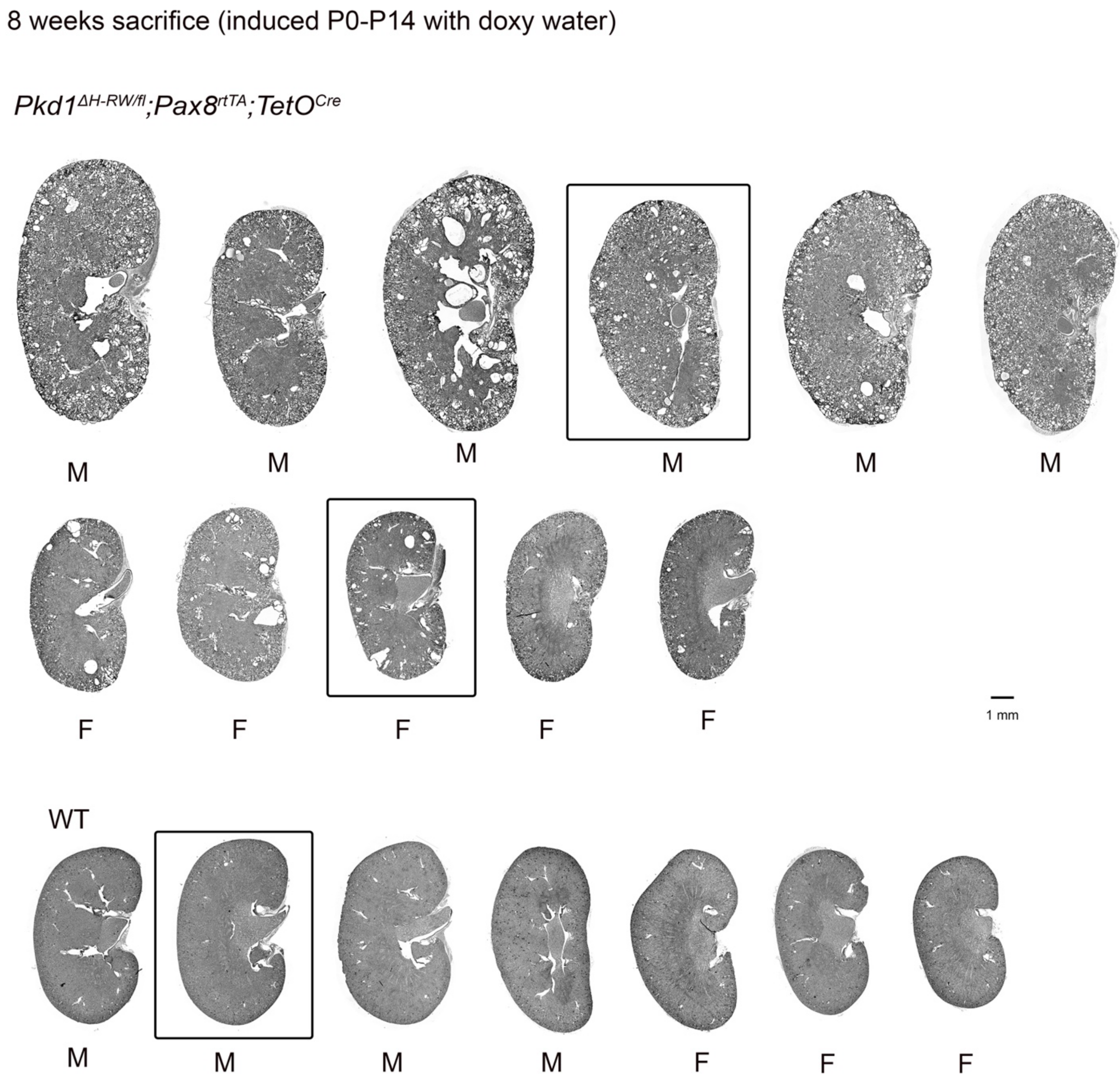

Supplemental Figure 12: **Assessing progression of *Pkd1* missense mice rescued by uORF edit: *$Pkd1^{\Delta H\text{-}RW/fl}$; $Pax8^{rtTA}$;TetO-Cre* at age 8 weeks.** Kidneys from the indicated genotypes are shown. The kidney images in the boxes are also shown in Figure 5D.

Supplemental Table S1. PsiCHECK2 reporter construct insert sequences containing *PKD1* 5'UTR:Renilla

Name	Sequence of insert (5'-3')
wt-*PKD1* 5'UTR	gctagcGCACTGCAGCGCCAGCGTCCGAGCGGGCGGCCGAGCTCCCGGAGCGGCCTGGCCCCG<br>AGCCCCGAGCGGGCGTCGCTCAGCAGCAGGTCGCGGCCGCAGCCCCATCCAGCCCCGCGCCC<br>GCCATGCCGTCCGCGGGCCCCGCCTGAGCTGCGGCCTCCGCGCGCGGGCGGGCCTGGGGAC<br>GGCGGGGCCATGCGCGCGCTGCCCTAACGATGGCTTCCAAGGTGTACGACCCCGAGCAACGCA<br>AACGCATGATCaccggtCCTCAGTGGTGGGCTCGCTGCAAGCAAATGAACGTGCTGGACTCCTTCA<br>TCAACTACTATGATTCCGAGAAGCACGCCGAGAACGCCGTGATTTTTCTGCATGGTAACGCTGCC<br>TCCAGCTACCTGTGGAGGCACGTCGTGCCTCACATCGAGCCCGTGGCTAGATGCATCATCCCTG<br>ATCTGATCGGAATGGGTAAGTCCGGCAAGAGCGGGAATGGCTCATATCGCCTCCTGGATCACTA<br>CAAGTACCTCACCGCTTGGTTCGAGCTGCTGAACCTTCCAAAGAAAATCATCTTTGTGGGCCACG<br>ACTGGGGGGCTTGTCTGGCCTTTCACTACTCCTACGAGCACCAAGACAAGATCAAGGCCATCGT<br>CCATGCTGAGAGTGTCGTGGACGTGATCGAGTCCTGGGACGAGTGGCCTGACATCGAGGAGgat<br>atc…
ΔuORF1	gctagcGCACTGCAGCGCCAGCGTCCGAGCGGGCGGCCGAGCTCCCGGAGCGGCCTGGCCCCG<br>AGCCCCGAGCGGGCGTCGCTCAGCAGCAGGTCGCGGCCGCAGCCCCATCCAGCCCCGCGCCC<br>GCC[T]TGCCGTCCGCGGGCCCCGCCTGAGCTGCGGCCTCCGCGCGCGGGCGGGCCTGGGGA<br>CGGCGGGGCCATGCGCGCGCTGCCCTAACGATGGCTTCCAAGGTGTACGACCCCGAGCAACGC<br>AAACGCATGATCaccggt
ΔuORF2	gctagcGCACTGCAGCGCCAGCGTCCGAGCGGGCGGCCGAGCTCCCGGAGCGGCCTGGCCCCG<br>AGCCCCGAGCGGGCGTCGCTCAGCAGCAGGTCGCGGCCGCAGCCCCATCCAGCCCCGCGCCC<br>GCCATGCCGTCCGCGGGCCCCGCCTGAGCTGCGGCCTCCGCGCGCGGGCGGGCCTGGGGAC<br>GGCGGGGCC[T]TGCGCGCGCTGCCCTAACGATGGCTTCCAAGGTGTACGACCCCGAGCAACGC<br>AAACGCATGATCaccggt
ΔuORF3	gctagcGCACTGCAGCGCCAGCGTCCGAGCGGGCGGCCGAGCTCCCGGAGCGGCC[C]GGCCCC<br>GAGCCCCGAGCGGGCGTCGCTCAGCAGCAGGTCGCGGCCGCAGCCCCATCCAGCCCCGCGCC<br>CGCCATGCCGTCCGCGGGCCCCGCCTGAGCTGCGGCCTCCGCGCGCGGGCGGGCCTGGGGA<br>CGGCGGGGCCATGCGCGCGCTGCCCTAACGATGGCTTCCAAGGTGTACGACCCCGAGCAACGC<br>AAACGCATGATCaccggt
ΔuORF1&2	gctagcGCACTGCAGCGCCAGCGTCCGAGCGGGCGGCCGAGCTCCCGGAGCGGCCTGGCCCCG<br>AGCCCCGAGCGGGCGTCGCTCAGCAGCAGGTCGCGGCCGCAGCCCCATCCAGCCCCGCGCCC<br>GCC[T]TGCCGTCCGCGGGCCCCGCCTGAGCTGCGGCCTCCGCGCGCGGGCGGGCCTGGGGA<br>CGGCGGGGCC[T]TGCGCGCGCTGCCCTAACGATGGCTTCCAAGGTGTACGACCCCGAGCAACG<br>CAAACGCATGATCaccggt
ΔuORF4	gctagcGCA[T]TGCAGCGCCAGCGTCCGAGCGGGCGGCCGAGCTCCCGGAGCGGCCTGGCCCCG<br>AGCCCCGAGCGGGCGTCGCTCAGCAGCAGGTCGCGGCCGCAGCCCCATCCAGCCCCGCGCCC<br>GCCATGCCGTCCGCGGGCCCCGCCTGAGCTGCGGCCTCCGCGCGCGGGCGGGCCTGGGGAC<br>GGCGGGGCCATGCGCGCGCTGCCCTAACGATGGCTTCCAAGGTGTACGACCCCGAGCAACGCA<br>AACGCATGATCaccggt
ΔuORF1&2 &4	gctagcGCA[T]TGCAGCGCCAGCGTCCGAGCGGGCGGCCGAGCTCCCGGAGCGGCCTGGCCCCG<br>AGCCCCGAGCGGGCGTCGCTCAGCAGCAGGTCGCGGCCGCAGCCCCATCCAGCCCCGCGCCC<br>GCC[T]TGCCGTCCGCGGGCCCCGCCTGAGCTGCGGCCTCCGCGCGCGGGCGGGCCTGGGGA<br>CGGCGGGGCC[T]TGCGCGCGCTGCCCTAACGATGGCTTCCAAGGTGTACGACCCCGAGCAACG<br>CAAACGCATGATCaccggt
ΔuORF1&2 &3&4	gctagcGCA[T]TGCAGCGCCAGCGTCCGAGCGGGCGGCCGAGCTCCCGGAGCGGC[T]TGGCCCC<br>GAGCCCCGAGCGGGCGTCGCTCAGCAGCAGGTCGCGGCCGCAGCCCCATCCAGCCCCGCGCC<br>CGCC[T]TGCCGTCCGCGGGCCCCGCCTGAGCTGCGGCCTCCGCGCGCGGGCGGGCCTGGGG<br>ACGGCGGGGCC[T]TGCGCGCGCTGCCCTAACGATGGCTTCCAAGGTGTACGACCCCGAGCAAC<br>GCAAACGCATGATCaccggt

Fus1	gctagcGCACTGCAGCGCCAGCGTCCGAGCGGGCGGCCGAGCTCCCGGAGCGGCCTGGCCCCG<br>AGCCCCGAGCGGGCGTCGCTCAGCAGCAGGTCGCGGCCGCAGCCCCATCCAGCCCCGCGCCC<br>GCCATGCCGTCCGCGGGCCCCGCCT**[AC]**GCTGCGGCCTCCGCGCGCGGGCGGGCCTGGGGA<br>CGGCGGGGCCATGCGCGCGCTGCCCTAACG**[T]**TGGCTTCCAAGGTGTACGACCCCGAGCAACG<br>CAAACGCATGATCaccggt…
Fus1_NC	gctagcGCACTGCAGCGCCAGCGTCCGAGCGGGCGGCCGAGCTCCCGGAGCGGCCTGGCCCCG<br>AGCCCCGAGCGGGCGTCGCTCAGCAGCAGGTCGCGGCCGCAGCCCCATCCAGCCCCGCGCCC<br>GCC**[T]**TGCCGTCCGCGGGCCCCGCCT**[AC]**GCTGCGGCCTCCGCGCGCGGGCGGGCCTGGGGA<br>CGGCGGGGCCATGCGCGCGCTGCCCTAACG**[T]**TGGCTTCCAAGGTGTACGACCCCGAGCAACG<br>CAAACGCATGATCaccggt…
Fus2	gctagcGCACTGCAGCGCCAGCGTCCGAGCGGGCGGCCGAGCTCCCGGAGCGGCCTGGCCCCG<br>AGCCCCGAGCGGGCGTCGCTCAGCAGCAGGTCGCGGCCGCAGCCCCATCCAGCCCCGCGCCC<br>GCCATGCCGTCCGCGGGCCCCGCCTGAGCTGCGGCCTCCGCGCGCGGGCGGGCCTGGGGAC<br>GGCGGGGCCATGCGCGCGCTGCCCTA**[CT]**CG**[T]**TGGCTTCCAAGGTGTACGACCCCGAGCAAC<br>GCAAACGCATGATCaccggt…
Fus2_NC	gctagcGCACTGCAGCGCCAGCGTCCGAGCGGGCGGCCGAGCTCCCGGAGCGGCCTGGCCCCG<br>AGCCCCGAGCGGGCGTCGCTCAGCAGCAGGTCGCGGCCGCAGCCCCATCCAGCCCCGCGCCC<br>GCCATGCCGTCCGCGGGCCCCGCCTGAGCTGCGGCCTCCGCGCGCGGGCGGGCCTGGGGAC<br>GGCGGGGCC**[T]**TGCGCGCGCTGCCCTA**[CT]**CG**[T]**TGGCTTCCAAGGTGTACGACCCCGAGCAA<br>CGCAAACGCATGATCaccggt…
Fus2_<br>ΔuORF1	gctagcGCACTGCAGCGCCAGCGTCCGAGCGGGCGGCCGAGCTCCCGGAGCGGCCTGGCCCCG<br>AGCCCCGAGCGGGCGTCGCTCAGCAGCAGGTCGCGGCCGCAGCCCCATCCAGCCCCGCGCCC<br>GCC**[T]**TGCCGTCCGCGGGCCCCGCCTGAGCTGCGGCCTCCGCGCGCGGGCGGGCCTGGGGA<br>CGGCGGGGCCATGCGCGCGCTGCCCTA**[CT]**CG**[T]**TGGCTTCCAAGGTGTACGACCCCGAGCAA<br>CGCAAACGCATGATCaccggt…
Fus2_<br>ΔuORF3	gctagcGCACTGCAGCGCCAGCGTCCGAGCGGGCGGCCGAGCTCCCGGAGCGGC**[T]**TGGCCCCG<br>AGCCCCGAGCGGGCGTCGCTCAGCAGCAGGTCGCGGCCGCAGCCCCATCCAGCCCCGCGCCC<br>GCCATGCCGTCCGCGGGCCCCGCCTGAGCTGCGGCCTCCGCGCGCGGGCGGGCCTGGGGAC<br>GGCGGGGCCATGCGCGCGCTGCCCTA**[CT]**CG**[T]**TGGCTTCCAAGGTGTACGACCCCGAGCAAC<br>GCAAACGCATGATCaccggt…
Fus2_<br>ΔuORF4	gctagcGCA**[T]**TGCAGCGCCAGCGTCCGAGCGGGCGGCCGAGCTCCCGGAGCGGCCTGGCCCCG<br>AGCCCCGAGCGGGCGTCGCTCAGCAGCAGGTCGCGGCCGCAGCCCCATCCAGCCCCGCGCCC<br>GCCATGCCGTCCGCGGGCCCCGCCTGAGCTGCGGCCTCCGCGCGCGGGCGGGCCTGGGGAC<br>GGCGGGGCCATGCGCGCGCTGCCCTA**[CT]**CG**[T]**TGGCTTCCAAGGTGTACGACCCCGAGCAAC<br>GCAAACGCATGATCaccggt…
Fus2_<br>ΔuORF3&4	gctagcGCA**[T]**TGCAGCGCCAGCGTCCGAGCGGGCGGCCGAGCTCCCGGAGCGGC**[T]**TGGCCCC<br>GAGCCCCGAGCGGGCGTCGCTCAGCAGCAGGTCGCGGCCGCAGCCCCATCCAGCCCCGCGCC<br>CGCCATGCCGTCCGCGGGCCCCGCCTGAGCTGCGGCCTCCGCGCGCGGGCGGGCCTGGGGA<br>CGGCGGGGCCATGCGCGCGCTGCCCTA**[CT]**CG**[T]**TGGCTTCCAAGGTGTACGACCCCGAGCAA<br>CGCAAACGCATGATCaccggt…
Fus2_<br>ΔuORF1&3<br>&4	gctagcGCA**[T]**TGCAGCGCCAGCGTCCGAGCGGGCGGCCGAGCTCCCGGAGCGGC**[T]**TGGCCCC<br>GAGCCCCGAGCGGGCGTCGCTCAGCAGCAGGTCGCGGCCGCAGCCCCATCCAGCCCCGCGCC<br>CGCC**[T]**TGCCGTCCGCGGGCCCCGCCTGAGCTGCGGCCTCCGCGCGCGGGCGGGCCTGGGG<br>ACGGCGGGGCCATGCGCGCGCTGCCCTA**[CT]**CG**[T]**TGGCTTCCAAGGTGTACGACCCCGAGCA<br>ACGCAAACGCATGATCaccggt…
Fus1_NS<br>(NS=No<br>stop, to<br>model<br>uORF1 stop<br>codon<br>readthrough<br>)	gctagcGCACTGCAGCGCCAGCGTCCGAGCGGGCGGCCGAGCTCCCGGAGCGGCCTGGCCCCG<br>AGCCCCGAGCGGGCGTCGCTCAGCAGCAGGTCGCGGCCGCAGCCCCATCCAGCCCCGCGCCC<br>GCCATGCCGTCCGCGGGCCCCGCCT**[AC]**GCTGCGGCCTCCGCGCGCGGGCGGGCCTGGGGA<br>CGGCGGGGCCATGCGCGCGCTGCCCTAACGATGGCTTCCAAGGTGTACGACCCCGAGCAACGC<br>AAACGCATGATCaccggt…

Fus1_NS_ ΔuORF3&4	gctagcGCA**[T]**TGCAGCGCCAGCGTCCGAGCGGGCGGCCGAGCTCCCGGAGCGGC**[T]**TGGCCCC GAGCCCCGAGCGGGCGTCGCTCAGCAGCAGGTCGCGGCCGCAGCCCCATCCAGCCCCGCGCC CGCCATGCCGTCCGCGGGCCCCGCCT**[AC]**GCTGCGGCCTCCGCGCGCGGGCGGCCTGGGG ACGGCGGGGCCATGCGCGCGCTGCCCTAACGATGGCTTCCAAGGTGTACGACCCCGAGCAACG CAAACGCATGATCaccggt…

Lowercase font: relevant restriction enzyme sequence. Red/Bold/bracketed font: edited nucleotides compared to Wt-*PKD1* 5'UTR. Orange Font: Renilla luciferase sequence. Underline: initiation codons for uORF3, uORF1, uORF2, Renilla in that order. NC, Negative control. NS, non-stop codon(stop codon edited).

Supplemental Table S2. ASO sequences used in this study.

Name	5'-3' oligonucleotide sequence (reverse complement of target mRNA sequence, 16 nucleotides each)
Human ASO1	mC*mA*mU*mG*mG*mC*mG*mG*mG*mC*mG*mC*mG*mG*mG*mG
Human mismatched control-1 (MM1)	mC*m**U***mU*mG*m**C***mC*mG*m**C***mG*mC*m**C***mC*mG*m**C***mG*mG
Human ASO2	mC*mA*mU*mG*mG*mC*mC*mC*mC*mG*mC*mC*mG*mU*mC*mC
Human mismatched control-2 (MM2)	mC*m**U***mU*mG*m**C***mC*mC*m**G***mC*mG*m**G***mC*mG*m**A***mC*mC
Mouse mASO1	mC*mA*mU*mG*mG*mU*mG*mC*mG*mG*mC*mA*mC*mG*mG*mG
Mouse mismatched control-1 (mMM1)	mC*m**U***mU*mG*m**C***mU*mG*m**G***mG*mG*m**G***mA*mC*m**C***mG*mG
Mouse mASO2	mC*mA*mU*mG*mG*mC*mC*mC*mC*mG*mG*mU*mU*mC*mC*mC
Mouse mismatched control-2 (mMM2)	mC*m**U***mU*mG*m**C***mC*mC*m**G***mC*mG*m**C***mU*mU*m**G***mC*mC
Human ASO3	mC*mA*mG*mG*mC*mC*mG*mC*mU*mC*mC*mG*mG*mG*mA*mG
Human mismatched control-3(mMM3)	mC*m**U***mG*mG*m**G***mC*mG*m**G***mU*mC*m**G***mG* mG*m**C***mA*mG

m: 2′-O-methyl (Me) modification, *: Phosphorothioate (PS) linkage. Red/Bold: differences of mismatched control ASOs with comparison experimental ASO sequence.

Supplemental Table S3. qPCR Primer sequences used in this study.

Human *PKD1*	Forward: GTCCGTCTTTGGCAAGACATT
	Reverse: ACAGGAAGACACGAGCAGGA
Mouse *Pkd1*	Forward: AGGAGTTCCGCCACAAAGTC
	Reverse: AGCTGGTAGACGTCCTCTGT
Human *GAPDH*	Forward: ACAACTTTGGTATCGTGGAAGG
	Reverse: GCCATCACGCCACAGTTTC
Mouse *Gapdh*	Forward: AACCCTTAAGAGGGATGCTGC
	Reverse: TCTACGGGACGAGGAAACAC
Mouse *18s*	Forward: GTAACCCGTTGAACCCCATT
	Reverse: CCATCCAATCGGTAGTAGCG
Renilla	Forward: GCGACGATCTGCCTAAGATGT
	Reverse: CGACAATAGCGTTGGAAAAGAA
Firefly	Forward: TAACCCTGAGGCCAVAAACG
	Reverse: TCCACGATGAAGAAGTGCTCGT
