# Supplementary material for: PKD1 upstream open reading frames affect Polycystin-1 expression and polycystic kidney disease phenotypes
Source: J Clin Invest. 2026 Jul 28;136(18):e203177. doi: 10.1172/JCI203177 (PMC13574166; doi:10.1172/JCI203177)

Figure 1F

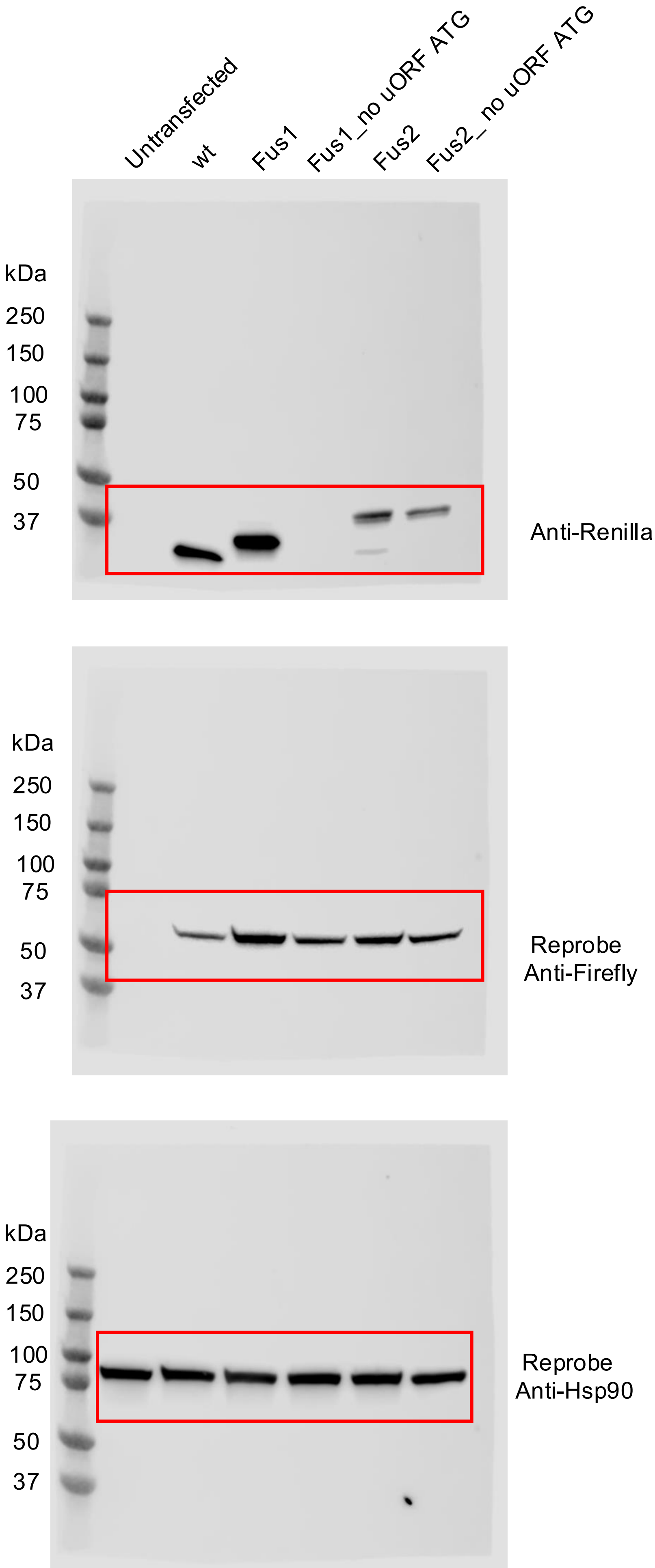
Untransfected
wt
Fus1
Fus1_no uORF ATG
Fus2
Fus2_ no uORF ATG
kDa
250
150
100
75
50
37
Anti-Renilla
kDa
250
150
100
75
50
37
Reprobe
Anti-Firefly
kDa
250
150
100
75
50
37
Reprobe
Anti-Hsp90

Figure 1H

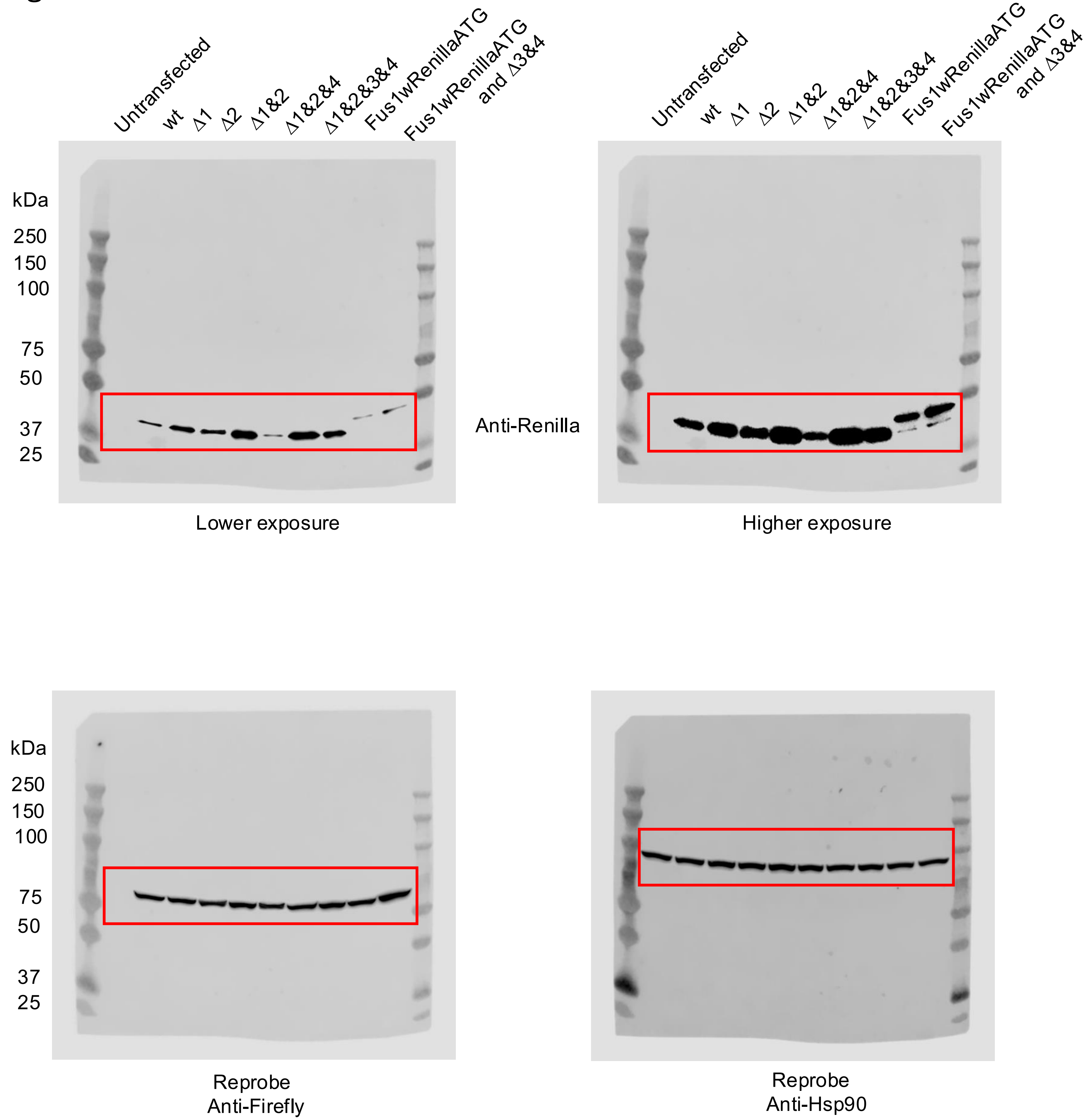
Untransfected
wt
Δ1
Δ2
Δ1&2
Δ1&2&4
Δ1&2&3&4
Fus1wRenillaATG
Fus1wRenillaATG and Δ3&4
kDa
250
150
100
75
50
37
25
Anti-Renilla
Lower exposure
Higher exposure
Reprobe Anti-Firefly
Reprobe Anti-Hsp90

Figure 2E

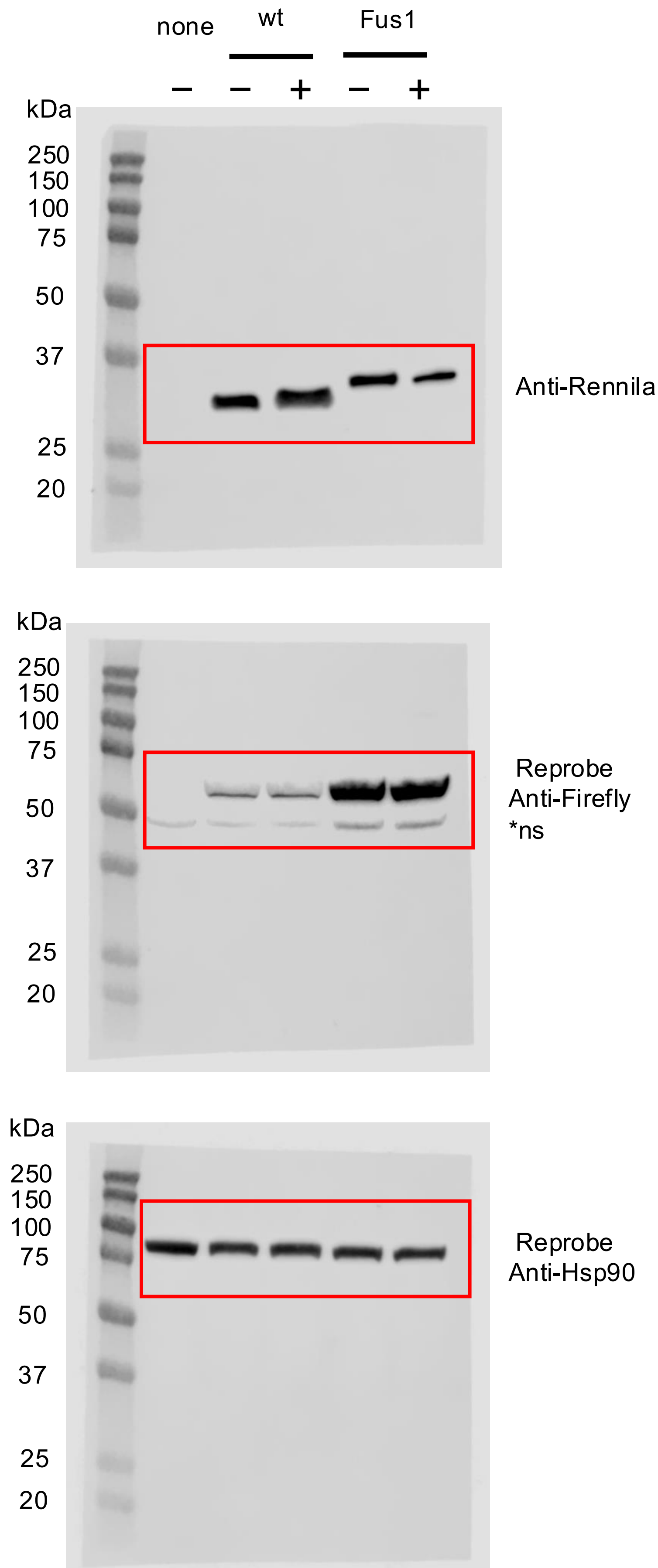

none
wt
Fus1
–
–
+
–
+
kDa
250
150
100
75
50
37
25
20
Anti-Rennila
kDa
250
150
100
75
50
37
25
20
Reprobe
Anti-Firefly
*ns
kDa
250
150
100
75
50
37
25
20
Reprobe
Anti-Hsp90

Figure 3A

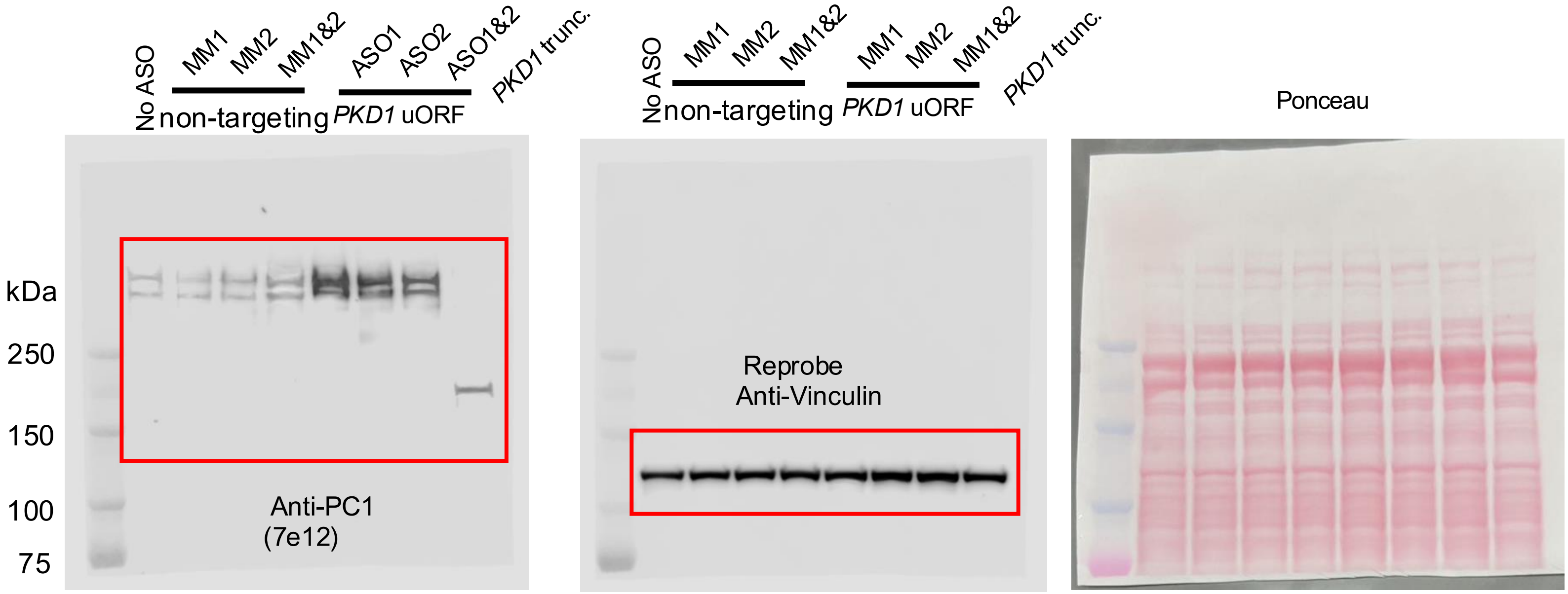

Figure 3B

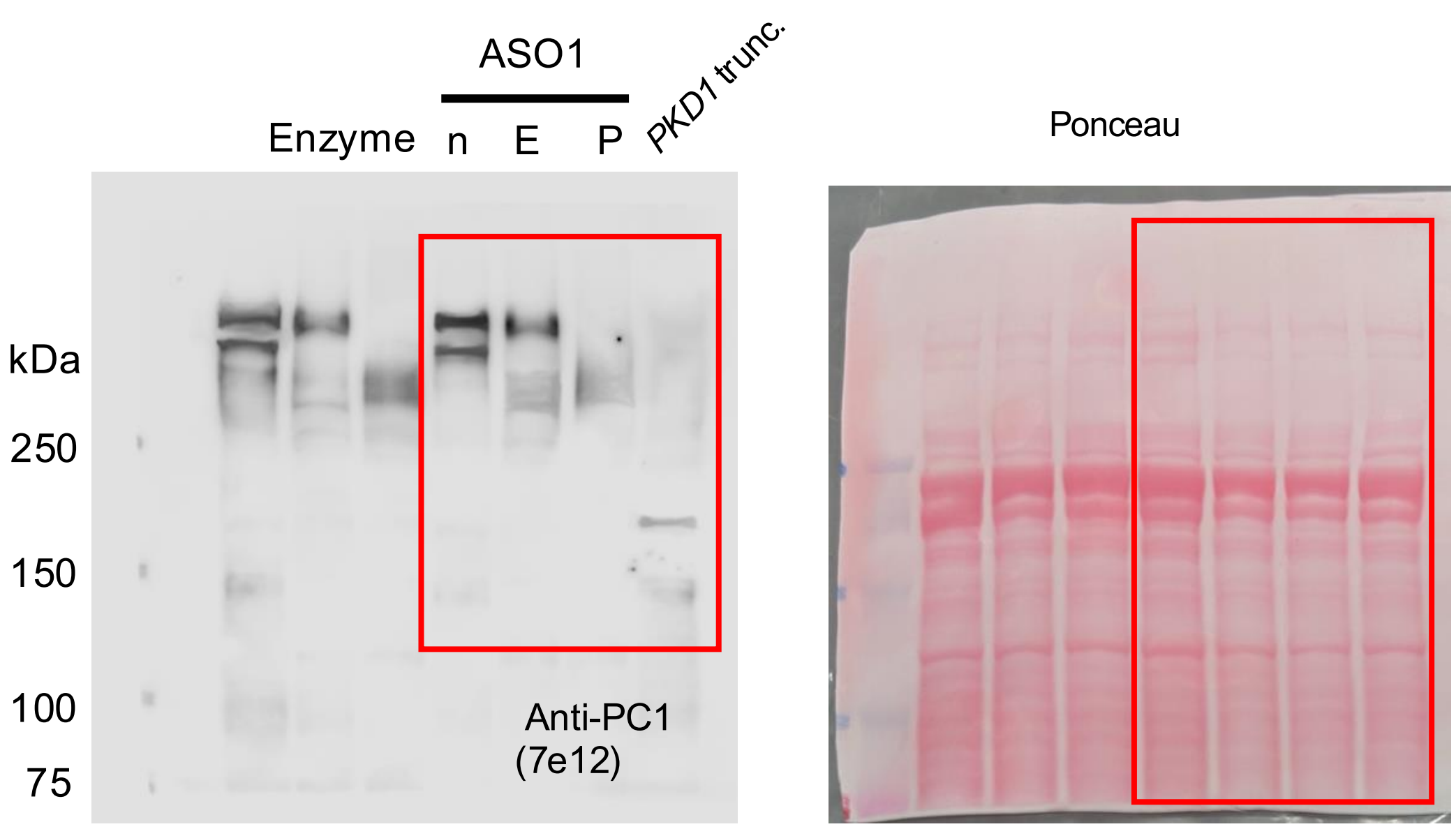

Figure 3D

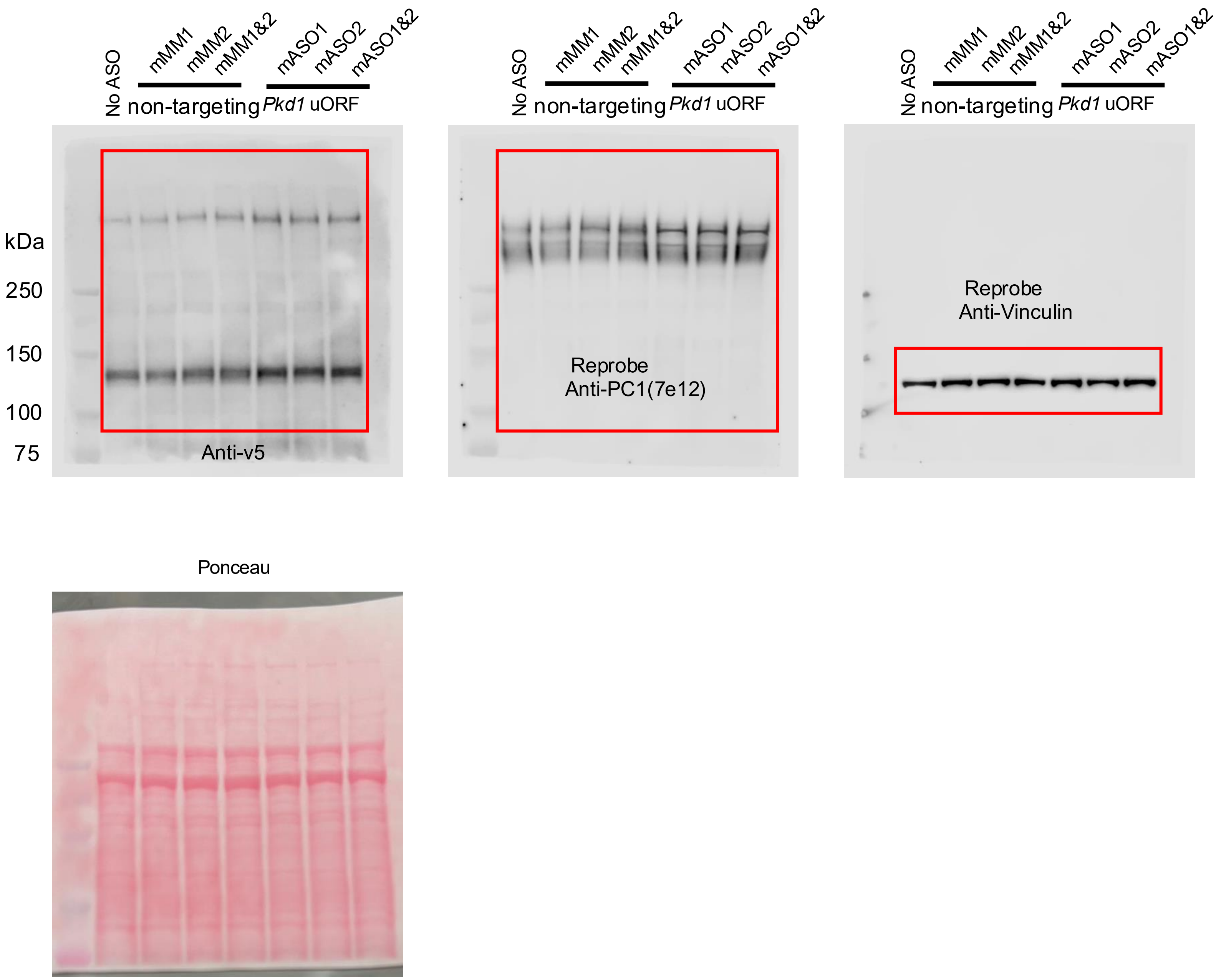
No ASO
mMM1
mMM2
mMM1&2
mASO1
mASO2
mASO1&2
non-targeting
Pkd1 uORF
kDa
250
150
100
75
Anti-v5
Reprobe
Anti-PC1(7e12)
Reprobe
Anti-Vinculin
Ponceau

Figure 3E

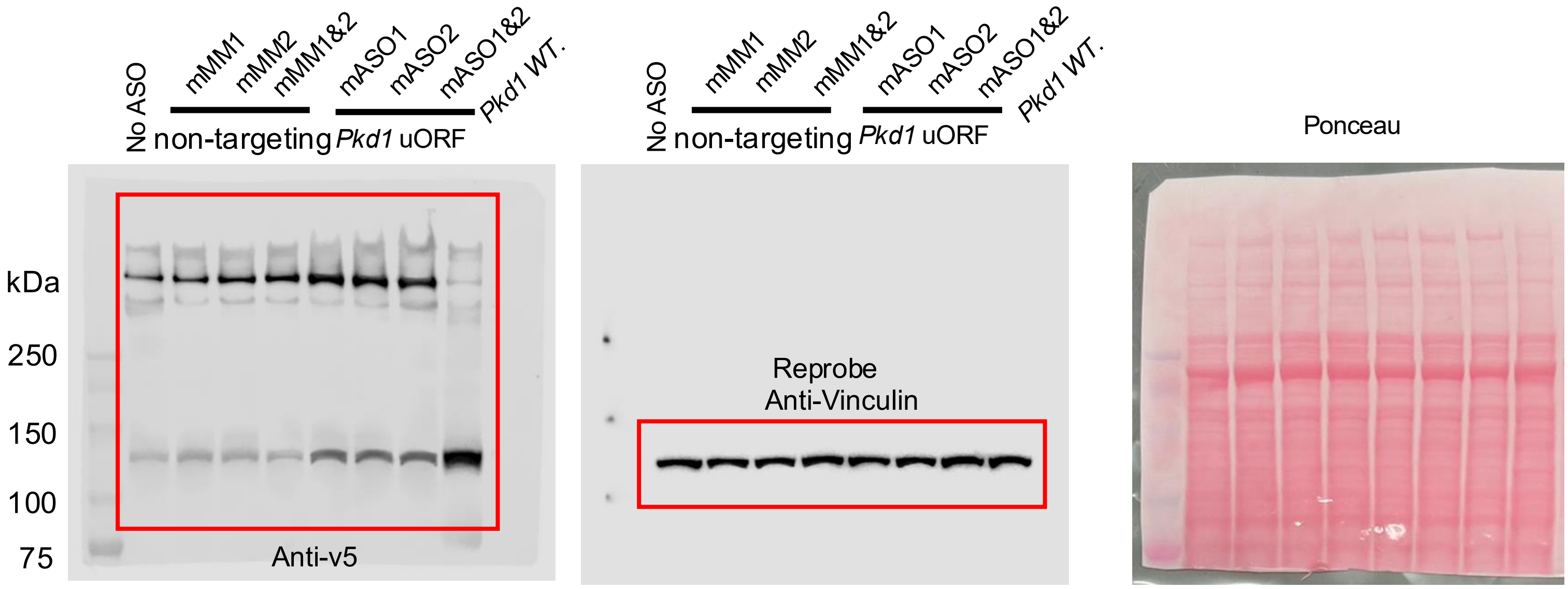

No ASO
mMM1
mMM2
mMM1&2
mASO1
mASO2
mASO1&2
Pkd1 WT.
non-targeting
Pkd1 uORF
kDa
250
150
100
75
Anti-v5
Reprobe
Anti-Vinculin
Ponceau

Figure 4C

Anti-rabbit V5

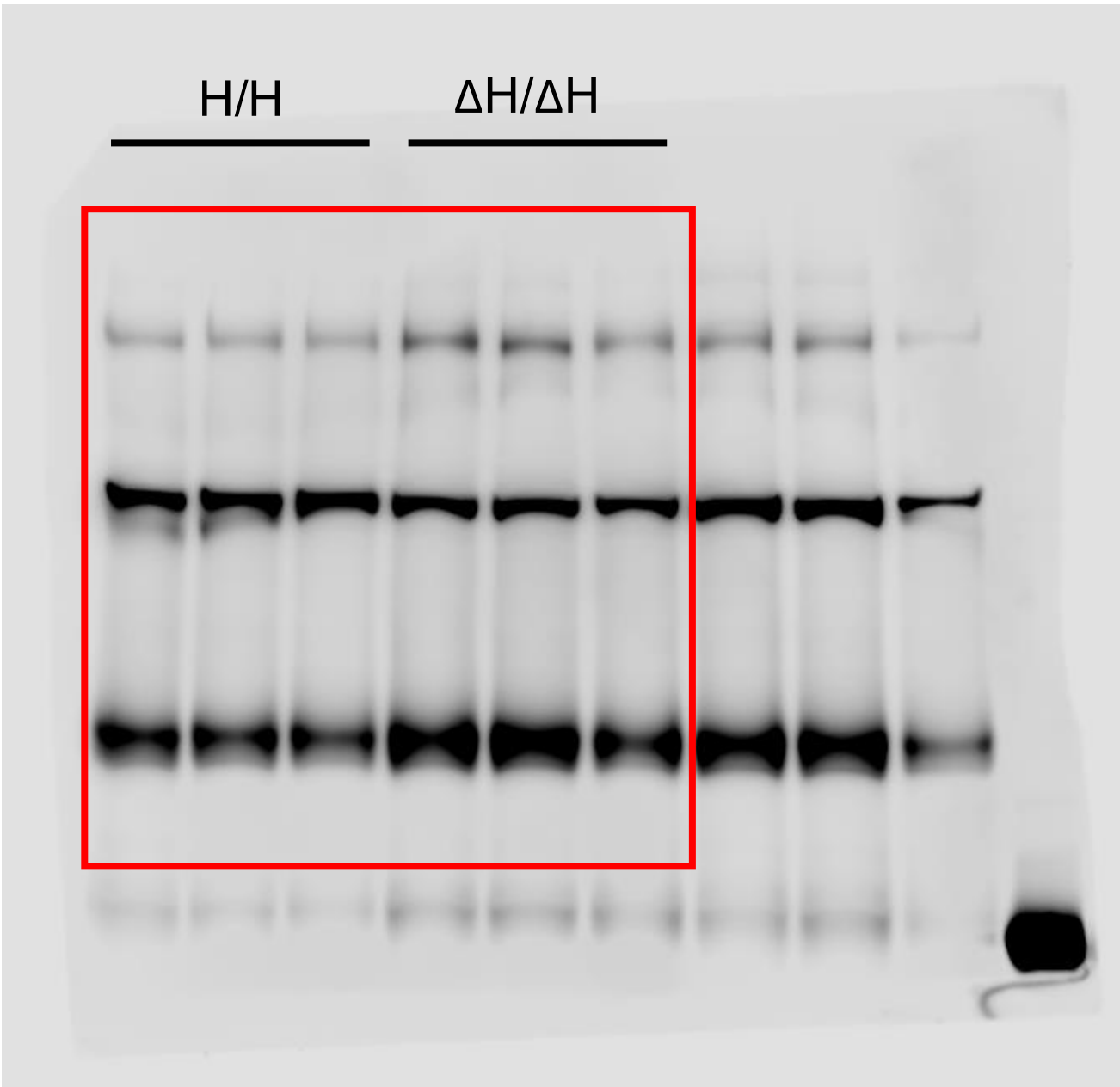

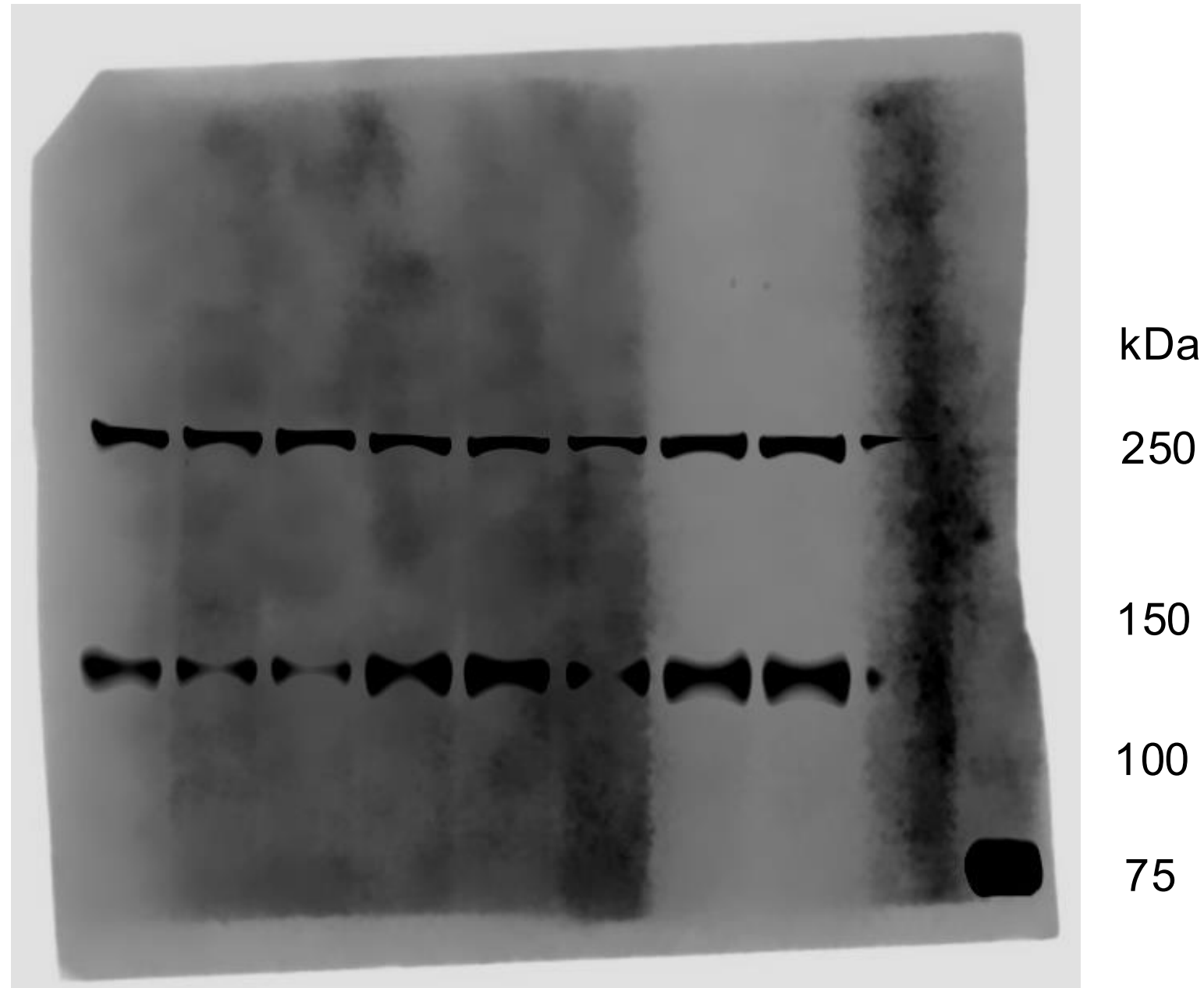

Same blot as left to show ladder

Reprobe
Anti-Vinculin

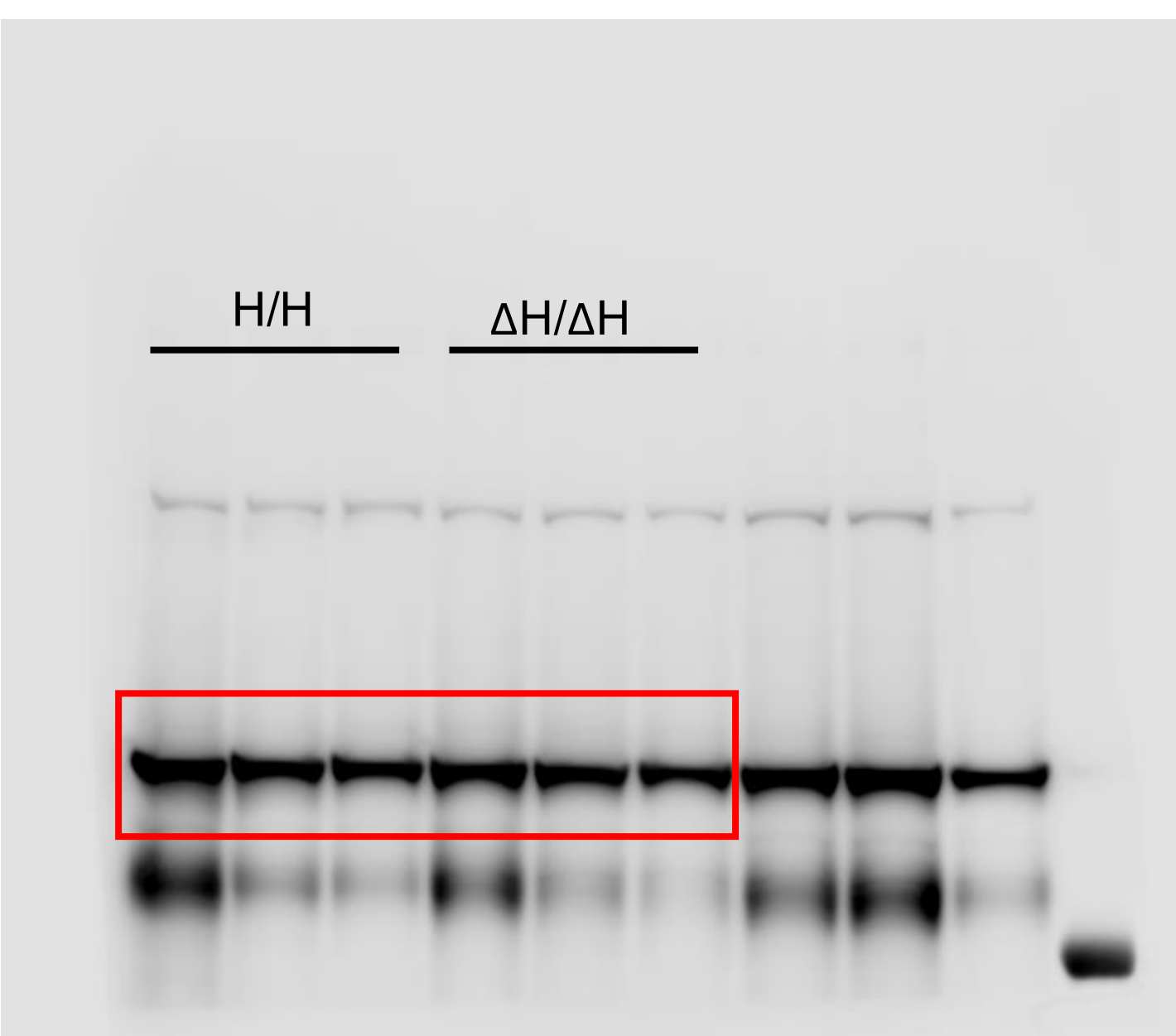

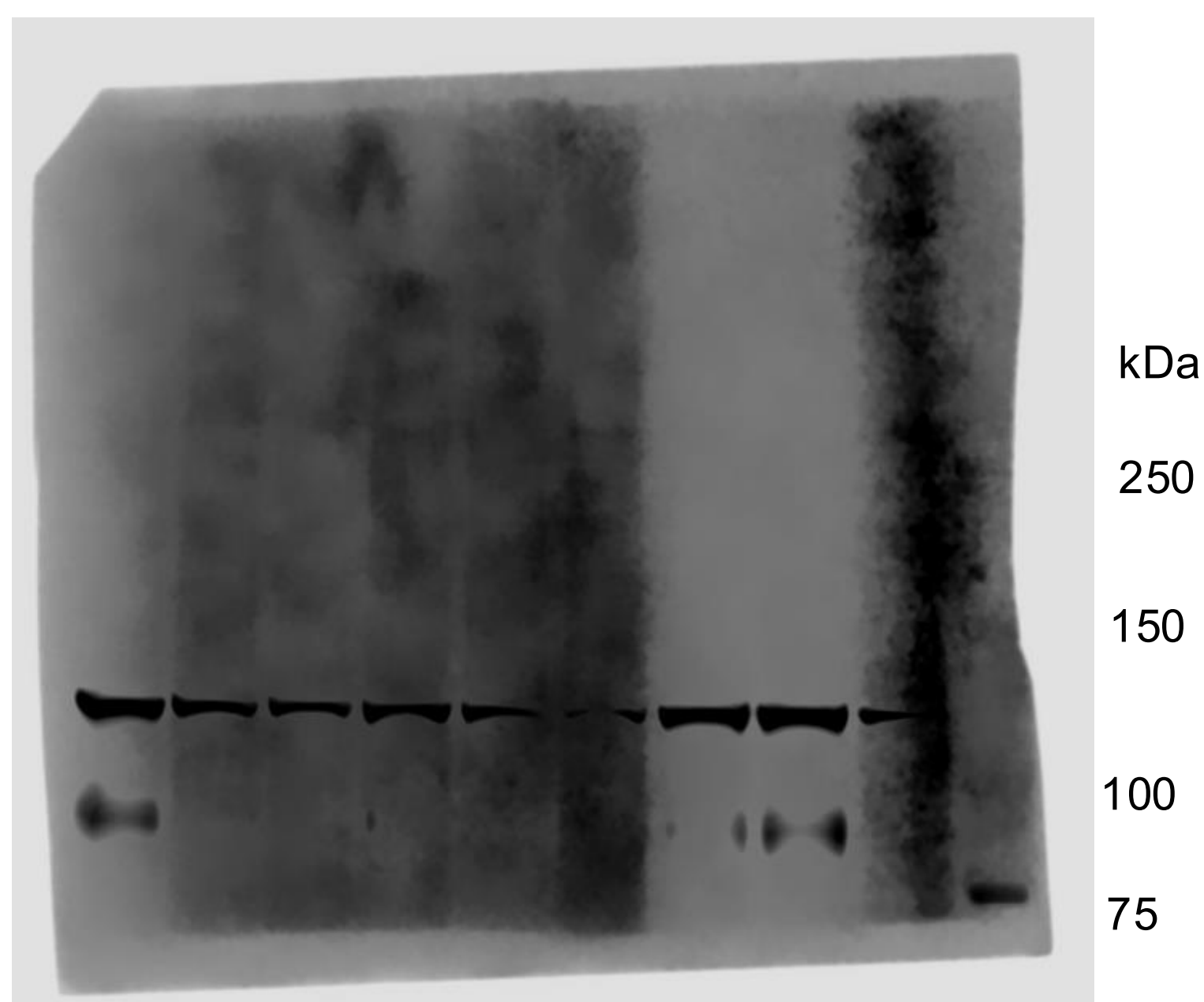

Same blot as left to show ladder

Figure 4D

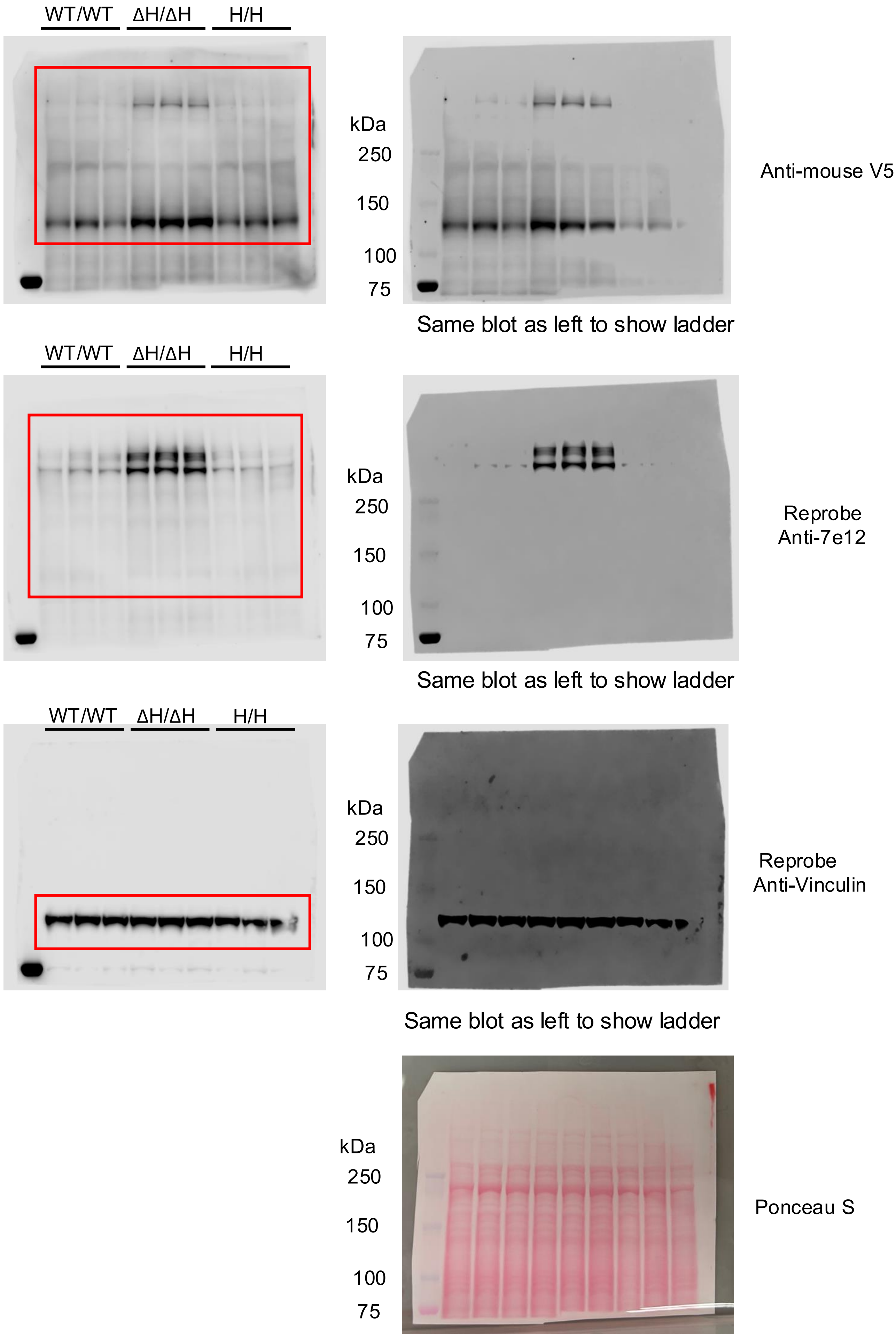
WT/WT
ΔH/ΔH
H/H
kDa
250
150
100
75
Anti-mouse V5
Same blot as left to show ladder
WT/WT
ΔH/ΔH
H/H
kDa
250
150
100
75
Reprobe
Anti-7e12
Same blot as left to show ladder
WT/WT
ΔH/ΔH
H/H
kDa
250
150
100
75
Reprobe
Anti-Vinculin
Same blot as left to show ladder
kDa
250
150
100
75
Ponceau S

Figure 4E

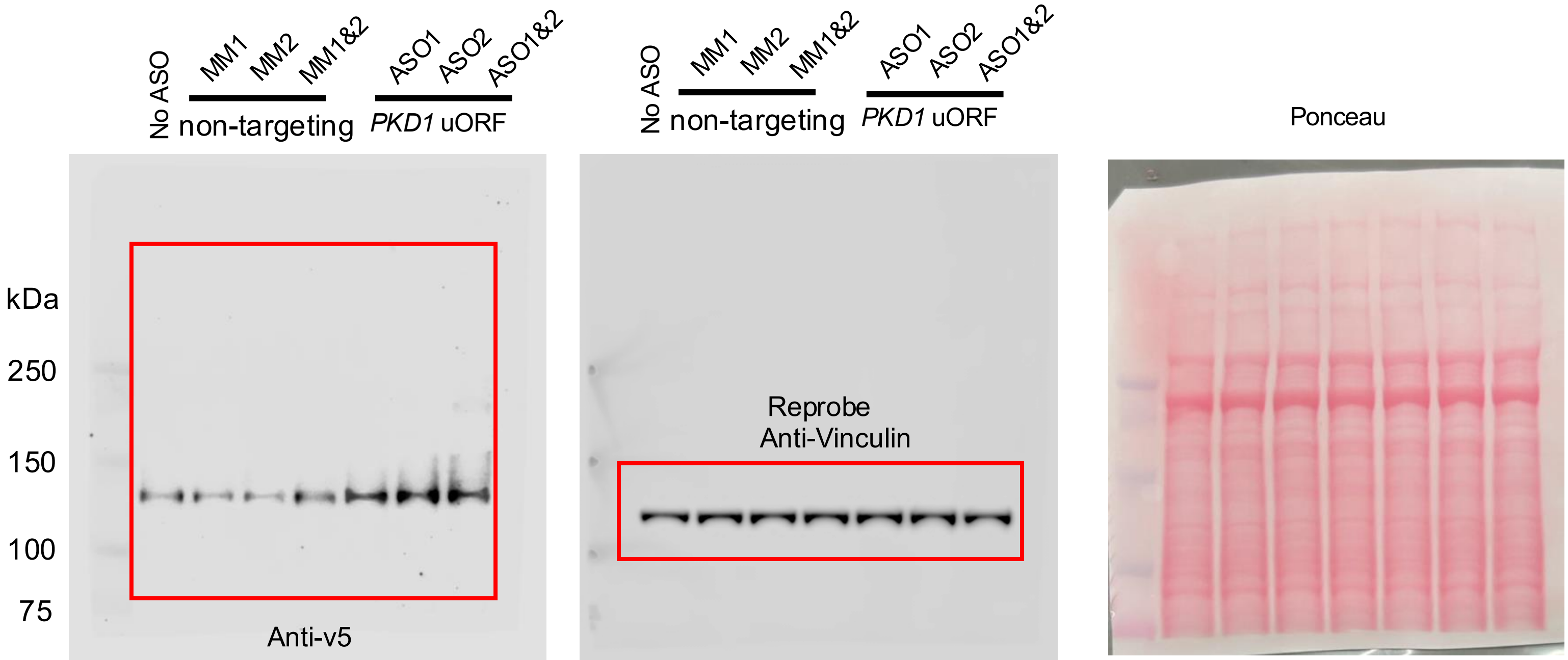
No ASO
MM1
MM2
MM1&2
ASO1
ASO2
ASO1&2
non-targeting
PKD1 uORF
kDa
250
150
100
75
Anti-v5
No ASO
MM1
MM2
MM1&2
ASO1
ASO2
ASO1&2
non-targeting
PKD1 uORF
Reprobe
Anti-Vinculin
Ponceau

Figure 5A

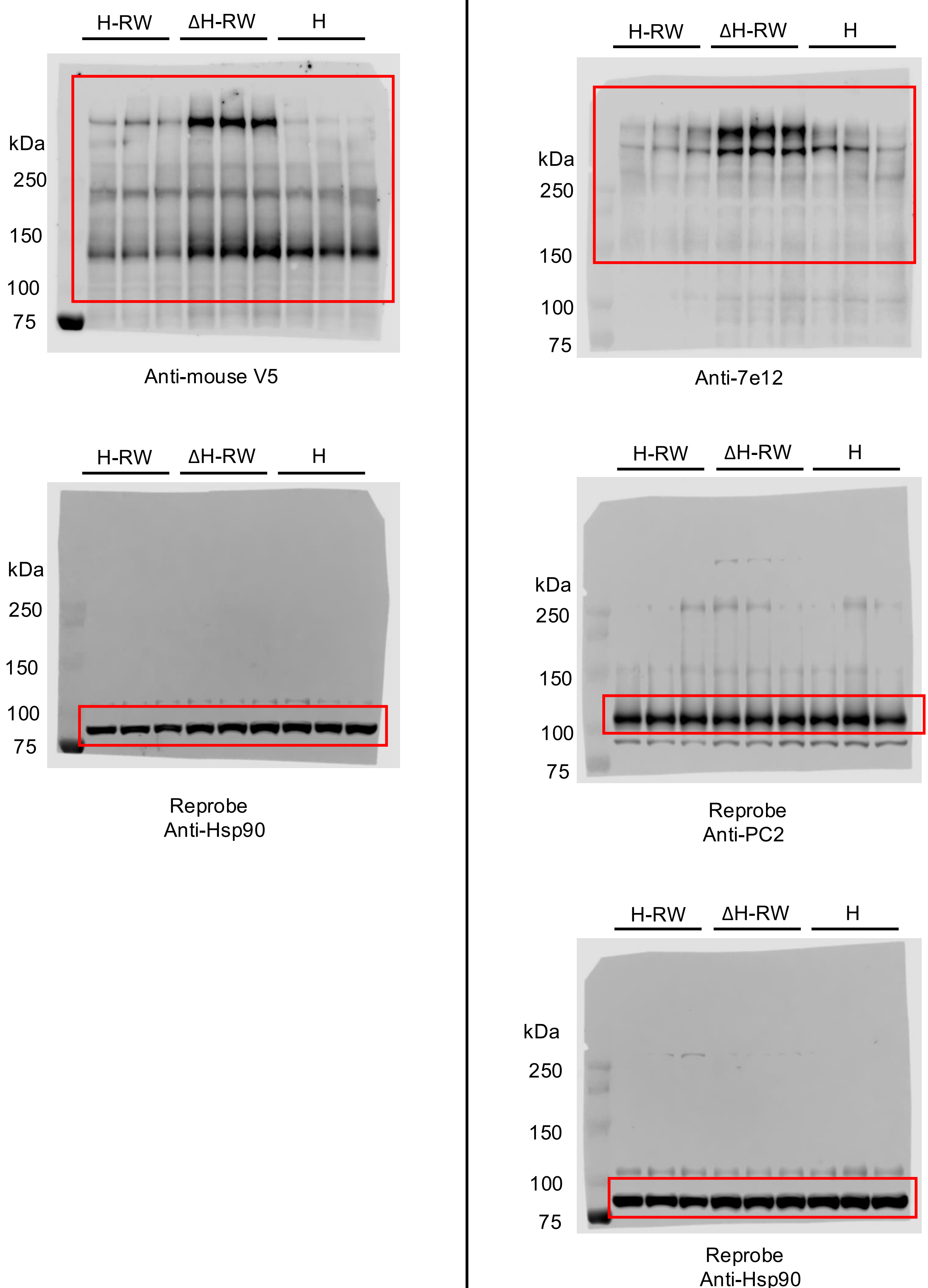
H-RW
ΔH-RW
H
kDa
250
150
100
75
Anti-mouse V5
H-RW
ΔH-RW
H
kDa
250
150
100
75
Anti-7e12
H-RW
ΔH-RW
H
kDa
250
150
100
75
Reprobe
Anti-Hsp90
H-RW
ΔH-RW
H
kDa
250
150
100
75
Reprobe
Anti-PC2
H-RW
ΔH-RW
H
kDa
250
150
100
75
Reprobe
Anti-Hsp90

Supplemental Figure 4C

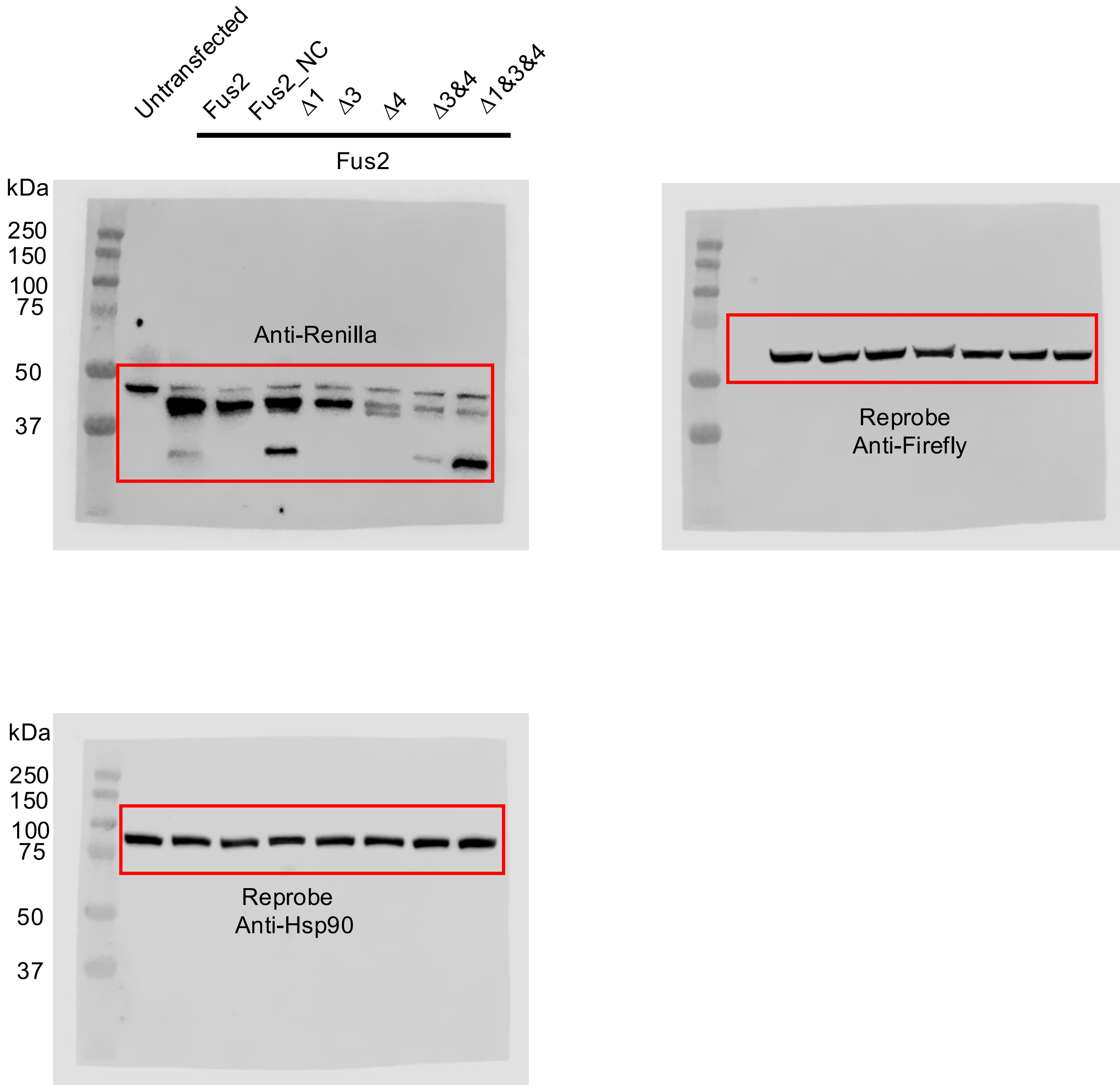

Supplemental Figure 9D

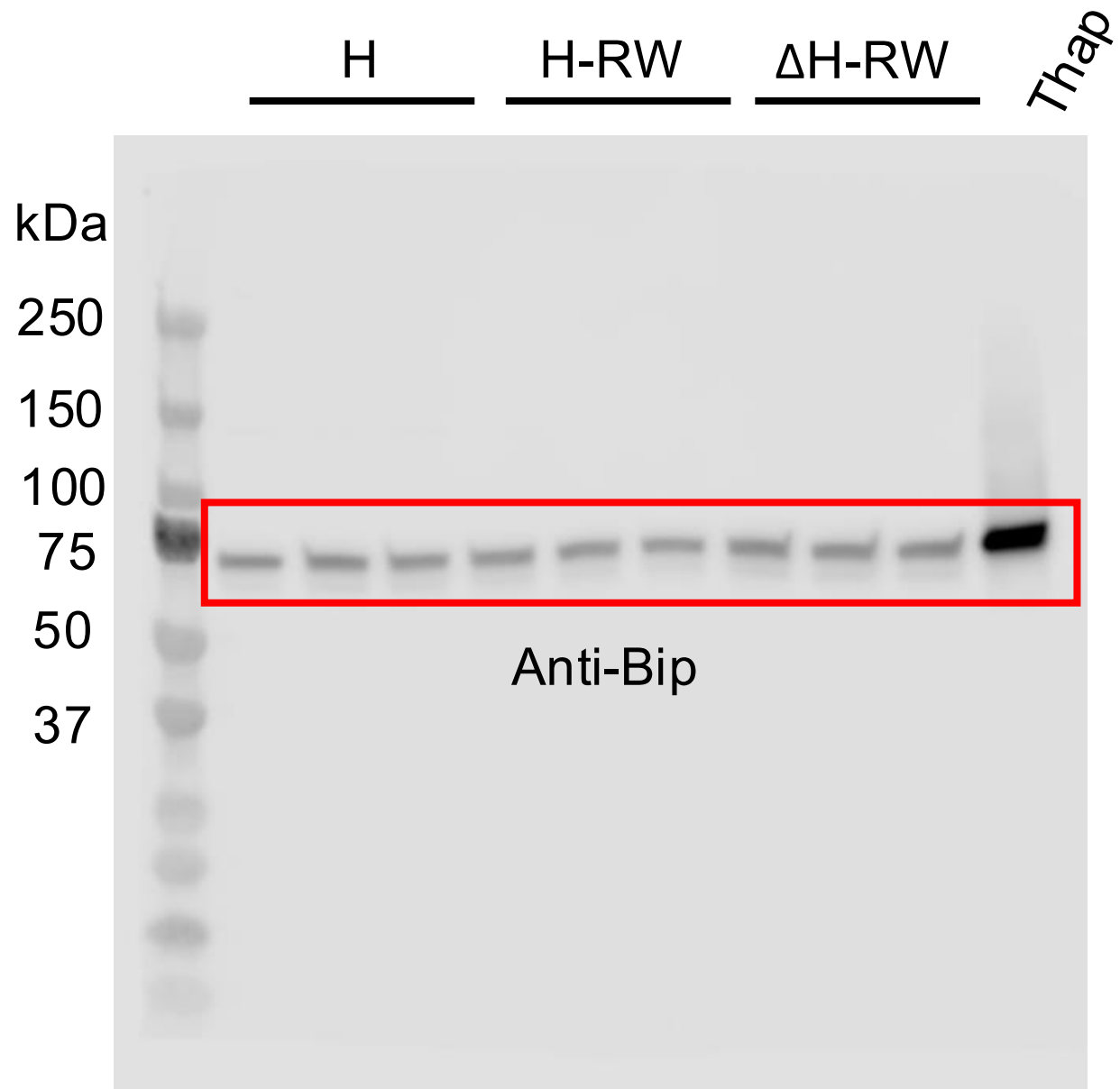

Supplement: Supplementary file 2 [file jci-136-203177-s280.pdf]
